# Recognition of pathogenic bacteria by intestinal stem cells promotes adult *Drosophila* midgut regeneration

**DOI:** 10.1371/journal.pbio.3003951

**Published:** 2026-08-25

**Authors:** Bhavin Uttekar, Marie Srotyr, Sanket Sudam Ravale, Liliana Ewertowska, Rebecca Wafer, Martina Legido, Parthive H. Patel

**Affiliations:** School of Biochemistry and Cellular and Molecular Medicine, Faculty of Health and Life Sciences, University of Bristol, Bristol, United Kingdom; University of Birmingham, UNITED KINGDOM OF GREAT BRITAIN AND NORTHERN IRELAND

## Abstract

When enteropathogenic bacteria breach the intestinal epithelium, they are recognised by epithelial and immune cells that elicit an intestinal regenerative response. However, less is known about whether and how intestinal stem cells (ISCs) directly detect invading pathogenic bacteria and couple this to their proliferation. Here, we show that adult *Drosophila* midgut ISCs recognise pathogenic bacteria through the peptidoglycan recognition proteins, PGRP-LC and PGRP-LE, and translate this into their proliferation by stimulating Imd-Mkk3-p38 signalling. Moreover, we find that PGRP-LC/LE-Imd-Mkk3-p38 signalling in ISCs regulates p38 activation throughout the midgut epithelium after infection, indicating that ISCs can influence the regenerative microenvironment in a non-cell autonomous manner. Whilst it was previously thought that ISC proliferation in both mammals and flies is driven solely by damage-induced signals after infection, our work reveals that ISCs can directly recognise pathogenic bacteria and mount a strong parallel regenerative response that spreads throughout the midgut epithelium. Increased ISC proliferation after bacterial recognition may also serve as a strategy to repopulate the epithelium with uninfected cells.

## Abstract 2

Bacteria infecting the intestinal epithelium are recognized by epithelial and immune cells, however the role of intestinal stem cells (ISCs) remains unclear. This study shows that adult Drosophila midgut ISCs recognize pathogenic bacteria via peptidoglycan recognition proteins promoting regeneration.

## Introduction

The inability to properly maintain or regenerate an epithelium leads to a breakdown of its integrity, structure and function, ultimately disrupting organismal homeostasis [1,2]. Maintenance of the mammalian intestinal epithelium relies on intestinal stem cells (ISCs), which generate differentiated absorptive and secretory epithelial cells [3]. In response to intestinal damage, ISCs receive proliferative signals, including epidermal growth factor (EGF), cytokines, and Wnts to replenish lost cells [4,5]. These signals originate from a regenerative microenvironment composed of resident epithelial cells, immune cells, mesenchyme, vasculature, and enteric glial cells [4,5].

Enteropathogenic bacterial infections cause intestinal damage. When enteropathogenic bacteria eventually breach the intestinal epithelial barrier, they are recognised by intestinal epithelial and innate immune cells [6]. This results in cytokine production and inflammation that supports a regenerative response within the intestinal epithelium [6]. While much is known about how intestinal epithelial and immune cells sense invading pathogenic bacteria, we know little about whether ISCs or intestinal progenitors sense bacteria during infection and mount a regenerative response.

Here we address this question using *Drosophila* as a model to study the response of midgut progenitors to bacterial intestinal infections. Like the mammalian intestine, the adult *Drosophila* midgut epithelium is maintained by ISCs that generate two primary epithelial cell types: absorptive enterocytes (ECs) and secretory enteroendocrine cells (EEs) [7–9]. In approximately 90-95% of cases, ISCs divide to produce an enteroblast (EB)—the precursor for ECs [8,10,11]. When EBs receive a strong Notch signal from ISCs, they differentiate into ECs [10,12]. In contrast, EEs arise either from ISCs via an enteroendocrine precursor (EEp) or through direct differentiation of ISCs [13]. ISCs and their daughters, EBs and EEps, are collectively considered adult midgut progenitors and all express the progenitor marker *escargot* (*esg*) [7]. Together with the visceral muscle and trachea that surround the midgut epithelium, EBs, ECs, and EEs form a regenerative microenvironment that facilitates midgut repair by producing signals that directly promote ISC proliferation [8,14,15]. These cues include Unpaired (Upd1-3) cytokines, EGFs, Wnt, Hedgehog, fibroblast growth factor (FGF) and protein tyrosine kinase 7 (PTK7)/Off-track [14–23].

Oral infection of adult flies with gram-negative pathogenic bacteria such as *Pseudomonas entomophila* (*P.e.*) or *Erwinia carotovora carotovora 15* (*Ecc15*) causes midgut damage [19,24–26]. In the case of *P.e*., this is due to the expression of virulence factors that directly cause midgut damage, especially the pore-forming toxin, Monalysin [27,28]. Damage to the midgut epithelium induces a regenerative microenvironment that stimulates ISC proliferation [19,29]. Nevertheless, it is unknown whether bacterial recognition by midgut cell types plays a direct role in the regenerative response. Similarly, the human intestine can be infected by gram-negative pathogenic bacteria that damage epithelial cells, including *Escherichia coli*, *Shigella flexneri* and *Salmonella enterica* [30]. While commensal bacterial recognition by ISCs has been suggested to promote intestinal homeostasis [31,32], it is unknown whether our intestines, particularly the ISCs, sense invading pathogenic bacteria and mount a regenerative response.

In the adult *Drosophila* midgut, ECs can recognise invading pathogenic bacteria (*Ecc15*) by sensing peptidoglycan, a component of the bacterial cell wall, through peptidoglycan recognition proteins (PGRPs) [33]. Peptidoglycan is a crosslinked polymer, with structural variations distinguishing gram-positive (L-lysine (Lys)-type) and gram-negative (meso-diaminopimelic acid (DAP)-type) bacteria [34–36]. *Drosophila* PGRPs are classified by size into short extracellular forms (PGRP-S) and long forms (PGRP-L) that can be intracellular or transmembrane [34,36]. The long forms of PGRP (PGRP-LC and PGRP-LE) detect DAP-type peptidoglycan mainly from gram-negative bacteria and activate antimicrobial peptide (AMP) production via the immune deficiency (Imd) pathway [36–38]. Further, both PGRP-LC and PGRP-LE are important in ECs of the posterior adult midgut for AMP production after *Ecc15* infection [37,38]. In the adult fly midgut, p38 stress-activated protein kinase (SAPK) signalling in ECs is important for promoting damage-induced ISC proliferation and has also been proposed to contribute to innate immune responses after pathogenic bacterial infection [33,39]. Damage activates p38 signalling in ECs via Nicotinamide adenine dinucleotide phosphate (NADPH) oxidase (Nox)-Apoptosis signal-regulating kinase 1 (Ask1)-Mitogen-activated protein kinase kinase 3 (Mkk3) signalling, which promotes *upd3* expression by ECs and ISC proliferation [39]. In general, activation of p38 occurs through dual phosphorylation by the stress-activated protein kinase kinase (SAP2K) Mkk3/Licorne, which is regulated by stress-activated protein kinase kinase kinases (SAP3Ks) such as Ask1, Mitogen-activated protein kinase kinase kinase 1 (Mekk1) and Transforming growth factor-β-activated kinase 1 (Tak1) [40]. Further, *Ecc15* peptidoglycan can activate p38 signalling in ECs through Imd-Mekk1-Mkk3 signalling [33]. p38 signalling in ECs promotes the expression of the NADPH oxidase, Dual oxidase (Duox), resulting in increased reactive oxygen species (ROS) production [33]. This increased ROS production eliminates invading pathogenic bacteria from the lumen [33,41]. Therefore, peptidoglycan sensing by ECs is deemed to elicit two strategies—ROS and AMP production—to eliminate invading pathogenic bacteria from the lumen. However, recent evidence suggests that ROS production by Duox does not eliminate pathogenic bacteria within midguts after infection [42]. Although it was hypothesised that ROS production triggered by *Ecc15* peptidoglycan sensing in ECs indirectly causes collateral epithelial damage and ISC proliferation, more recent evidence does not support this model [43,44]. Thus, it remains unknown whether midgut cell types, particularly ISCs, can directly recognise peptidoglycan to contribute to regeneration.

In mammals, most peptidoglycan sensing by cells likely occurs through the intracellular receptors, nucleotide-binding oligomerisation domain-containing proteins 1 and 2 (NOD1 and NOD2) [35]. Their functional equivalent in *Drosophila* is PGRP-LE [36,37]. NOD1 senses gram-negative bacteria, whilst NOD2 senses gram-positive and gram-negative bacteria [35]. Both NOD1 and NOD2 interact with the receptor-interacting serine/threonine protein kinase 2 (RIPK2), activating either nuclear factor-kappaB (NF-κB), or mitogen-activated protein kinase kinase kinase 7 (TAK1/MAP3K7) and mitogen-activated protein kinase (c-Jun N-terminal kinase (JNK), p38) signalling to stimulate cytokine production [35]. NOD2 has been shown to be expressed at higher levels in leucine-rich repeat-containing G protein-coupled receptor 5-positive (Lgr5^+^) stem cells within intestinal organoids. Treatment of intestinal organoids with the NOD2 ligand muramyl dipeptide—a component of bacterial peptidoglycan—produces a higher yield of intestinal organoids, suggesting that bacterial sensing could promote ISC survival and intestinal homeostasis [31]. Further, NOD2 was found to be critical for the regeneration of ISCs and intestinal epithelial restitution following doxorubicin-induced ISC and crypt cell depletion [31]. This study, however, did not examine a pathophysiological in vivo role for NOD2 in intestinal regeneration during pathogenic bacterial infection, where ISCs are spared.

Here we explore if and how adult *Drosophila* midgut progenitors recognise pathogenic bacteria after infection and translate this into a regenerative response. We find that PGRP-LC/LE-Imd-Mkk3-p38 signalling is required within ISCs for adult *Drosophila* midgut regeneration after pathogenic bacterial infection. Mechanistically, PGRP-LC and PGRP-LE in ISCs recognise pathogenic bacteria and translate this into their proliferation by activating Mkk3-p38 signalling. Furthermore, we show that PGRP-LC/LE-Imd-Mkk3-p38 signalling in ISCs activates p38 throughout the epithelium after infection, indicating that progenitors can shape the regenerative microenvironment in a non-cell autonomous manner to support epithelial regeneration. While ISC proliferation after intestinal infection by pathogenic bacteria has been thought to be solely due to midgut damage, our work shows that ISC proliferation is also strongly induced by direct pathogen recognition by ISCs.

## Results

### PGRP-LC and PGRP-LE promote ISC proliferation and p38 activation in the midgut epithelium after pathogenic bacterial infection

**Fig 1:**
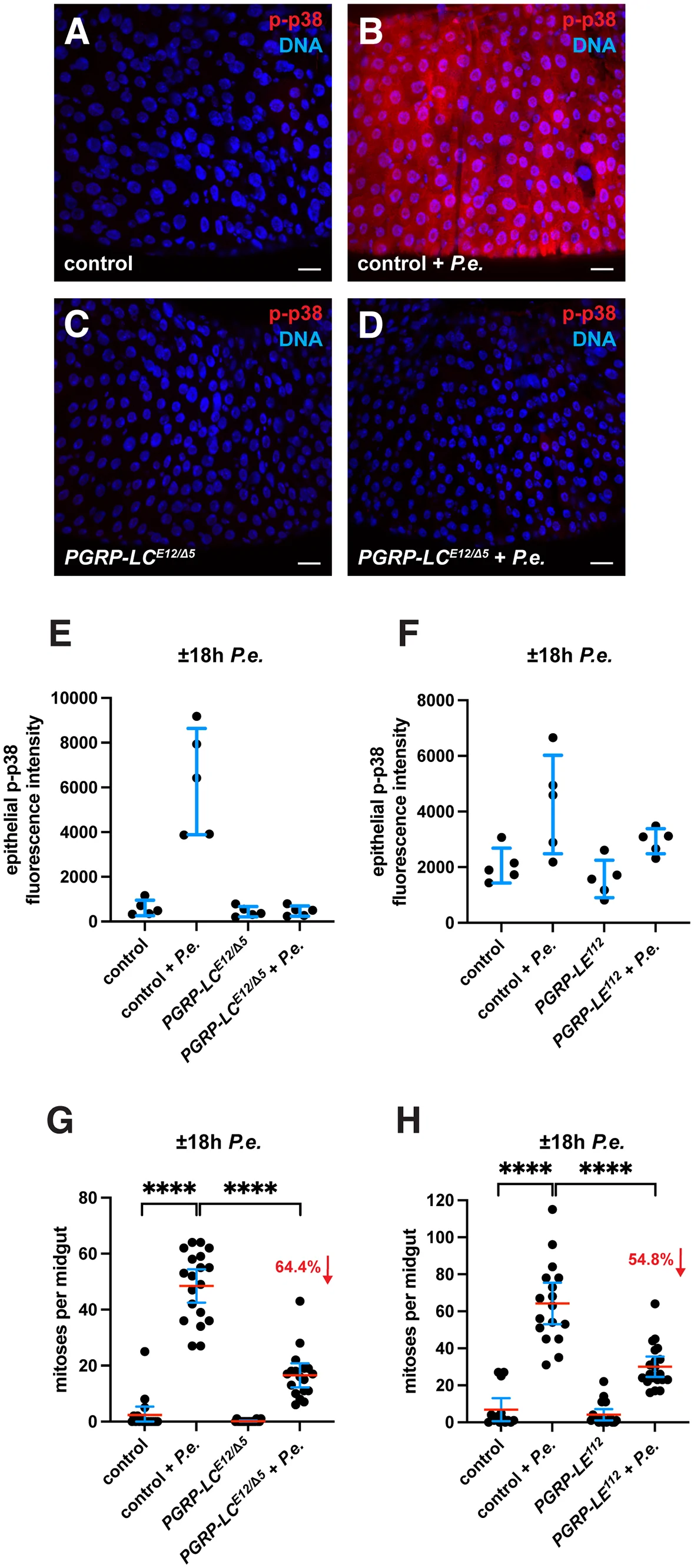
PGRP-LC and PGRP-LE promote ISC proliferation and p38 activation in the midgut epithelium after pathogenic bacterial infection. Both PGRP-LC and PGRP-LE are required for p38 activation in the midgut epithelium after *P.e.* infection **(A–F)**. p-p38 (red) increased in infected control midguts **(B)** compared to uninfected control midguts **(A)**; however, decreased in infected *PGRP-LC* transheterozygote (*PGRP-LC*^*E12*^*/PGRP-LC*^*Δ5*^) midguts **(D)** compared to infected control midguts **(B)**. p-p38 levels were similar between uninfected control midguts **(A)** and uninfected *PGRP-LC*^*E12*^*/PGRP-LC*^*Δ5*^ midguts **(C)**. The epithelial p-p38 fluorescence intensity increased in infected control midguts (*n* = 5 from one of two experiments) compared to uninfected control midguts (*n* = 5 from one of two experiments). The epithelial p-p38 fluorescence intensity decreased in infected midguts from *PGRP-LC* transheterozygotes (*n* = 5 from one of two experiments) compared to infected control midguts **(E)**. The epithelial p-p38 fluorescence intensity increased in infected control midguts (*n* = 5 from one of two experiments) compared to uninfected control midguts (*n* = 5 from one of two experiments). The epithelial p-p38 fluorescence intensity decreased in infected *PGRP-LE*^*112*^ midguts (*n* = 5 from one of two experiments) compared to infected control midguts **(F)**. Both PGRP-LC and PGRP-LE are required for ISC proliferation after *P.e.* infection **(G-H)**. The mean mitoses per midgut increased in infected control midguts (*n* = 19 from one of two experiments) compared to uninfected control midguts (*n* = 18 from one of two experiments) (Mann–Whitney; *p* < 0.0001). The mean mitoses per midgut decreased in infected midguts from *PGRP-LC* transheterozygotes (*n* = 18 from one of two experiments) compared to infected control midguts (Mann–Whitney; *p* < 0.0001) **(G)**. The mean mitoses per midgut increased in infected control midguts (*n* = 17 from one of two experiments) compared to uninfected control midguts (*n* = 14 from one of two experiments) (Mann–Whitney; *p* < 0.0001). The mean mitoses per midgut decreased in infected *PGRP-LE*^*112*^ midguts (*n* = 20 from one of two experiments) compared to infected control midguts (Mann–Whitney; *p* < 0.0001) **(H)**. Data in A-F, acquired from R4a-b region. In G and H, mean (red line) and 95% confidence intervals (blue bars) are shown. In E and F, standard deviation (blue bars) is shown. DNA is in blue. Scale bars are 20 µm. Related supplementary data for this figure can be found in S1 Fig and S1 Data.

Previously, it was shown that PGRP-LC is required for p38 activation in the midgut epithelium after exposure to peptidoglycan from *Ecc15* [33]. Further, it was also shown that p38 is activated in the midgut epithelium after *P.e.* infection [39]. Thus, we tested whether PGRP-LC was required for p38 activation after *P.e.* infection. We observed that null *PGRP-LC*^*E12*^ mutants were not viable as homozygotes on our medium, whereas the putative null *PGRP-LC*^*Δ5*^ mutants were viable. Thus, we examined p38 activation in *PGRP-LC*^*E12*^/*PGRP-LC*^*Δ*5^ transheterozygotes. Using an antibody against phosphorylated human p38 (Thr180/Tyr182) (p-p38) [39], we found that PGRP-LC transheterozygotes showed a decrease in p38 activation following continuous *P.e.* infection for 18 hours compared to infected control midguts (Fig 1A–1E). The increased phosphorylated p38 in infected control midguts likely represents both activated p38a and p38b [39]. We then determined whether the intracellular peptidoglycan sensor, PGRP-LE, was necessary for p38 activation in the midgut epithelium after *P.e.* infection. Indeed, p38 activation in the midgut epithelium also decreased in infected null *PGRP-LE*^*112*^ mutants compared to infected control midguts (Figs 1F and S1A–S1D). These data suggest that peptidoglycan recognition by midgut epithelial cells through PGRP-LC and PGRP-LE promotes p38 activation in the midgut epithelium. Previously, it was shown that p38 activity in ECs is required for ISC proliferation after *P.e.* infection [39]. Thus, we determined whether PGRP-LC and PGRP-LE were both required for ISC proliferation after *P.e.* infection. ISC proliferation decreased by 64.4% and 54.8% in *PGRP-LC* transheterozygotes (*PGRP-LC*^*E12*^/*PGRP-LC*^*Δ5*^) and *PGRP-LE*^*112*^ mutants after *P.e.* infection, respectively, compared to infected control midguts (Fig 1G–1H). Interestingly, infected *PGRP-LC*^*Δ5*^ mutants showed only a 23.7% decrease in ISC proliferation compared to infected control midguts (S1E Fig), suggesting that *PGRP-LC*^*Δ5*^ [45] is a hypomorphic allele of *PGRP-LC*. Together, these data indicate that PGRP-LC and PGRP-LE are required to promote midgut regeneration after pathogenic bacterial infection, partly by inducing p38 activity in the midgut epithelium.

### Pathogenic bacterial recognition by progenitors stimulates ISC proliferation via p38 signalling

Whilst both PGRP-LC and PGRP-LE were necessary for ISC proliferation following pathogenic bacterial infection (Fig 1G–1H), the specific cell type in which they functioned remained unclear. Since PGRP-LC was previously shown to be required for p38 activation in ECs after exposure to peptidoglycan from *Ecc15* and to promote antimicrobial peptide production [33,37,38], we first tested whether PGRP-LC was required in ECs to promote ISC proliferation after pathogenic bacterial infection. Depleting *PGRP-LC* mRNA by RNA interference (*RNAi*) in ECs with the EC-specific driver *mex-GAL4; tubGAL80*^*ts*^ (*Malic enzyme modifier*, *mex*^*ts*^) for 5 or 6 days did not affect ISC proliferation after continuous *P.e.* infection for 18 hours (S2A and S2C Fig), suggesting that PGRP-LC function in ECs is not essential for promoting midgut regeneration.

**Fig 2:**
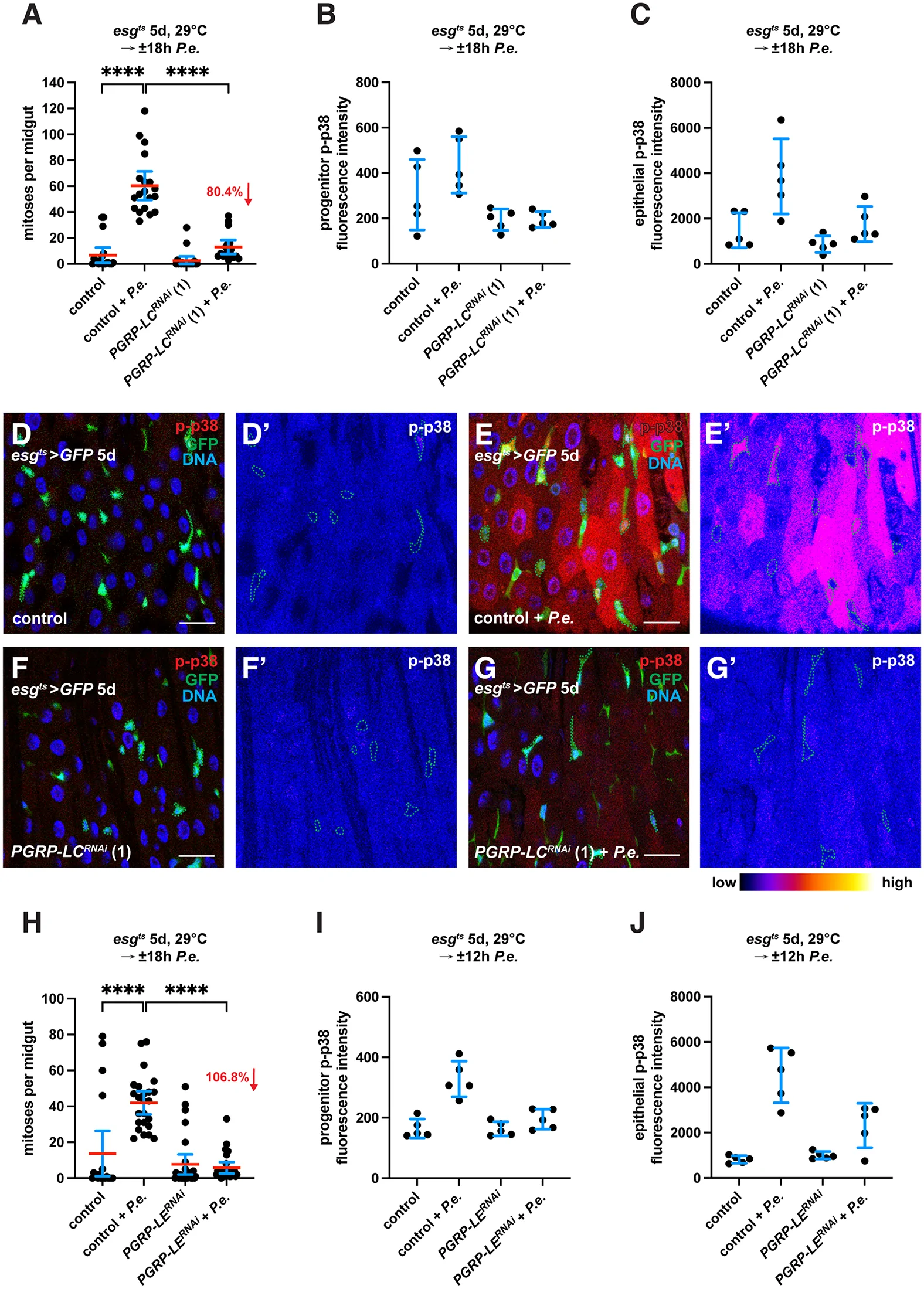
Pathogenic bacterial recognition by progenitors via PGRP-LC and PGRP-LE stimulates ISC proliferation and p38 activation throughout the midgut epithelium. PGRP-LC in progenitors promotes ISC proliferation after *P.e.* infection **(A)**. The mean number of mitoses per midgut increased in infected control midguts (*n* = 19 from one of two experiments) compared to uninfected control midguts (*n* = 19 from one of two experiments) (Mann–Whitney; *p* < 0.0001). The mean number of mitoses decreased in infected midguts expressing *PGRP-LC*^*RNAi*^ (1) in progenitors (*n* = 17 from one of two experiments) compared to infected control midguts (Mann–Whitney; *p* < 0.0001) **(A)**. PGRP-LC in progenitors stimulates p38 activation in progenitors and throughout the midgut epithelium after *P.e.* infection **(B–G)**. Progenitor p-p38 fluorescence intensity increased in infected control midguts (*n* = 5 from one of two experiments) compared to progenitors in uninfected control midguts (*n* = 5 from one of two experiments). Progenitor p-p38 fluorescence intensity decreased in infected midguts expressing *PGRP-LC*^*RNAi*^ (1) with *esg*^*ts*^ (*n* = 5 from one of two experiments) compared to progenitors in infected control midguts **(B)**. The epithelial p-p38 fluorescence intensity per midgut from those analysed in B are shown in C. The epithelial p-p38 fluorescence intensity increased in *P.e.-*infected control midguts compared to uninfected control midguts. The epithelial p-p38 fluorescence intensity decreased in infected midguts expressing *PGRP-LC*^*RNAi*^ (1) in progenitors compared to infected control midguts **(C)**. p-p38 (D, E: red; D′, E′: Fire LUT) levels increased in progenitors (E: GFP; E–E′: green-dashed outline) and throughout the midgut epithelium after *P.e.* infection **(E-E’)** compared to uninfected control midguts **(D–D′)**. p-p38 levels decreased (G: red; G′: Fire LUT) in progenitors and throughout the midgut epithelium in infected midguts expressing *PGRP-LC*^*RNAi*^ (1) in progenitors **(G–G′)** compared to *P.e*.-infected control midguts **(E–E′)**. PGRP-LE in progenitors promotes ISC proliferation after *P.e.* infection **(H)**. The mean number of mitoses per midgut increased in infected control midguts (*n* = 24 from one of two experiments) compared to uninfected control midguts (*n* = 20 from one of two experiments) (Mann–Whitney; *p* < 0.0001). The mean number of mitoses per midgut decreased in infected midguts expressing *PGRP-LE*^*RNAi*^ in progenitors (*n* = 25 from one of two experiments) compared to infected control midguts (Mann–Whitney; *p* < 0.0001) **(H)**. PGRP-LE in progenitors stimulates p38 activation in progenitors and throughout the midgut epithelium after *P.e.* infection **(I–J)**. Progenitor p-p38 fluorescence levels increased in infected control midguts (*n* = 5 from one of two experiments) compared to progenitors in uninfected control midguts (*n* = 5 from one of two experiments). Progenitor p-p38 levels decreased in infected midguts expressing *PGRP-LE*^*RNAi*^ with *esg*^*ts*^ (*n* = 5 from one of two experiments) compared to progenitors in infected control midguts **(I)**. The epithelial p-p38 fluorescence intensity per midgut from those analysed in I are shown in J. The epithelial p-p38 fluorescence intensity increased in infected control midguts compared to uninfected control midguts. The epithelial p-p38 fluorescence intensity decreased in infected midguts expressing *PGRP-LE*^*RNAi*^ in progenitors compared to infected control midguts **(J)**. Data in B–G′ and I–J, acquired from R4a-b region. In A and H, mean (red line) and 95% confidence intervals (blue bars) are shown. In B–C and I–J, standard deviation (blue bars) is shown. DNA is in blue. Scale bars are 20 µm. Related supplementary data for this figure can be found in S2 Fig and S1 Data.

Previously, it was shown that PGRP-LC is highly expressed in midgut progenitor cells [46]. Additionally, ISC proliferation in *Drosophila* is known to increase with age, and both PGRP-LC and PGRP-LE are required for this age-related increase [46]. Thus, we next tested whether PGRP-LC and PGRP-LE were both required in progenitors. We found that both receptors were required in progenitors for ISC proliferation after *P.e.* infection. Depletion of *PGRP-LC* in progenitors with the progenitor-specific driver *esg-GAL4 tubGAL80*^*ts*^ (*esg*^*ts*^) reduced ISC proliferation after *P.e.* infection by 80.4% and 49.5% with *RNAi* (1) and *RNAi* (2), respectively, compared to infected control midguts (Figs 2A and S2B). Similarly, we found that *PGRP-LE* mRNA depletion resulted in a 106.8% decrease in ISC proliferation after *P.e.* infection compared to infected control midguts (Figs 2H and S2D). Furthermore, the decrease in ISC proliferation due to *PGRP-LC* and *PGRP-LE* depletion in progenitors was not due to a decrease in progenitor number as the mean percent progenitors in these midguts was similar to uninfected and infected control midguts (S2I–S2J Fig). Together, these data suggest that DAP-type peptidoglycan recognition through PGRP-LC and PGRP-LE in progenitors stimulates ISC proliferation after *P.e.* infection.

Since both PGRP-LC and PGRP-LE were required for p38 activation in the midgut epithelium after *P.e.* infection, we tested whether both PGRPs were required for p38 activation in *esg*^*+*^ progenitors. We found increased levels of activated p38 within *esg*^*+*^ progenitors in addition to ECs after *P.e.* infection compared to the basal p-p38 levels in uninfected control midguts (Fig 2B–2E′). However, we found that *PGRP-LC* depletion with *esg*^*ts*^ reduced p38 activation in progenitors by 105.0% after *P.e.* infection compared to progenitors in infected control midguts (Fig 2B and 2D–2G′; S1A Appendix). Similarly, *PGRP-LE* depletion with *esg*^*ts*^ reduced p38 activation in progenitors by 74.2% after *P.e.* infection compared to progenitors in infected control midguts (Figs 2I and S2E–S2H′; S1B Appendix). Moreover, we surprisingly found that depleting *PGRP-LC* and *PGRP-LE* in progenitors using *esg*^*ts*^ reduced p38 activation throughout the midgut epithelium after *P.e.* infection compared to infected control midguts (Figs 2C–2G′, 2J, and S2E–S2H′). These data suggest that pathogenic bacterial recognition by progenitors through PGRP-LC and PGRP-LE promotes ISC proliferation, possibly through activation of p38 signalling in progenitors and throughout the midgut epithelium after *P.e.* infection.

We next ascertained whether other gram-negative pathogenic bacteria such as *Ecc15* can promote ISC proliferation and activate p38 in the midgut epithelium through PGRP-LC in progenitors. We found that *Ecc15* can stimulate ISC proliferation, as previously described [25], as well as p38 activation in the epithelium of control midguts (S2K–S2L Fig). However, depleting *PGRP-LC* in progenitors inhibited ISC proliferation and p38 activation throughout the midgut epithelium (S2K–S2L Fig). These data indicate that the mechanism by which pathogenic bacterial recognition by progenitors via PGPR-LC and PGRP-LE promotes ISC proliferation is not limited to specific pathogenic bacteria.

**Fig 3:**
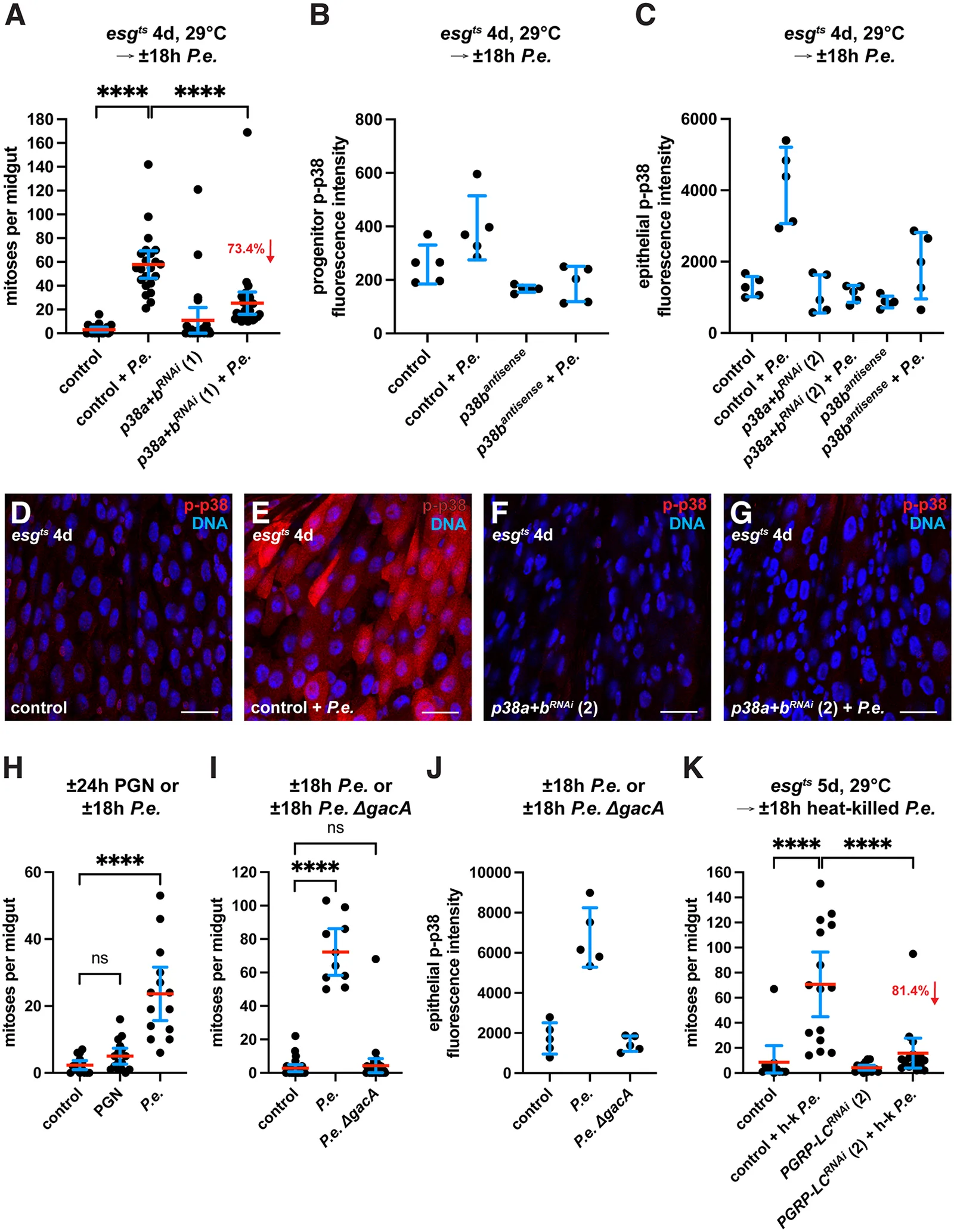
Peptidoglycan alone does not trigger p38-mediated ISC proliferation. p38 activity in progenitors promotes ISC proliferation after *P.e.* infection **(A)**. The mean mitoses per midgut increased in infected control midguts (*n* = 22 pooled from three experiments) compared to uninfected control midguts (*n* = 21 pooled from three experiments) (Mann–Whitney; *p* < 0.0001). The mean mitoses per midgut decreased in infected midguts expressing *p38a+b*^*RNAi*^ (1) in progenitors (*n* = 34 pooled from three experiments) compared to infected control midguts (Mann–Whitney; *p* < 0.0001) **(A)**. p38 activity in progenitors stimulates p38 activation throughout the midgut epithelium after *P.e.* infection **(B–G)**. Progenitor p-p38 fluorescence intensity in infected control midguts (*n* = 5 from one experiment) increased compared to uninfected control midguts (*n* = 5 from one experiment). Progenitor p-p38 fluorescence intensity in infected midguts expressing *p38b*^*antisense*^ with *esg*^*ts*^ (*n* = 5 from one experiment) decreased compared to infected control midguts **(B)**. Epithelial p-p38 fluorescence intensity increased in infected control midguts (*n* = 5 from one experiment) compared to uninfected control midguts (*n* = 5 from one experiment). Epithelial p-p38 fluorescence intensity decreased in infected midguts with progenitors expressing *p38a+b*^*RNAi*^ (2) or *p38b*^*antisense*^ (*n* = 5 from one experiment) compared to infected control midguts **(C)**. Epithelial p38 activation (red) increased in infected control midguts (E) compared to uninfected control midguts **(D)**. Epithelial p38 activation was reduced in infected midguts with progenitors expressing *p38a+b*^*RNAi*^ (2) (G) compared to infected control midguts **(E)**. Epithelial p38 activation in uninfected midguts expressing *p38a+b*^*RNAi*^ (2) in progenitors (F) was similar to uninfected control midguts **(D)**. DAP-type peptidoglycan and non-virulent *P.e.* cannot stimulate ISC proliferation or epithelial p38 activation **(H–J)**. Mean mitoses per midgut did not increase upon feeding DAP-type peptidoglycan (*n* = 16 from one of two experiments) like after *P.e.* infection (*n* = 14 from one experiment) (Mann–Whitney; *p* < 0.0001) compared to control midguts (*n* = 15 from one of two experiments) (Mann–Whitney; *p* = 0.0675) **(H)**. Mean mitoses per midgut did not increase upon infection with non-virulent *P.e.* Δ*gacA* (*n* = 17 from one of two experiments) (Mann–Whitney; *p* = 0.4645) like after infection with virulent *P.e.* (*n* = 10 from one experiment) (Mann–Whitney; *p* < 0.0001) compared to control midguts (*n* = 17 from one of two experiments) **(I)**. Epithelial p-p38 fluorescence intensity did not increase after infection with non-virulent *P.e. ΔgacA* (*n* = 5 from one of two experiments) like after infection with virulent *P.e.* (n = 5 from one of two experiments) compared to control midguts (*n* = 5 from one of two experiments) **(J)**. PGRP-LC in progenitors promotes ISC proliferation in midguts exposed to heat-killed *P.e.* **(K)**. Mean mitoses per midgut increased in flies fed heat-killed **(h–k)** *P.e.* (*n* = 15 from one experiment) compared to control midguts (*n* = 11 from one experiment) (Mann–Whitney; *p* < 0.0001). The mean mitoses per midgut decreased in midguts expressing *PGRP-LC*^*RNAi*^ (2) in progenitors exposed to heat-killed *P.e.* (n = 16 from one experiment) compared to control flies fed heat killed *P.e.* (Mann–Whitney; *p* < 0.0001) **(K)**. Data in B-G and J, acquired from R4a-b region. In A, H–I and K, mean (red line) and 95% confidence intervals (blue bars) are shown. In B–C and J, standard deviation (blue bars) is shown. DNA is in blue. Scale bars are 20 µm. Related supplementary data for this figure can be found in S3 Fig and S1 Data.

Since p38 activation within progenitors and ISC proliferation after *P.e.* infection required both *PGRP-LC* and *PGRP-LE*, we next tested whether p38 signalling was required in progenitors for ISC proliferation after *P.e.* infection. To do this, we depleted p38 signalling from midgut progenitors by expressing an *RNAi* that targets both *p38a* and *p38b* (*p38a+b*^*RNAi*^) using *esg*^*ts*^ and infected these flies with *P.e.*. Depleting *p38a* and *p38b* in progenitors resulted in a 73.4% decrease in ISC proliferation after *P.e.* infection compared to infected control midguts (Fig 3A). This decrease was not caused by reduced progenitor number due to *p38a and p38b* depletion with *esg*^*ts*^ and *P.e.* infection as the mean percent *esg*^*+*^ progenitors in these midguts was similar to uninfected and *P*.*e*.-infected control midguts (S3A Fig). These data suggest that p38 signalling in progenitors is required for ISC proliferation after *P.e.* infection.

We then examined whether depleting p38 in progenitors affects their levels of activated p38 after *P.e.* infection. Depletion of p38b resulted in an 87.8% decrease in p38 activation in progenitors after infection compared to progenitors in infected control midguts (Figs 3B and S3B; S1C Appendix). Surprisingly, we also found that depleting *p38a* and *p38b* or p38b alone in progenitors with *esg*^*ts*^ reduced p38 activation throughout the midgut epithelium after *P.e.* infection (Fig 3C–3G). These data indicate that in addition to regulating ISC proliferation, p38a and p38b activity in progenitors non-cell autonomously controls p38 activity throughout the midgut epithelium.

### DAP-type peptidoglycan alone is insufficient to trigger an ISC regenerative response in healthy midguts

Since peptidoglycan from *Ecc15* was shown to activate p38 in the midgut epithelium [33], we tested whether DAP-type peptidoglycan alone could stimulate ISC proliferation in uninfected midguts. We first determined whether midgut epithelial cells recognised DAP-type peptidoglycan after feeding using *Dipt(2.2)-lacZ*, which reports the expression of the AMP, *Diptericin*. As expected, DAP-type peptidoglycan or *P.e.* activated *Dipt(2.2)-lacZ* in ECs (S3D–S3F′ Fig). This indicates that after adult flies consumed food containing DAP-type peptidoglycan or *P.e.*, midgut ECs, as previously reported [37,38], launched an innate immune response by producing AMPs. Nevertheless, DAP-type peptidoglycan did not trigger ISC proliferation in these flies, while *P.e.* infection did (Fig 3H), indicating that DAP-type peptidoglycan alone cannot trigger an ISC regenerative response.

We further tested whether exposing the midgut epithelium to DAP-type peptidoglycan from non-virulent *P.e.* or heat-killed virulent *P.e.* could stimulate ISC proliferation. Non-virulent *P.e.* activated the *Dipt(2.2)-lacZ* reporter but did not stimulate ISC proliferation or epithelial p38 like *P.e.* (Figs 3I–3J and S3G–S3G′). Thus, DAP-type peptidoglycan released from non-virulent *P.e.* was sufficient to trigger AMP production but not ISC proliferation. We then fed flies heat-killed *P.e.* (at 95°C), which surprisingly triggered ISC proliferation (Figs 3K and S3C). These midguts appeared damaged after exposure to heat-killed *P.e.*, suggesting that certain virulence factors may be resistant to 95°C heat treatment. Interestingly, depleting *PGRP-LC* or *PGRP-LE* from progenitors decreased ISC proliferation by 81.4% and 82.1%, respectively, after feeding heat-killed *P.e.* compared to heat-killed *P.e.*-fed control midguts (Figs 3K and S3C).

Together, these data indicate that DAP-type peptidoglycan alone is not sufficient to stimulate ISC proliferation, and that it possibly requires midgut damage to stimulate ISC proliferation and activate epithelial p38. This is consistent with our experiments feeding damage-causing pathogenic bacteria such as *P.e.* and *Ecc15*.

### Imd signalling in progenitors promotes ISC proliferation and p38 activation in the midgut epithelium after pathogenic bacterial infection

**Fig 4:**
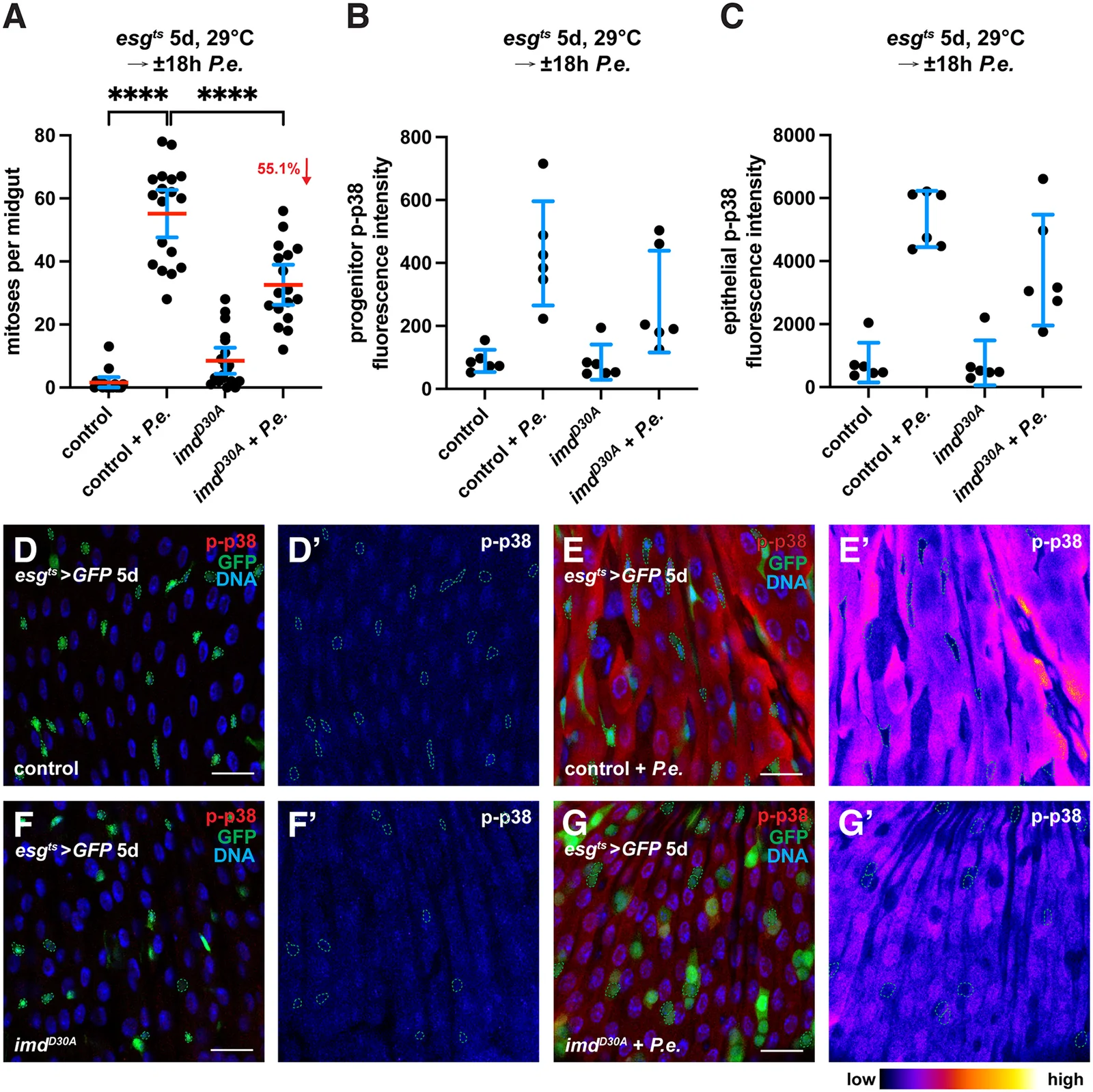
Imd signalling in progenitors stimulates ISC proliferation and p38 activation throughout the midgut epithelium after pathogenic bacterial infection. Imd signalling in progenitors promotes ISC proliferation after *P.e.* infection **(A)**. The mean number of mitoses per midgut increased in infected control midguts (*n* = 18 from one of two experiments) compared to uninfected control midguts (*n* = 17 from one of two experiments) (Mann–Whitney; *p* < 0.0001). The mean number of mitoses decreased in infected midguts expressing *imd*^*D30A*^ in progenitors (*n* = 17 from one of two experiments) compared to infected control midguts (Mann–Whitney; *p* < 0.0001) **(A)**. Imd signalling in progenitors promotes p38 activation in progenitors and throughout the midgut epithelium after *P.e.* infection **(B–G)**. Progenitor p-p38 fluorescence intensity increased in infected control midguts (*n* = 6 from one of two experiments) compared to progenitors in uninfected control midguts (*n* = 6 from one of two experiments). Progenitor p-p38 fluorescence intensity decreased in infected midguts expressing *imd*^*D30A*^ with *esg*^*ts*^ (*n* = 6 from one of two experiments) compared to progenitors in infected control midguts **(B)**. The epithelial p-p38 fluorescence intensity per midgut from those analysed in B are shown in C. The epithelial p-p38 fluorescence intensity increased in *P.e.-*infected control midguts compared to uninfected control midguts. The epithelial p-p38 fluorescence intensity decreased in infected midguts expressing *imd*^*D30A*^ in progenitors compared to infected control midguts **(C)**. p-p38 (D, E: red; D′, E′: Fire LUT) levels increased in progenitors (E: GFP; E–E′: green-dashed outline) and throughout the midgut epithelium after *P.e.* infection **(E, E′)** compared to uninfected control midguts **(D–D′)**. Expressing *imd*^*D30A*^ in progenitors inhibited the increase in p-p38 (G: red; G′: Fire LUT) in progenitors and throughout the midgut epithelium after *P.e.* infection **(G–G′)** compared to infected control midguts **(E–E′)**. Data in B–G′, acquired from R4a-b region. In A, mean (red line) and 95% confidence intervals (blue bars) are shown. In B–C, standard deviation (blue bars) is shown. DNA is in blue. Scale bars are 20 µm. Related supplementary data for this figure can be found in S4 Fig and S1 Data.

Previously, it was shown that Imd but not Relish (Rel) is required for p38 activation in the midgut epithelium after exposure to peptidoglycan from *Ecc15* [33]. Indeed, like PGRP-LC and PGRP-LE, Imd is also required in midgut progenitors for the age-related increase in ISC proliferation [46]. Thus, Imd may also be involved in progenitors to promote ISC proliferation and p38 activation in the midgut epithelium after infection. To test this, we first examined *imd*^*1*^ mutants but found that ISC proliferation after infection was similar between *imd*^*1*^ mutant and control midguts (S4A–S4B Fig). However, we then inhibited Imd specifically in progenitors by either expressing a dominant-negative form of Imd (Imd^D30A^) or by *RNAi*. Inhibiting Imd by either expressing Imd^D30A^ or *imd*^*RNAi*^ in progenitors reduced ISC proliferation by 55.1% and 83.5%, respectively (Figs 4A and S4C). This decrease was not caused by reduced progenitor number due to Imd^D30A^ expression with *esg*^*ts*^ and *P.e.* infection (S4E Fig). Further, we found a 43.9% decrease in p38 activation in progenitors expressing Imd^D30A^ after infection compared to progenitors in infected control midguts (Fig 4B and 4D–4G′; S2A Appendix). Moreover, we found a decrease in p38 activation in the midgut epithelium in *P.e.-*infected midguts expressing Imd^D30A^ or *imd*^*RNAi*^ in progenitors compared to infected control midguts (Figs 4C–4G′ and S4D). These data suggest that Imd is necessary in progenitors for p38 activation in the midgut epithelium and ISC proliferation. Finally, we tested using isogenic control and mutant lines whether Relish was required for ISC proliferation after *P.e.* infection. Consistent with a prior study using *Ecc15* [47], we found that ISC proliferation in *Rel*^*E20*^ mutant midguts was similar to control midguts after *P.e.* infection (S4F Fig). Together, these data suggest that Imd, but not Relish, is required in progenitors for ISC proliferation and p38 activation in the midgut epithelium.

### Mkk3-p38 signalling in progenitors promotes ISC proliferation and p38 activation in the midgut epithelium after pathogenic bacterial infection

**Fig 5:**
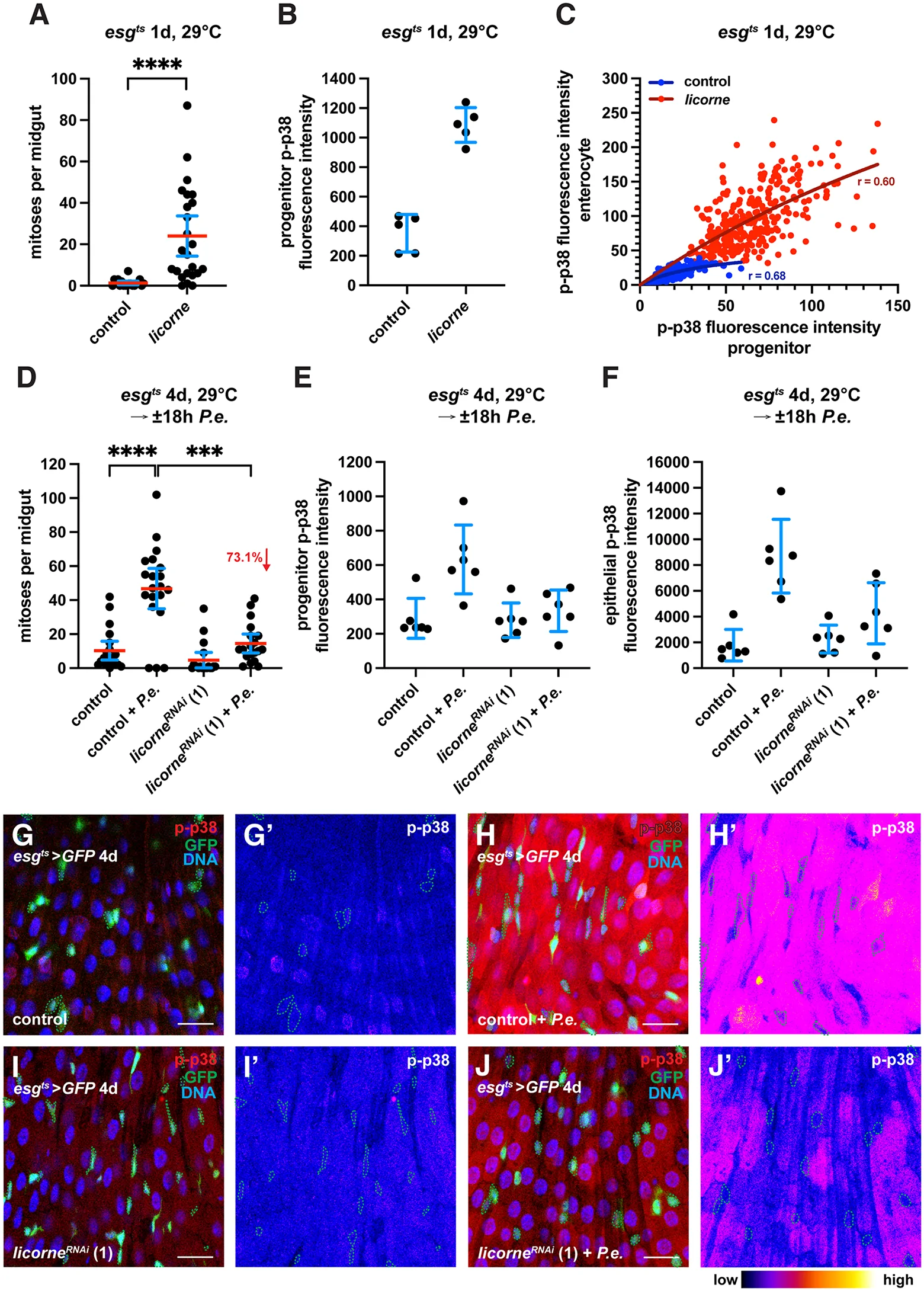
Mkk3/Licorne in progenitors promotes ISC proliferation and p38 activation throughout the midgut epithelium after pathogenic bacterial infection. Licorne overexpression in midguts promotes ISC proliferation **(A)**. The mean number of mitoses per midgut increased in midguts overexpressing *licorne* in progenitors (*n* = 24 from one of two experiments) compared to control midguts (*n* = 19 from one of two experiments) (Mann–Whitney; *p* < 0.0001) **(A)**. Licorne overexpression in progenitors activates p38 in progenitors and in nearby midgut epithelial cells **(B-C)**. Progenitor p-p38 fluorescence intensity increased in midguts overexpressing *licorne* with *esg*^*ts*^ (*n* = 5 from one experiment) compared to control midguts (*n* = 5 from one of two experiments) **(B)**. The p-p38 fluorescence intensity in progenitors correlated with p-p38 fluorescence intensity in adjacent enterocytes in control midguts (*n* = 247 cell pairs from three midguts) (Pearsons; *r =* 0.68). The p-p38 fluorescence intensity in progenitors correlated with p-p38 fluorescence intensity in adjacent enterocytes in midguts overexpressing *licorne* in progenitors (*n* = 310 cell pairs from three midguts) (Pearsons; *r =* 0.60) **(C)**. Licorne activity in progenitors promotes ISC proliferation after *P.e.* infection **(D)**. The mean number of mitoses per midgut increased in infected control midguts (*n* = 20 from one of two experiments) compared to uninfected control midguts (*n* = 20 from one of two experiments) (Mann–Whitney; *p* < 0.0001). The mean number of mitoses per midgut decreased in infected midguts expressing *licorne*^*RNAi*^ (1) in progenitors (*n* = 19 from one of two experiments) compared to infected control midguts (Mann–Whitney; *p* = 0.0001) **(D)**. Licorne activity in progenitors stimulates p38 activation in progenitors and throughout the midgut epithelium after *P.e.* infection **(E-J)**. The progenitor p-p38 fluorescence intensity increased in infected control midguts (*n* = 6 from one of two experiments) compared to uninfected control midguts (*n* = 6 from one of two experiments). The progenitor p-p38 fluorescence intensity decreased in infected midguts expressing *licorne*^*RNAi*^ (1) with *esg*^*ts*^ (*n* = 6 from one of two experiments) compared to infected control midguts **(E)**. The epithelial p-p38 fluorescence intensity per midgut from those analysed in E are shown in F. The epithelial p-p38 fluorescence intensity increased in *P.e.-*infected control midguts compared to uninfected control midguts. The epithelial p-p38 fluorescence intensity decreased in infected midguts expressing *licorne*^*RNAi*^ (1) in progenitors compared to infected control midguts **(F)**. p-p38 (G, H: red; G′, H′: Fire LUT) increases in progenitors (H: GFP; H–H′: green-dashed outline) and throughout the midgut epithelium after *P.e.* infection **(H–H′)** compared to uninfected control midguts **(G–G′)**. p-p38 (J: red; J′: Fire LUT) decreased in progenitors and throughout the midgut epithelium in midguts expressing *licorne*^*RNAi*^ (1) with *esg*^*ts*^ **(J–J′)** compared to infected control midguts **(H–H′)**. Data in B–C and E–J′, acquired from R4a-b region. In A and D, mean (red line) and 95% confidence intervals (blue bars) are shown. In B and E–F, standard deviation (blue bars) is shown. DNA is in blue. Scale bars are 20 µm. Related supplementary data for this figure can be found in S5 Fig and S1 Data.

Since PGRP-LC/LE-Imd-p38 signalling was required for ISC proliferation after *P.e.* infection, we next determined whether increasing p38 activity in midgut progenitors is sufficient to stimulate ISC proliferation. To activate p38 signalling within midgut progenitors, we overexpressed the *Drosophila* Mkk3, Licorne, with *esg*^*ts*^. Overexpression of Licorne in progenitors caused a significant increase in ISC proliferation after 1 day of expression (Fig 5A). Furthermore, we found that Licorne overexpression in progenitors for 1 day with *esg*^*ts*^ increased p38 activation by 211.1% within progenitors (Figs 5B and S5A–S5B′; S2B Appendix). Interestingly, we also found increased p38 activation in ECs adjacent to Licorne-overexpressing progenitors (S5A–S5B′ Fig), suggesting that p38 activation in progenitors causes p38 activation in ECs. To correlate p38 activation in progenitors with p38 activation in adjacent ECs, we measured the p-p38 fluorescence intensity in progenitors and the adjacent ECs within both control midguts and midguts with progenitors overexpressing Licorne. In midguts with progenitors overexpressing Licorne, we found a high correlation *(r =* 0.60) between p-p38 levels in progenitors and their adjacent ECs (Fig 5C). This suggests that increases in p38 activation in progenitors correspondingly increases p38 activation in adjacent ECs. In control midguts, we similarly found a high correlation (*r* = 0.68) between p-p38 levels in progenitors and in adjacent ECs (Fig 5C). This suggests that basal p38 activation in progenitors of unchallenged midguts regulates p38 activation in adjacent ECs. Together, these data show that overexpressing Mkk3 in progenitors can promote ISC proliferation and p38 activation in both progenitors and surrounding ECs.

Since Licorne overexpression stimulated ISC proliferation in the absence of *P.e.* infection, we next tested whether Licorne was required for ISC proliferation after *P.e.* infection. We found that depleting *licorne* with two *RNAi*s (*RNAi* (1) and *RNAi* (2)) in progenitors with *esg*^*ts*^ reduced ISC proliferation after *P.e.* infection (by 73.1% for *RNAi* (1)) compared to infected control midguts (Figs 5D and S5C). This decrease in ISC proliferation was not due to reduced progenitor number caused by *licorne* depletion with *esg*^*ts*^ and *P.e.* infection as the mean percent *esg*^*+*^ progenitors in these midguts was similar to uninfected and *P.e.*-infected control midguts (S5F–S5G Fig). These data suggest that Licorne, likely through p38a and p38b, promotes ISC proliferation after *P.e.* infection.

We next examined whether Mkk3/Licorne activity in progenitors regulated p38 activation in both the progenitors and throughout the midgut epithelium. We indeed found that depletion of *licorne* with *esg*^*ts*^ decreased p38 activation in both the progenitors and throughout the midgut epithelium. Depleting *licorne* in progenitors with two *RNAi*s (*RNAi*s (1) and (2)) decreased p38 activation within progenitors by 87.1% and 102.9%, respectively, after *P.e.* infection compared to progenitors in infected control midguts (Figs 5E, 5G–5J′ and S5D; S2C Appendix). Further, the same depletion of *licorne* in progenitors decreased p38 activation throughout the midgut epithelium after infection compared to infected control midguts (Figs 5F–5J′ and S5E).

Together, these data indicate that Mkk3-p38 signalling in progenitors after *P.e.* infection stimulates ISC proliferation and activates p38 throughout the midgut epithelium.

### PGRP-LC/LE-Imd-Mkk3-p38 activity in progenitors activates p38 in the midgut epithelium after pathogenic bacterial infection

**Fig 6:**
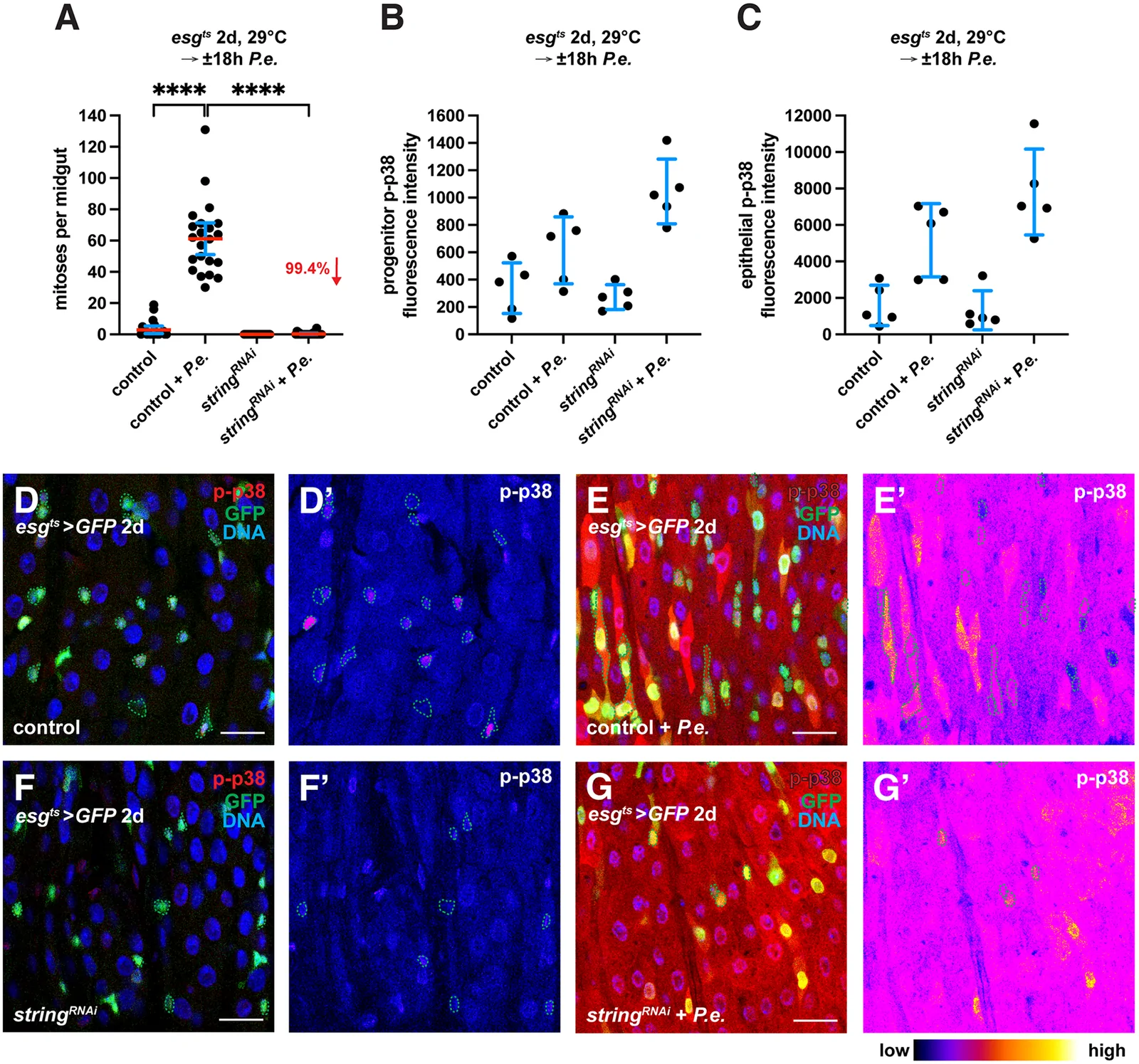
p38 activity in progenitors and not ISC proliferation induces p38 activation throughout the adult *Drosophila* midgut epithelium after pathogenic bacterial infection. ISC proliferation is not required for p38 activation in progenitors and throughout the midgut epithelium after *P.e.* infection **(A–G)**. The mean number of mitoses per midgut increased in infected control midguts (*n* = 22 from one of two experiments) compared to uninfected control midguts (*n* = 22 from one of two experiments) (Mann–Whitney; *p* < 0.0001). The mean number of mitoses decreased in infected midguts expressing *string*^*RNAi*^ in progenitors (*n* = 23 from one of two experiments) compared to infected control midguts (Mann–Whitney; *p* < 0.0001) **(A)**. Progenitor p-p38 fluorescence intensity increased in infected control midguts (*n* = 5 from one of two experiments) compared to progenitors in uninfected control midguts (*n* = 5 from one of two experiments). Progenitor p-p38 fluorescence intensity increased in infected midguts expressing *string*^*RNAi*^ with *esg*^*ts*^ (*n* = 5 from one of two experiments) compared to progenitors in infected control midguts **(B)**. The epithelial p-p38 fluorescence intensity per midgut from those analysed in B are shown in C. The epithelial p-p38 fluorescence intensity increased in *P.e.-*infected control midguts compared to uninfected control midguts. The epithelial p-p38 fluorescence intensity increased in infected midguts expressing *string*^*RNAi*^ in progenitors compared to infected control midguts **(C)**. p-p38 (D, E: red; D′, E′: Fire LUT) levels increased in progenitors (E: GFP; E-E′: green-dashed outline) and throughout the midgut epithelium after *P.e.* infection **(E–E′)** compared to uninfected control midguts **(D–D′)**. Blocking ISC proliferation by expressing *string*^*RNAi*^ with *esg*^*ts*^ did not inhibit the increase in p-p38 (G: red; G′: Fire LUT) in progenitors and throughout the midgut epithelium **(G–G′)** compared to *P.e*.-infected control midguts **(E–E′)**. Data in B–G′, acquired from R4a-b region. In A, mean (red line) and 95% confidence intervals (blue bars) are shown. In B–C, standard deviation (blue bars) is shown. DNA is in blue. Scale bars are 20 µm. Related supplementary data for this figure can be found in S6 Fig and S1 Data.

So far, we had shown that depleting p38a and p38b, Mkk3, Imd, PGRP-LC or PGRP-LE in midgut progenitors reduced p38 activation after *P.e.* infection not only within progenitors but also throughout the midgut epithelium. Furthermore, we had found that increasing ISC proliferation through Mkk3/Licorne overexpression led to p38 activation in nearby ECs. Thus, we asked whether it was increased PGRP-LC/LE-Imd-Mkk3-p38 signalling in progenitors that stimulated p38 activation throughout the midgut epithelium or rather increased ISC proliferation. To test this, we blocked ISC proliferation by depleting the *Drosophila* cdc25 phosphatase homolog, String, in progenitors with *esg*^*ts*^. This blocked ISC proliferation by 99.4% after *P.e.* infection compared to infected control midguts (Fig 6A). The decrease in ISC proliferation was not caused by reduced progenitor number due to *string* depletion with *esg*^*ts*^ and *P.e.* infection as the mean percent *esg*^*+*^ progenitors in these midguts was similar to uninfected and *P.e.*-infected control midguts (S6A Fig). Furthermore, blocking ISC proliferation did not decrease p38 activation, neither within progenitors (Fig 6B and 6D–6G′; S3A Appendix) nor throughout the epithelium (Fig 6C–6G′). These data suggest that pathogenic bacterial recognition via PGRP-LC/LE-Imd-Mkk3-p38 signalling in progenitors promotes p38 activation in the epithelial regenerative microenvironment.

### Recognition of pathogenic bacteria primarily by intestinal stem cells promotes adult midgut regeneration

**Fig 7:**
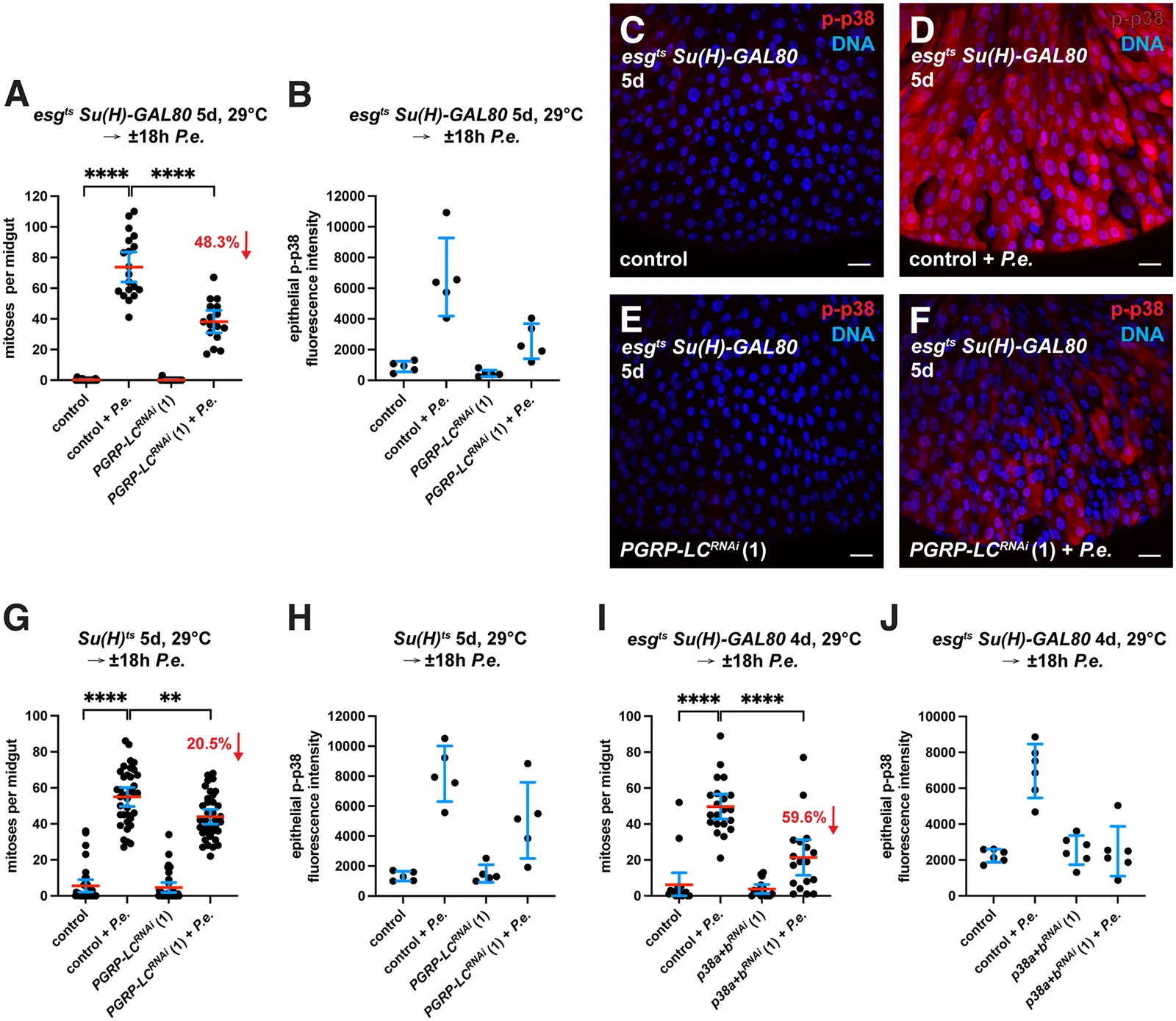
Recognition of pathogenic bacteria by ISCs via PGRP-LC stimulates p38 activation throughout the midgut epithelium and ISC proliferation. PGRP-LC signalling in ISCs stimulates their proliferation and p38 activation throughout the midgut epithelium **(A–F)**. The mean mitoses per midgut increased in infected control midguts (*n* = 19 from one of two experiments) compared to uninfected control midguts (*n* = 17 from one of two experiments) (Mann–Whitney; *p* < 0.0001). The mean mitoses per midgut decreased in infected midguts expressing *PGRP-LC*^*RNAi*^ (1) in ISCs (*n* = 16 from one of two experiments) compared to infected control midguts (Mann–Whitney; *p* < 0.0001) **(A)**. Epithelial p-p38 fluorescence intensity increased in infected control midguts (*n* = 5 from one of two experiments) compared to uninfected control midguts (*n* = 5 from one of two experiments). Epithelial p-p38 fluorescence intensity decreased in infected midguts expressing *PGRP-LC*^*RNAi*^ (1) in ISCs (*n* = 5 from one of two experiments) compared to infected control midguts **(B)**. p-p38 (red) increased in infected control midguts **(D)** compared to uninfected control midguts **(C)**; however, decreased in infected midguts expressing *PGRP-LC*^*RNAi*^ (1) in ISCs **(F)** compared to infected control midguts **(D)**. p-p38 levels were similar between uninfected control midguts **(C)** and uninfected midguts expressing *PGRP-LC*^*RNAi*^ (1) in ISCs **(E)**. PGRP-LC is partially required in enteroblasts (EBs) for ISC proliferation and p38 activation throughout the midgut epithelium **(G–H)**. The mean mitoses per midgut increased in infected midguts (*n* = 36 pooled from two experiments) compared to uninfected control midguts (*n* = 35 pooled from two experiments) (Mann–Whitney; *p* < 0.0001). The mean mitoses per midgut mildly decreased in infected midguts expressing *PGRP-LC*^*RNAi*^ (1) in EBs (*n* = 37 pooled from two experiments) compared to infected control midguts (Mann–Whitney; *p* = 0.0018) **(G)**. Epithelial p-p38 fluorescence intensity increased in infected control midguts (*n* = 5 from one of two experiments) compared to uninfected control midguts (*n* = 5 from one of two experiments). Epithelial p-p38 fluorescence intensity mildly decreased in infected midguts expressing *PGRP-LC*^*RNAi*^ (1) in EBs (*n* = 5 from one of two experiments) compared to infected control midguts **(H)**. p38 activity in ISCs stimulates their proliferation and p38 activation throughout the midgut epithelium **(I-J)**. The mean mitoses per midgut increased in infected control midguts (*n* = 21 pooled from two experiments) compared to uninfected control midguts (*n* = 18 pooled from two experiments) (Mann–Whitney; *p* < 0.0001). The mean mitoses per midgut decreased in infected midguts expressing *p38a+b*^*RNAi*^ (1) in ISCs (*n* = 18 pooled from two experiments) compared to infected control midguts (Mann–Whitney; *p* < 0.0001) **(I)**. Epithelial p-p38 fluorescence intensity increased in infected control midguts (*n* = 6 from one of two experiments) compared to uninfected control midguts (*n* = 6 from one of two experiments). Epithelial p-p38 fluorescence intensity decreased in infected midguts expressing *p38a+b*^*RNAi*^ (1) in ISCs (*n* = 6 from one of two experiments) compared to infected control midguts **(J)**. Data in B-F, H and J, acquired from R4a-b region. In A, G and I, mean (red line) and 95% confidence intervals (blue bars) are shown. In B, H and J, standard deviation (blue bars) is shown. DNA is in blue. Scale bars are 20 µm. Related supplementary data for this figure can be found in S6 Fig and S1 Data.

Because *esg*^*+*^ midgut progenitors include ISCs, EBs and EEps, it was not clear in which progenitor cell type(s) the recognition of pathogenic bacteria was important for midgut regeneration. Thus, we tested whether ISCs or EBs sensed pathogenic bacteria. To determine this, we depleted *PGRP-LC* from ISCs using an ISC-specific driver, *esg-GAL4 tubGAL80*^*ts*^ *Su(H)-GAL80* (*esg*^*ts*^ *Su(H)-GAL80*). This system allowed us to express *PGRP-LC*^*RNAi*^ in ISCs whilst blocking GAL4-mediated expression in *Suppressor of Hairless*-positive (*Su(H)*^*+*^) EBs by GAL80 expression. We found that *PGRP-LC* depletion from ISCs decreased ISC proliferation by 48.3% after *P.e.* infection compared to infected control midguts (Fig 7A). Furthermore, *PGRP-LC* depletion strongly reduced p38 activation throughout the midgut epithelium after *P.e.* infection (Fig 7B–7F). In contrast, we found a milder reduction in ISC proliferation (20.5%) and p38 activation in the midgut epithelium after *P.e.* infection when *PGRP-LC* was depleted from EBs using the EB-specific driver, *w; Su(H)Gbe-GAL4; tubGAL80*^*ts*^ (*Su(H)*^*ts*^) (Fig 7G and 7H). These data suggest that ISCs are the primary sensors of pathogenic bacteria amongst progenitors after infection.

We similarly asked whether p38a and p38b were required in ISCs or in EBs for ISC proliferation and p38 activation throughout the midgut epithelium. To determine this, we depleted *p38a* and *p38b* from ISCs using *esg*^*ts*^ *Su(H)-GAL80*. We found that *p38a* and *p38b* depletion in ISCs strongly reduced their proliferation after *P.e.* infection by 59.6% compared to infected control midguts (Fig 7I). Further, *p38a* and *p38b* depletion in ISCs strongly reduced p38 activation throughout the midgut epithelium after infection compared to infected control midguts (Fig 7J). In contrast, depleting *p38a* and *p38b* in EBs did not affect ISC proliferation after *P.e.* infection (S6B Fig).

Together, these data indicate that PGRP-LC-p38 signalling is essential in ISCs to detect pathogenic bacteria after infection and promote their proliferation.

**Fig 8:**
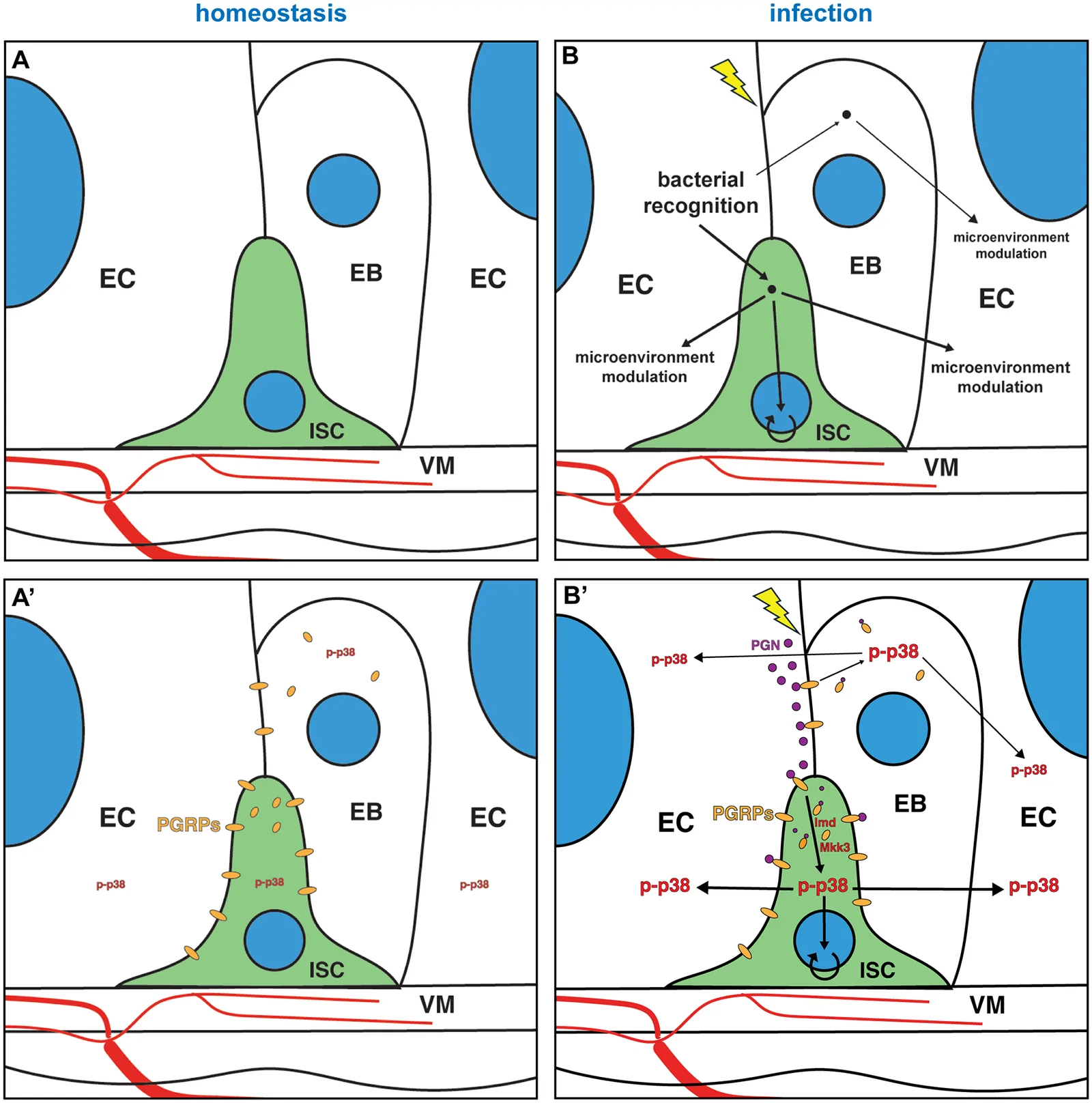
Recognition of pathogenic bacteria by intestinal stem cells via PGRP-Imd-Mkk3-p38 signalling promotes adult *Drosophila* midgut regeneration. Under homeostasis, pathogenic bacteria are not recognised by adult *Drosophila* midgut progenitors (intestinal stem cells: ISCs and enteroblasts: EBs) via PGRPs (PGRP-LC and PGRP-LE) and the levels of activated p38 remain low within progenitors and throughout the midgut epithelium **(A, A′)**. After infection, pathogenic bacteria are recognised by both ISCs and EBs, however, principally by ISCs. ISCs and EBs sense pathogenic bacteria partly through DAP-type peptidoglycan, thus activating Imd-Mkk3-p38 signalling. In ISCs, PGRP-LC/LE-Imd-Mkk3-p38 signalling promotes ISC proliferation; in both ISCs and EBs, PGRP-LC/LE-Imd-Mkk3-p38 signalling stimulates p38 activation throughout the epithelial regenerative microenvironment **(B, B′)**. ECs, enterocytes; VM, visceral muscle.

In summary, we have uncovered a mechanism by which pathogenic bacterial infection promotes ISC proliferation in the adult *Drosophila* midgut. We find that pathogenic bacteria are primarily recognised by ISCs through PGRP-LC and PGRP-LE, activating Imd-Mkk3-p38 signalling within ISCs. This p38 activity stimulates ISC proliferation but also stimulates p38 activation throughout the midgut epithelium—a component of the regenerative microenvironment (Fig 8).

## Discussion

Enteropathogenic bacterial infections damage the intestinal epithelium. When these bacteria breach the epithelial barrier, they are detected by intestinal epithelial cells and innate immune cells. This recognition triggers cytokine production and inflammation, which in turn promotes a regenerative response in the intestine. Bacterial pattern recognition factors like NOD2 are highly expressed in intestinal progenitors [31], but their role in intestinal regeneration after pathogenic bacterial infection is unclear.

In the adult *Drosophila* midgut, ECs serve as superb sensors of both bacteria [33,37,38] and midgut damage [29,39,48], positioning ECs as ideal mediators of the ISC regenerative response after pathogenic bacterial infection. Bacterial recognition by ECs via peptidoglycan results in both AMP and potentially ROS production to kill off invading pathogens [33,37,38,42]. Indeed, it has been proposed that ROS production via Duox leads to collateral epithelial damage and an ISC regenerative response. However, growing evidence indicates that this is unlikely the case [43,44]. Thus, it is not clear whether bacterial recognition can trigger a regenerative response in the adult *Drosophila* midgut. Pathogenic bacteria also directly damage epithelial cells through virulence factors. ECs utilise several damage-sensing mechanisms (p38, JNK, Hpo) to produce signalling cues that promote ISC proliferation after pathogenic bacterial infection [29,39,48]. Thus, it is unclear whether midgut epithelial cells couple pathogenic bacterial recognition and damage recognition to promote midgut regeneration. Here we have shown that ISCs in the adult *Drosophila* midgut recognise pathogenic bacteria after infection, translate this into their proliferation and influence damage-sensing mechanisms in the regenerative microenvironment.

We found that *PGRP-LC* and *PGRP-LE* mutants exhibited decreased ISC proliferation and p38 activation compared to control midguts after *P.e.* infection (Figs 1 and S1), indicating that both PGRPs are required for midgut regeneration, possibly via p38 signalling in ECs. Interestingly, ISC proliferation and midgut epithelial p38 activation in both *PGRP-LC* and *PGRP-LE* mutants reduced to a similar extent, suggesting that both proteins may function together in these processes rather than independently. Since pathogen recognition by PGRP-LC can activate p38 signalling in ECs [33] and p38 signalling in ECs promotes ISC proliferation [39], we tested whether PGRP-LC was required in ECs for ISC proliferation after *P.e.* infection. However, we found that this was not the case (S2A Fig). We thus hypothesised that PGRPs may be required in progenitors to promote infection-induced proliferation and p38 activation. Indeed, PGRP-LC is highly expressed in midgut progenitors [46]. Further, general ISC proliferation is known to increase with age in *Drosophila*, and both PGRP-LC and PGRP-LE are required for this increase [46]. We found that after *P.e.* infection, PGRP-LC and PGRP-LE were both required within progenitors for ISC proliferation, as depletion of either PGRP from progenitors strongly reduced ISC proliferation after infection compared to infected control midguts (Figs 2A, 2H, and S2B). Further, depleting *PGRP-LC* and *PGRP-LE* with *esg*^*ts*^ reduced p38 activation in progenitors after *P.e.* infection and, surprisingly, throughout the midgut epithelium (Figs 2B–2G′, 2I–2J, and S2E–S2H′). Together, these data suggest that recognition of pathogenic bacteria in progenitors through PGRP-LC and PGRP-LE stimulates p38 signalling throughout the midgut epithelium and promotes ISC proliferation. This could be by modulating signalling pathways known to promote ISC proliferation, such as Janus kinase-Signal transducer and activator of transcription (JAK-STAT), Epidermal growth factor receptor (EGFR), Wnt/Wingless (Wg) or FGF signalling after *P.e.* infection [14–17,19–21,25]. Furthermore, our data suggest that recognition of pathogenic bacteria by progenitors plays a significant role in the ISC response to *P.e.* infection and likely works synergistically with cues produced by the regenerative microenvironment [29] that forms after midgut damage, to fully stimulate ISC proliferation. Increased ISC proliferation after bacterial recognition may also serve as a strategy to repopulate the epithelium with uninfected cells.

Both Imd and Mkk3 are required for p38 activation in the midgut epithelium after exposure to peptidoglycan from *Ecc15* [33]. We similarly found that inhibiting Imd, Licorne (Mkk3), or both p38a and p38b in midgut progenitors decreased ISC proliferation and p38 activation in progenitors and throughout the midgut epithelium (Figs 3A–3G, 4, 5D–5J′, S4C–S4D, and S5C–S5E; S1C, S2A, and S2C Appendices). Previous work indicates that p38b, but not p38a and p38c, is required for midgut regeneration after *P.e.* infection [39]. We similarly found a strong role for p38b signalling in progenitors to stimulate p38 activation throughout the midgut epithelium (Fig 3B–3C and S1C Appendix). Together, our data indicate that bacterial recognition by progenitors via PGRP-Imd-Mkk3-p38 signalling promotes ISC proliferation and p38 activation throughout the midgut epithelium.

Our data suggests that PGRP-LC and PGRP-LE promote ISC proliferation and Mkk3-p38 signalling through Imd. However, inhibiting Imd in progenitors only partially decreased p38 activation in progenitors and throughout the midgut epithelium (Figs 4B–4G′ and S4D). Further, *imd*^*1*^ mutants did not consistently display a decrease in ISC proliferation (S4A–S4B Fig), raising the possibility of compensatory p38 activation in the midgut epithelium when Imd activity is suppressed systemically. Another possibility is that PGRP-LC and PGRP-LE in progenitors may regulate epithelial p38 activation and ISC proliferation through two separate pathways, Mkk3-p38 and Imd: Imd may partially regulate ISC proliferation through Mkk3-p38 signalling as well as through another mechanism after infection. In that case, this is unlikely to be through Relish activity since we did not find a defect in ISC proliferation in *Rel*^*E20*^ mutants after *P.e.* infection (S4F Fig).

The two PGRPs differ in their peptidoglycan specificities: PGRP-LC recognises extracellular polymeric and monomeric DAP-type peptidoglycan, whereas PGRP-LE detects monomeric DAP-type peptidoglycan intracellularly [34,36]. Our data suggest that progenitors sense both extracellular and intracellular peptidoglycan. Intracellular detection of DAP-type peptidoglycan could occur through the entry of monomeric peptidoglycan or pathogenic bacteria into midgut progenitors.

Since both PGRP-LC and PGRP-LE sense peptidoglycan, we asked whether peptidoglycan alone was able to stimulate ISC proliferation. We did not find this to be the case (Fig 3H). Instead, damage to the midgut epithelium was required for ISC proliferation via the peptidoglycan receptors (Figs 3K and S3C). One possibility is that damage compromises the intestinal barrier allowing peptidoglycan to reach basally localised stem cells. Another possibility is that another bacterial or host factor, possibly a damage-associated molecular pattern (DAMP) [49,50], is required together with peptidoglycan for PGRP-LC and PGRP-LE to promote ISC proliferation. This is consistent with our data showing that PGRP-LC is required for ISC proliferation after damage-causing pathogenic infection and heat-killed *P.e*., which we found to cause damage to the midgut epithelium (Figs 3K and S3C).

Interestingly, overexpressing Mkk3/Licorne to activate p38 in progenitors led to p38 activation not only within progenitors but also in neighbouring ECs (Figs 5B and 5C and S5A–S5B′). Indeed, we found a strong correlation between p-p38 levels in progenitors and their adjacent ECs in control midguts and in midguts with progenitors overexpressing Licorne (Fig 5C). Consistently, inhibition of p38a and p38b, Licorne, Imd, PGRP-LC or PGRP-LE in progenitors led to reduced p38 activation after infection not only within progenitors but also throughout the epithelium (Figs 2B–2G′, 2I–2J, 3B–3G, 4B–4G′, 5E–5J′, S2E–S2H′, S4D, and S5D–S5E; S1, S2A, and S2C Appendices). Further, we found that blocking ISC proliferation still stimulated p38 activation throughout the midgut epithelium after *P.e.* infection (Fig 6B–6G′ and S3A Appendix). These data suggest that PGRP-LC/LE-Imd-Mkk3-p38 signalling in progenitors, and not ISC proliferation, promotes p38 activation throughout the midgut epithelium after *P.e.* infection. Furthermore, it indicates that pathogenic bacterial sensing in midgut progenitors can modify the regenerative microenvironment to support regeneration. It is possible that p38 activity within progenitors stimulates p38 activation throughout the epithelium by a yet to be determined mechanism that influences Nox-Ask1 signalling in neighbouring ECs. Increased Nox-Ask1 signalling in ECs subsequently results in increased *upd3* expression that further supports ISC proliferation and midgut regeneration [39]. One possibility is that p38 signalling in ISCs promotes FGF signalling, which promotes ISC proliferation and tracheal remodelling [15], thus delivering more oxygen to the epithelium. This oxygen supply would fuel ROS production by Nox, thus activating the ROS-sensitive SAP3K, Ask1.

Previously, it was shown that damage sensing by ECs via Nox-Ask1 signalling activates p38 in ECs after *P.e.* infection [39]. Our data suggest that p38 is activated in midgut ECs by pathogenic bacterial recognition by progenitors through PGRP-LC and PGRP-LE. For the case of *P.e.* infection, it is possible that both damage sensing in ECs and pathogen recognition in progenitors are important to activate p38 in ECs to sufficiently high levels. Another study showed that p38a is activated in ECs after *Ecc15* infection by peptidoglycan recognition by PGRP-LC and Imd-Mekk1-Mkk3 signalling [33]. However, this study reduced Mkk3, Imd or PGRP-LC in the entire midgut epithelium, which could also reduce levels of Mkk3, Imd or PGRP-LC in progenitors. Consistent with this, we found that *Ecc15* is sensed by progenitors through PGRP-LC, which promotes p38 activation throughout the midgut epithelium and ISC proliferation (S2K–S2L Fig).

Finally, we have clarified which progenitor recognised pathogenic bacteria and stimulated p38 activation throughout the midgut epithelium. PGRP-LC, as well as both p38a and p38b, were required in ISCs for p38 activation throughout the midgut epithelium and for their proliferation after *P.e.* infection (Fig 7A–7F, 7I and 7J). In contrast, depleting *PGRP-LC* from EBs only mildly decreased ISC proliferation and p38 activation in the midgut epithelium (Fig 7G and 7H). Furthermore, depleting *p38a* and *p38b* from EBs had no effect on ISC proliferation (S6B Fig). Together, our findings suggest that ISCs are the principal sensors of pathogenic infection in the context of regeneration, responding by modifying the regenerative microenvironment and increasing their proliferation.

A similar mechanism may be in place in the mammalian intestine, with detection of bacterial peptidoglycan through NOD2 receptors influencing homeostatic epithelial growth. NOD2 sensing of the beneficial microbe *Lactiplantibacillus plantarum* has been shown to cause an increase in proliferative cell numbers in the small intestines of mice, alleviating stunted postnatal growth caused by undernutrition [32]. Furthermore, NOD2 is highly expressed in Lgr5^+^ ISCs in intestinal organoids, and treatment of intestinal organoids with muramyl dipeptide (MDP), a synthetic peptidoglycan fragment, increased organoid growth and cell survival in response to stress [31]. Our work provides an explanation for this result and indicates the potential for NOD2 in ISCs or intestinal progenitors to detect invading pathogenic bacteria and promote regenerative growth. Enteropathogenic *E. coli* (EPEC) is a major cause of diarrheal-related deaths worldwide, often due to dehydration and sepsis [51]. Thus, identifying targets for therapies that stimulate the regenerative response would be beneficial to restore water absorption and barrier function. In support of a role for NOD2 receptors in intestinal regeneration, mutations in the *NOD2* gene increase the risk of developing the inflammatory bowel disease, Crohn’s disease [52]. Intestines of Crohn’s patients are severely damaged and fail to regenerate [53]. Therefore, loss of bacterial detection by NOD2, particularly in ISCs, in inflammatory bowel diseases may contribute to reduced intestinal epithelial restitution in some Crohn’s disease patients. Treatments that enhance or restore bacterial recognition in intestinal stem cells may prove beneficial to these patients.

## Materials and Methods

### Fly stocks

The following fly stocks were used: *w*^*1118*^, *yw*, *w*; *esg-GAL4; tubGAL80*^*ts*^ *UAS-GFP* (*esg*^*ts*^), *esg-GAL4 UAS-2X EYFP; Su(H)Gbe-GAL80, tubGAL80*^*ts*^ (*esg*^*ts*^ *Su(H)-GAL80*), *w; Su(H)Gbe-GAL4; tubGAL80*^*ts*^ *UAS-GFP* (*Su(H)*^*ts*^) and *mex-GAL4; tubGAL80*^*ts*^ (*mex*^*ts*^). *UAS-imd*^*RNAi*^ (101834KK) [53], *UAS-licorne*^*RNAi*^ (1) (106822KK) [39], *w; UAS-p38a+b*^*RNAi*^ (1) (34238GD) [39], *w; UAS-p38a+b*^*RNAi*^ (2) (52277GD) [39], *w; UAS-PGRP-LC*^*RNAi*^ (1) (51968GD), *UAS-PGRP-LC*^*RNAi*^ (2) (101636KK) [46,54], *UAS-PGRP-LE*^*RNAi*^ (108199KK) [46] and KK control line (60100KK) from the Vienna *Drosophila* Stock Center (VDRC). *PGRP*^*Δ5*^, *Dipt2.2-lacZ; Drs-GFP, yw*, *w; UAS-imd*^*D30A*^*/MRKS*, *UAS-mCherry*^*RNAi*^, *UAS-licorne*^*RNAi*^ (2) (JF01433) and *UAS-string*^*RNAi*^ (HMS00146) from the Bloomington *Drosophila* Stock Center (BDSC). *yw; UAS-licorne* [39] from the Zürich Fly ORFeome project (FlyORF) and *UAS-p38b*^*antisense*^ [55] from the Kyoto Stock Center. *PGRP-LC*^*E12*^ and *PGRP-LE*^*112*^ are from Bruno Lemaitre (École Polytechnique Fédérale de Lausanne, EPFL) and *w; imd*^*1*^, isoDrosDel *Rel*^*E20*^ and isoDrosDel *w*^*1118*^ are from Mark Hanson (University of Exeter).

### *Drosophila melanogaster* housing and husbandry

*Drosophila melanogaster* were housed at either 18°C or 25°C (65% humidity) under a light-dark cycle and fed a *Drosophila* medium containing water, agar, glucose, dry yeast, wheat flour, 10% Nipagin and propionic acid.

### *Drosophila* genetics

Flies raised at 18°C were shifted to 29°C to induce GAL4-mediated *UAS*-transgene expression. Experiments were performed using 5–10 day old, adult, female *Drosophila melanogaster*. Typically, between 15 and 20 flies were used per experiment. Flies were first selected based on genotype, then randomly chosen for use in experiments. Genotypes for all experiments can be found in S1 and S2 Tables.

### Pathogenic bacterial culture and preparation for oral infection

*Pseudomonas entomophila* (*P.e.*) was grown by inoculating 3 mL of Luria Broth (LB) with 100 µg/mL rifampicin with a single colony of *P.e.* grown on a LB-rifampicin plate. The 3 mL culture was grown for 24 hours at 30°C at 130 RPM. 500 µL of this culture was diluted into 50 mL of fresh LB with rifampicin, which was then grown for 18 hours at 30°C at 130 RPM. The bacteria were then centrifuged at 3000*g* for 30 min. The bacterial pellet was then resuspended in 2 mL of 5% sucrose and diluted in 5% sucrose to CFU (colony forming unit) = 7.0 × 10^9^ bacteria/mL. Non-virulent *P.e. ΔgacA* were prepared similarly but resuspended to CFU = 4.1 × 10^10^ bacteria/mL. For feeding of heat-killed *P.e*., *P.e.* were prepared as above and then 1 mL of resuspended *P.e.* (CFU = 7.0 × 10^9^ bacteria/mL) was heated at 95°C for 30 min. *P.e.* viability after heat treatment was determined by streaking out lysates onto a LB-agar plate containing 100 µg/mL rifampicin. *P.e. ΔgacA* was provided by Bruno Lemaitre (École Polytechnique Fédérale de Lausanne, EPFL).

*Erwinia carotovora carotovora 15* (*Ecc15*) was grown by inoculating 3 mL of Luria Broth (LB) with 100 µg/mL rifampicin with a single colony of *Ecc15* grown on a LB-rifampicin plate. The 3 mL culture was grown for 24 hours at 30°C at 130 RPM. 2.5 mL of this culture was diluted into 250 mL of fresh LB with rifampicin, which was then grown for 18 hours at 30°C at 130 RPM. The bacteria were then centrifuged at 3000*g* for 30 min. The bacterial pellet was then resuspended in 1 mL of 5% sucrose and diluted in 5% sucrose to CFU = 3.8 × 10^11^ bacteria/mL.

### Oral infection of *Drosophila* with bacteria and feeding of heat-killed bacteria

Flies were fed food supplemented with 500 µL of 5% sucrose or *P.e.*, *P.e. ΔgacA* or *Ecc15* resuspended in 5% sucrose. This was done by adding 500 µL of each to the top layer of the food within a vial (25 mm wide) and mixing it into the food to a depth of approximately 3.7 mm. Heat-killed *P.e.* was fed similarly. Flies were continuously exposed to *P.e.*, heat-killed *P.e.*, *P.e. ΔgacA* or *Ecc15* for 18 hours or for 12 hours in Figs 2I–2J and S2E–S2H and S1B Appendix.

### Peptidoglycan feeding

Flies were exposed for 24 hours with a 45x26 mm rectangular Whatman filter paper soaked with either 250 µL of 5% sucrose or 5 mg/mL DAP-type peptidoglycan (PGN) suspended in 5% sucrose. PGN was an ultrapure preparation of DAP-type peptidoglycan isolated from *Escherichia coli K12* (InvivoGen). 5 mg of PGN was added to 1 mL LAL reagent water and sonicated six times using a Bioruptor UCD-200 (Diagenode) at 320 W for 30 seconds in an ice bath, followed by 30 seconds of rest. Sucrose was then added to make a solution of 5 mg/mL PGN in 5% sucrose.

### Histology

Adult *Drosophila* midguts were dissected in cold PBS (phosphate-buffered saline) and then fixed in 8% formaldehyde containing phosSTOP (Roche) for 1 hour. After fixation, midguts were washed at least three times in PBS, 0.1% Triton X-100 for 10–15 min. Midguts were then blocked for at least 30 min in blocking solution containing PBS, 0.1% Triton X-100, 1% bovine serum albumin (BSA) and 2% normal goat serum (NGS). After blocking, midguts were incubated overnight with primary antibodies in blocking solution at 4°C. The following primary antibodies were used: rabbit polyclonal anti-phospho Ser 10 histone 3 (Millipore; 06-570; 1:1000), mouse monoclonal anti-phospho Ser 10 histone 3 (Sigma-Aldrich; 3H10; 05-806; 1:1000), rabbit polyclonal anti-phosphorylated p38 (Thr180/Tyr182) (Cell Signaling; 9211; 1:200) and chicken polyclonal anti-ß-galactosidase (abcam; ab9361; 1:1000). Midguts were then washed three times as above in PBS containing 0.1% Triton X-100, stained in PBS, 0.3% Triton X-100, 0.1% BSA with Alexa Fluor-conjugated secondary antibodies (Life Technologies; 1:1000) and Hoechst 33258 (Life Technologies) and mounted in Vectashield (Vector Laboratories). Specificity of the antibody against phosphorylated p38 (Thr180/Tyr182) was previously tested in [39].

### Microscopy and image processing

Samples were analysed using a Leica DMI6000 epifluorescence microscope and a Leica SP5-II confocal laser scanning microscope. Images were processed using ImageJ (NIH) and Adobe Photoshop. Confocal images are presented as maximum intensity projections or as single slices. Each z-stack for each midgut within an experiment was acquired with the same laser intensity and gain. Regions R4a and R4b were imaged from randomly chosen midguts within a sample. For single slice images to which the Fire LUT (Look-Up Table) was applied, the range (minimum and maximum pixel) was set from 0 to 255 in ImageJ prior to applying the LUT.

The number of mitoses per midgut was determined by counting the number of phospho-Ser 10 histone 3-positive cells from whole adult female midguts. The mean number of mitoses per midgut and 95% confidence interval are presented for each genotype and treatment.

### Image quantitation

*Phosphorylated p38 in progenitors:* the Modular Image Analysis (MIA) plugin in Fiji (ImageJ) was used to trace around and individually track *esg* > GFP^+^ progenitors across a series of 10 consecutive slices from 0.25 or 0.5 μm z-stacks of the progenitor layer to generate a series of linked regions of interest (ROIs). These linked ROIs were applied onto corresponding slices of the p-p38 channel to measure the mean raw integrated density of each progenitor across the 10 slices. The mean raw integrated density of each progenitor was divided by the mean projected area of each progenitor to obtain the normalised p-p38 fluorescence intensity per progenitor. To exclude GFP^+^ early ECs from our analysis, we used a macro that we developed to identify cells with a nuclear size comparable to those of *esg* > GFP^+^ progenitors found in homeostatic midguts.

*Phosphorylated p38 in the midgut epithelium:* sum intensity projections of 10 slices from 0.5 µm z-stacks of the progenitor layer from regions R4a and R4b were generated for the p-p38 channel using ImageJ. The freehand or polygon selection tool was used to trace around the epithelium to measure the raw integrated density and epithelial area. The raw integrated density of p-p38 was then divided by the area of selected epithelium to obtain the normalised fluorescence intensity of p-p38 in the whole epithelium. These intensities were then further normalised by subtracting the background (raw integrated density/area) determined from a region of the image without tissue to obtain the final normalised p-p38 fluorescence intensity for the whole epithelium.

*Phosphorylatedp38 measurements between progenitors and adjacent ECs:* a slice from a 0.25 µm z-stack containing the majority of progenitors within the field was selected from the *esg* > GFP channel using ImageJ. A line (thickness = 5) was drawn between a progenitor and all adjacent ECs, starting within the progenitor. The middle point of the line was placed between these cells. The grey values along the line was determined from the corresponding slice from the p-p38 channel. The first half of the grey values were attributed to the progenitor, with the latter half to the adjacent EC. The grey values were then averaged. This process was repeated for all progenitors in the field. Average grey values for progenitors were graphed against those for adjacent ECs, and a Pearson’s correlation coefficient was determined using GraphPad Prism.

*% esg-positive cells:* maximum intensity projections of 10 slices from 0.25, 0.5 or 1 µm z-stacks of the progenitor layer from regions R4a and R4b were generated for the *esg* > GFP and DNA (Hoechst) channels in ImageJ. The total number of *esg* > GFP^*+*^ cells with small nuclei and the total number of nuclei were counted. The number of *esg* > GFP^+^ cells with small nuclei was then divided by the total number of nuclei to obtain % *esg-*positive cells.

### Quantitative RT-PCR

RNA was isolated from 24 midguts using the PicoPure RNA extraction kit and DNase treated during RNA isolation using the Qiagen RNase-free DNase Set. The RNA concentration was determined using a NanoDrop ND-1000 spectrophotometer. 200−250 ng of RNA was used for cDNA synthesis using the QuantiTect Reverse Transcription Kit or the Superscript IV First-Strand Synthesis System. qPCR was performed using the QuantiNova SYBR Green PCR Kit and Applied Biosystems QuantStudio 5 Real-Time qPCR System. Croquemort (Crq) was used as a reference. Each assay was performed in triplicate on two independent biological replicates. Fold changes were determined using the comparative Ct (ΔΔCt) method. A standard curve was performed to calculate primer efficiencies, which were between 90% and 110% for all primers. The following primers were used: PGRP-LE_F 5′-ACAGAAGCCCATGGACGAGC-3′; PGRP-LE_R 5′-ATCCCCGCGACTCAATGTGG-3′; PGRP-LC_F 5′-GCCGAAGCGGAGGATTATACGG-3′; PGRP-LC_R 5′-TGGTTAAGGTCGCGCAGGTG-3′; Crq_F 5′-CAGAGCTCTCCTCCGAATTG-3′ and Crq_R 5′-ATGCCGGTGATGAGAAAGAC-3′.

### Statistical analysis

Statistical analyses were conducted using GraphPad Prism 10. A two-sided Mann–Whitney test was applied to determine statistical significance. The significance level is denoted by an * for *p* ≤ 0.05, ** for *p* ≤ 0.01, *** *p* ≤ 0.001, and **** for *p* ≤ 0.0001, and ns, for not significant, *p*  >  0.05.

## Supporting information

S1 Fig PGRP-LC and PGRP-LE promote ISC proliferation and p38 activation throughout the midgut epithelium after pathogenic bacterial infection. PGRP-LE is required for epithelial p38 activation after *P.e.* infection **(A–D)**. Epithelial p-p38 (red) levels increased in infected control midguts **(B)** compared to uninfected control midguts **(A)**. Epithelial p-p38 levels decreased in infected *PGRP-LE*^*112*^ midguts **(D)** compared to infected control midguts **(B)**. Epithelial p-p38 levels were similar between uninfected control midguts **(A)** and uninfected *PGRP-LE*^*112*^ midguts **(C)**. PGRP-LC is required for ISC proliferation after *P.e.* infection **(E)**. The mean mitoses per midgut increased in infected control midguts (*n* = 34 pooled from two experiments) compared to uninfected control midguts (*n* = 36 pooled from two experiments) (Mann–Whitney; *p* < 0.0001). The mean mitoses per midgut decreased in infected *PGRP-LC*^*Δ5*^ midguts (*n* = 34 pooled from two experiments) compared to infected control midguts (Mann–Whitney; *p* = 0.0032) **(E)**. Data in A–D, acquired from R4a-b region. In E, mean (red line) and 95% confidence intervals (blue bars) are shown. DNA is in blue. Scale bars are 20 µm. Numerical data for this figure can be found in S1 Data.
(TIF)

S2 Fig PGRP-LC in progenitors but not in enterocytes stimulates ISC proliferation and p38 activation after pathogenic bacterial infection. PGRP-LC is required in progenitors and not enterocytes (ECs) for ISC proliferation after *P.e.* infection **(A–B)**. The mean mitoses per midgut increased in infected control midguts (*n* = 34 pooled from two experiments) compared to uninfected control midguts (*n* = 39 pooled from two experiments) (Mann–Whitney; *p* < 0.0001). The mean mitoses per midgut increased in infected midguts expressing *PGRP-LC*^*RNAi*^ (1) in ECs (*n* = 34 pooled from two experiments) compared to infected control midguts (Mann–Whitney; *p* = 0.0156) **(A)**. The mean mitoses per midgut increased in infected control midguts (*n* = 23 from one experiment) compared to uninfected control midguts (*n* = 24 from one experiment) (Mann–Whitney; *p* < 0.0001). The mean mitoses per midgut decreased in infected midguts expressing *PGRP-LC*^*RNAi*^ (2) in progenitors (*n* = 19 from one experiment) compared to infected control midguts (Mann–Whitney; *p* = 0.0002) **(B)**. Quantitative real-time PCR showed a decrease in both *PGRP-LC* and *PGRP-LE* transcripts in whole midguts after expressing *PGRP-LC*^*RNAi*^ (1) and *PGRP-LE*^*RNAi*^ in ECs (*n* = two experiments for both) **(C–D)**. PGRP-LE in progenitors promotes p38 activation in progenitors and throughout the midgut epithelium after *P.e.* infection **(E–H)**. p-p38 (E, F: red; E′, F′: Fire LUT) increased in progenitors (F: GFP; F–F′: green-dashed outline) and throughout the midgut epithelium after *P.e.* infection **(F–F′)** compared to uninfected control midguts **(E–E′)**. p-p38 (H: red; H′; Fire LUT) decreased in progenitors and throughout the midgut epithelium in midguts expressing *PGRP-LE*^*RNAi*^ with *esg*^*ts*^ **(H–H′)** compared to infected control midguts **(F–F′)**. Depleting *PGRP-LC* or *PGRP-LE* from progenitors does not affect their number after *P.e.* infection **(I–J)**. The percent *esg*^*+*^ cells was similar between infected and uninfected control midguts and between infected control midguts and infected midguts expressing *PGRP-LC*^*RNAi*^ (1) or *PGRP-LE*^*RNAi*^ (*n* = 6 from one of two experiments) **(I–J)**. PGRP-LC signalling in progenitors promotes ISC proliferation and epithelial p38 activation after *Ecc15* infection **(K–L)**. The mean mitoses per midgut increased in *Ecc15*-infected control midguts (*n* = 18 from one experiment) compared to uninfected control midguts (*n* = 21 from one experiment) (Mann–Whitney; *p* < 0.0001). The mean mitoses per midgut decreased in infected midguts expressing *PGRP-LC*^*RNAi*^ (1) in progenitors (*n* = 19 from one experiment) compared to infected control midguts (Mann–Whitney; *p* = 0.0128) **(K)**. Epithelial p-p38 fluorescence intensity increased in *Ecc15*-infected control midguts (*n* = 9 from one experiment) compared to uninfected control midguts (*n* = 9 from one experiment). Epithelial p-p38 fluorescence intensity decreased in infected midguts expressing *PGRP-LC*^*RNAi*^ (1) in progenitors (*n* = 9 from one experiment) compared to infected control midguts **(L)**. Data in E–J and L, acquired from R4a-b region. In A-B and K, mean (red line) and 95% confidence intervals (blue bars) are shown. In I–J and L, standard deviation (blue bars) is shown. DNA is in blue. Scale bars are 20 µm. Numerical data for this figure can be found in S1 Data.
(TIF)

S3 Fig DAP-type peptidoglycan and non-virulent *P.e.* elicit an innate immune response. Depleting p38a and p38b in progenitors does not affect their number after *P.e.* infection **(A–B)**. The percent *esg*^*+*^ cells was similar between infected and uninfected control midguts and between infected control midguts and infected midguts expressing *p38a+b*^*RNAi*^ (1) (*n* = 3–5 from one experiment) or *p38b*^*antisense*^ (*n* = 5 from one experiment) in progenitors **(A–B)**. PGRP-LE signalling in progenitors promotes ISC proliferation after exposure to heat-killed *P.e.* **(C)**. The mean mitoses per midgut increased in control midguts exposed to heat-killed (h-k) *P.e.* (*n* = 17 from one experiment) compared to control midguts (*n* = 19 from one experiment) (Mann–Whitney; *p* < 0.0001). The mean mitoses per midgut decreased in midguts expressing *PGRP-LE*^*RNAi*^ in progenitors exposed to heat-killed *P.e.* (*n* = 18 from one experiment) compared to control midguts exposed to heat-killed *P.e.* (Mann–Whitney; *p* = 0.0001) **(C)**. *Diptericin* expression increases in the midgut epithelium after exposure to DAP-type peptidoglycan or gram-negative pathogenic bacteria **(D–G)**. *Diptericin* expression reporter (*Dipt2.2-lacZ*) (D–G′; B-gal, red) was activated in midgut enterocytes exposed to DAP-type peptidoglycan, *P.e.* or *P.e. ΔgacA*. Data in A, B, acquired from R4a-b region. Data in D–G′, acquired from R5. In C, mean (red line) and 95% confidence intervals (blue bars) are shown. In A–B, standard deviation (blue bars) is shown. DNA is in blue. Scale bars are 20 µm. Numerical data for this figure can be found in S1 Data.
(TIF)

S4 Fig Imd signalling in progenitors stimulates ISC proliferation and p38 activation after pathogenic bacterial infection. ISC proliferation is not consistently affected in *imd* mutant midguts after *P.e.* infection **(A–B)**. The mean mitoses per midgut increased in infected control midguts (*n* = 50 pooled from three experiments) compared to uninfected control midguts (*n* = 47 pooled from three experiments) (Mann–Whitney; *p* < 0.0001). The mean mitoses per midgut in infected *imd*^*1*^ midguts (*n* = 49 pooled from three experiments) were similar to infected control midguts (Mann–Whitney; *p* = 0.1049) **(A)**. The mean mitoses per midgut for each genotype and treatment per experiment (*n* = 3) described in A **(B)**. Imd activity in progenitors promotes ISC proliferation and p38 activation throughout the midgut epithelium after *P.e.* infection **(C–D)**. The mean mitoses per midgut increased in infected control midguts (*n* = 19 from one of two experiments) compared to uninfected control midguts (*n* = 18 from one of two experiments) (Mann–Whitney; *p* < 0.0001). The mean mitoses per midgut decreased in infected midguts expressing *imd*^*RNAi*^ in progenitors (*n* = 19 from one of two experiments) compared to infected control midguts (Mann–Whitney; *p* < 0.0001) **(C)**. Epithelial p-p38 fluorescence intensity increased in infected control midguts (*n* = 6 from one experiment) compared to uninfected control midguts (*n* = 6 from one experiment). Epithelial p-p38 fluorescence intensity decreased in infected midguts expressing *imd*^*RNAi*^ in progenitors (*n* = 6 midguts from one experiment) compared to infected control midguts **(D)**. Inhibiting Imd signalling in progenitors does not decrease their number after *P.e.* infection **(E)**. The percent *esg*^*+*^ cells mildly increased between infected control midguts (*n* = 6 from one of two experiments) and uninfected control midguts (*n* = 6 from one of two experiments). The percent *esg*^*+*^ cells further increased in infected midguts expressing *imd*^*D30A*^ in progenitors (*n* = 6 from one of two experiments) compared to infected control midguts **(E)**. *Relish* is not required for ISC proliferation after *P.e.* infection **(F)**. The mean mitoses per midgut increased in infected control midguts (*n* = 17 from one experiment) compared to uninfected control midguts (*n* = 14 from one experiment) (Mann–Whitney; *p* < 0.0001). The mean mitoses per midgut in infected *Rel*^*E20*^ midguts (*n* = 17 from one experiment) was similar to infected control midguts (Mann–Whitney; *p* = 0.3890) **(F)**. Data in D–E, acquired from R4a-b region. In A, C and F, mean (red line) and 95% confidence intervals (blue bars) are shown. In B and D–E, standard deviation (blue bars) is shown. Numerical data for this figure can be found in S1 Data.
(TIF)

S5 Fig Mkk3/Licorne is necessary and sufficient to promote ISC proliferation and p38 activation after pathogenic bacterial infection. Licorne overexpression in progenitors increased p38 activation in progenitors and in adjacent ECs **(A–B)**. p-p38 (A, B: red; A′, B′: Fire LUT) increased in midgut progenitors (A, B: GFP; A′, B′, green-dashed outline) and in nearby epithelial cells after overexpressing *licorne* with *esg*^*ts*^ compared to control midguts **(A–B′)**. Licorne activity in progenitors promotes ISC proliferation after *P.e.* infection **(C)**. The mean number of mitoses per midgut increased in infected control midguts (*n* = 21 from one experiment) compared to uninfected control midguts (*n* = 19 from one experiment) (Mann–Whitney; *p* = 0.0169). The mean number of mitoses per midgut decreased in infected midguts expressing *licorne*^*RNAi*^ (2) in progenitors (*n* = 15 from one experiment) compared to infected control midguts (Mann–Whitney; *p* = 0.0465) **(C)**. Licorne is required in progenitors for p38 activation in progenitors and throughout the midgut epithelium after *P.e.* infection **(D–E)**. The progenitor p-p38 fluorescence intensity increased in infected control midguts (*n* = 5 from one experiment) compared to progenitors in uninfected control midguts (*n* = 5 from one experiment). The progenitor p-p38 fluorescence intensity decreased in infected midguts expressing *licorne*^*RNAi*^ (2) with *esg*^*ts*^ (*n* = 5 from one experiment) compared to progenitors in infected control midguts **(D)**. The epithelial p-p38 fluorescence intensity per midgut from those analysed in D are shown in E. The epithelial p-p38 fluorescence intensity increased in infected control midguts compared to uninfected control midguts. The epithelial p-p38 fluorescence intensity decreased in infected midguts expressing *licorne*^*RNAi*^ (2) in progenitors compared to infected control midguts **(E)**. Depleting *licorne* from progenitors does not affect their number after *P.e.* infection **(F-G)**. The percent *esg*^*+*^ cells was similar between infected and uninfected control midguts and between infected control midguts and infected midguts expressing *licorne*^*RNAi*^ (1) (*n* = 6 from one of two experiments) and *licorne*^*RNAi*^ (2) (*n* = 5 from one experiment) **(F–G)**. Data in A–B′ and D–G, acquired from R4a-b region. In C, mean (red line) and 95% confidence intervals (blue bars) are shown. In D–G, standard deviation (blue bars) is shown. DNA is in blue. Scale bars are 20 µm. Numerical data for this figure can be found in S1 Data.
(TIF)

S6 Fig p38 activity in enteroblasts does not promote ISC proliferation after pathogenic bacterial infection. Depleting *string* from progenitors does not affect their number after *P.e.* infection **(A)**. The percent *esg*^*+*^ cells was similar between infected and uninfected control midguts and between infected control midguts and infected midguts expressing *string*^*RNAi*^ in progenitors (*n* = 7 from one of two experiments) **(A)**. p38 activity is not required in enteroblasts (EBs) for ISC proliferation after *P.e.* infection **(B)**. The mean number of mitoses per midgut increased in infected control midguts (*n* = 36 pooled from two experiments) compared to uninfected control midguts (*n* = 35 pooled from two experiments) (Mann–Whitney; *p* < 0.0001). The mean number of mitoses per midgut in infected midguts expressing *p38a+b*^*RNAi*^ (1) in EBs (*n* = 35 pooled from two experiments) was similar to infected control midguts (Mann–Whitney; *p* = 0.3462) **(B)**. Data in A, acquired from R4a-b region. In B, mean (red line) and 95% confidence intervals (blue bars) are shown. In A, standard deviation (blue bars) is shown. Numerical data for this figure can be found in S1 Data.
(TIF)

S1 Appendix PGRP-LC and PGRP-LE promote p38 activation in progenitors after pathogenic bacterial infection. The distribution (histogram) of the normalised p-p38 fluorescence intensity per progenitor is shown for uninfected control midguts (*n* = 175), *P.e.-*infected control midguts (*n* = 142), uninfected midguts expressing *PGRP-LC*^*RNAi*^ (1) in progenitors (*n* = 233) and *P.e.-*infected midguts expressing *PGRP-LC*^*RNAi*^ (1) in progenitors (*n* = 153). The mean p-p38 level decreased by 105.0% in progenitors of infected midguts expressing *PGRP-LC*^*RNAi*^ (1) with *esg*^*ts*^ compared to progenitors in infected control midguts (Mann–Whitney; *p* < 0.0001). Basal p-p38 in progenitors of uninfected midguts expressing *PGRP-LC*^*RNAi*^ (1) with *esg*^*ts*^ was similar to progenitors in uninfected control midguts **(A)**. The distribution (histogram) of the normalised p-p38 fluorescence intensity per progenitor is shown for uninfected control midguts (*n* = 108), *P.e.-*infected control midguts (*n* = 202), uninfected midguts expressing *PGRP-LE*^*RNAi*^ in progenitors (*n* = 117) and *P.e.-*infected midguts expressing *PGRP-LE*^*RNAi*^ in progenitors (*n* = 178). The mean p-p38 level decreased by 74.2% in progenitors of infected midguts expressing *PGRP-LE*^*RNAi*^ with *esg*^*ts*^ compared to progenitors in infected control midguts (Mann–Whitney; *p* < 0.0001). Basal p-p38 in progenitors of uninfected midguts expressing *PGRP-LE*^*RNAi*^ with *esg*^*ts*^ was similar to progenitors in uninfected control midguts **(B)**. The distribution (histogram) of p-p38 fluorescence intensity per progenitor is shown for uninfected control midguts (*n* = 143), *P.e.*-infected control midguts (*n* = 143), uninfected midguts expressing *p38b*^*antisense*^ in progenitors (*n* = 136) and *P.e.*-infected midguts expressing *p38b*^*antisense*^ in progenitors (*n* = 142). The mean p-p38 level decreased by 87.8% in progenitors of infected midguts expressing *p38b*^*antisense*^ with *esg*^*ts*^ compared to progenitors in infected control midguts (Mann–Whitney; *p* < 0.0001). Basal p-p38 in progenitors of uninfected midguts expressing *p38b*^*antisense*^ with *esg*^*ts*^ was similar to progenitors in uninfected control midguts **(C)**. Data acquired from R4a-b. Histogram x-axis bin width is 50 (A–C). Numerical data for this figure can be found in S1 Data.
(TIF)

S2 Appendix Imd and Mkk3/Licorne promote p38 activation in progenitors after pathogenic bacterial infection. The distribution (histogram) of the normalised p-p38 fluorescence intensity per progenitor is shown for uninfected control midguts (*n* = 181), *P.e.-*infected control midguts (*n* = 187), uninfected midguts expressing *imd*^*D30A*^ in progenitors (*n* = 164) and *P.e.-*infected midguts expressing *imd*^*D30A*^ in progenitors (*n* = 173). The mean p-p38 level decreased by 43.9% in progenitors of infected midguts expressing *imd*^*D30A*^ with *esg*^*ts*^ compared to progenitors in infected control midguts (Mann–Whitney; *p* < 0.0001). Basal p-p38 in progenitors of uninfected midguts expressing *imd*^*D30A*^ with *esg*^*ts*^ was similar to progenitors in uninfected control midguts **(A)**. The distribution (histogram) of the normalised p-p38 fluorescence intensity per progenitor is shown for control midguts (*n* = 140) and midguts overexpressing *licorne* in progenitors (*n* = 184). The mean p-p38 level increased by 211.1% in progenitors of midguts overexpressing *licorne* with *esg*^*ts*^ compared to progenitors in control midguts (Mann–Whitney; *p* < 0.0001) **(B)**. The distribution (histogram) of the normalised p-p38 fluorescence intensity per progenitor is shown for uninfected control midguts (*n* = 174), *P.e.*-infected control midguts (*n* = 225), uninfected midguts expressing *licorne*^*RNAi*^ (1) in progenitors (*n* = 212) and *P.e.*-infected midguts expressing *licorne*^*RNAi*^ (1) in progenitors (*n* = 191). The mean p-p38 level decreased by 87.1% in progenitors of infected midguts expressing *licorne*^*RNAi*^ (1) with *esg*^*ts*^ compared to progenitors in infected control midguts (Mann–Whitney; *p* < 0.0001). Basal p-p38 in progenitors of uninfected midguts expressing *licorne*^*RNAi*^ (1) with *esg*^*ts*^ was similar to progenitors in uninfected control midguts **(C)**. Data acquired from R4a-b. Histogram x-axis bin width is 50 (A), 200 (B) or 100 (C). Numerical data for this figure can be found in S1 Data.
(TIF)

S3 Appendix Blocking ISC proliferation does not affect p38 activation in progenitors after pathogenic bacterial infection. The distribution (histogram) of the normalised p-p38 fluorescence intensity per progenitor is shown for uninfected control midguts (*n* = 121), *P.e.-*infected control midguts (*n* = 127), uninfected midguts expressing *string*^*RNAi*^ in progenitors (*n* = 143) and *P.e.-*infected midguts expressing *string*^*RNAi*^ in progenitors (*n* = 166). The mean p-p38 level increased by 205.2% in progenitors of infected midguts expressing *string*^*RNAi*^ with *esg*^*ts*^ compared to progenitors in infected control midguts (Mann–Whitney; *p* < 0.0001). Basal p-p38 in progenitors of uninfected midguts expressing *string*^*RNAi*^ with *esg*^*ts*^ was similar to progenitors in uninfected control midguts **(A)**. Data acquired from R4a-b. Histogram x-axis bin width is 100 (A). Numerical data for this figure can be found in S1 Data.
(TIF)

S1 Table Genotypes of flies used in Figs 1–7. (PDF)

S2 Table Genotypes of flies used in S1–S6 Figs and S1–S3 Appendices. (PDF)

S1 Data Numerical data for all quantifications in the manuscript are organised by figure panel per spreadsheet. For each genotype (± treatment), the number of measurements and mean are also provided.
(XLSX)

## Abbreviations

AMP: antimicrobial peptide
DAMP: damage-associated molecular pattern
EB: enteroblast
EC: enterocyte
EE: enteroendocrine cell
EEp: enteroendocrine precursor
EGF: epidermal growth factor
EGFR: Epidermal growth factor receptor
EPEC: Enteropathogenic *E. coli*
FGF: fibroblast growth factor
ISCs: intestinal stem cells
JAK-STAT: Janus kinase-Signal transducer and activator of transcription
LB: Luria Broth
MDP: muramyl dipeptide
NGS: normal goat serum
PGN: peptidoglycan
PGRPs: peptidoglycan recognition proteins
PTK7: protein tyrosine kinase 7
RIPK2: receptor-interacting serine/threonine protein kinase 2
RNAi: RNA interference
ROIs: regions of interest
ROS: reactive oxygen species
SAPK: stress-activated protein kinase
