# Supplementary material for: Recognition of pathogenic bacteria by intestinal stem cells promotes adult Drosophila midgut regeneration
Source: PLoS Biol. 2026 Aug 25;24(8):e3003951. doi: 10.1371/journal.pbio.3003951 (PMC13541249; doi:10.1371/journal.pbio.3003951)
Supplement: Supplementary file 1 [file pbio.3003951.s010.pdf]

Figure		Genotype
Figure 1	A-B	yw/w^{1118}
	C-D	yw, Dipt-lacZ, Drs-GFP/w; +/+; PGRP-LC^{E12}/PGRP-LC$^{\Delta5}$
	E,G	yw/w^{1118}<br>OR<br>yw, Dipt-lacZ, Drs-GFP/w; +/+; PGRP-LC^{E12}/PGRP-LC$^{\Delta5}$
	F,H	yw/yw<br>OR<br>yw, PGRP-LE^{112}/yw, PGRP-LE^{112}; +/+; +/+
Figure 2	A-C	w/w^{1118}; esg-GAL4/+; tubGAL80^{ts}, UAS-GFP/+<br>OR<br>w/UAS-PGRP-LC^{RNAi} (1), w; esg-GAL4/+; tubGAL80^{ts}, UAS-GFP/+
	D-D', E-E'	w/w^{1118}; esg-GAL4/+; tubGAL80^{ts}, UAS-GFP/+
	F-F', G-G'	w/UAS-PGRP-LC^{RNAi} (1), w; esg-GAL4/+; tubGAL80^{ts}, UAS-GFP/+
	H-J	w/w; esg-GAL4/P{attP,y[+],w[3`]}; tubGAL80^{ts}, UAS-GFP/+<br>OR<br>w/w; esg-GAL4/UAS-PGRP-LE^{RNAi}; tubGAL80^{ts}, UAS-GFP/+
Figure 3	A	w/w^{1118}; esg-GAL4/+; tubGAL80^{ts}, UAS-GFP/+<br>OR<br>w/w; esg-GAL4/UAS-p38a+b^{RNAi} (1); tubGAL80^{ts}, UAS-GFP/+
	B	w/w^{1118}; esg-GAL4/+; tubGAL80^{ts}, UAS-GFP/+<br>OR<br>UAS-p38b^{antisense}, w/w, esg-GAL4/+; tubGAL80^{ts}, UAS-GFP/+
	C	w/w^{1118}; esg-GAL4/+; tubGAL80^{ts}, UAS-GFP/+<br>OR<br>w/w; esg-GAL4/+; tubGAL80^{ts}, UAS-GFP/UAS-p38a+b^{RNAi} (2)<br>OR<br>UAS-p38b^{antisense}, w/w, esg-GAL4/+; tubGAL80^{ts}, UAS-GFP/+
	D-E	w/w^{1118}; esg-GAL4/+; tubGAL80^{ts}, UAS-GFP/+
	F-G	w/w; esg-GAL4/+; tubGAL80^{ts}, UAS-GFP/UAS-p38a+b^{RNAi} (2)
	H	Dipt2.2-lacZ, Drs-GFP, yw/Dipt2.2-lacZ, Drs-GFP, yw
	I-J	w^{1118}/w^{1118}
	K	w/w; esg-GAL4/P{attP,y[+],w[3`]}; tubGAL80^{ts}, UAS-GFP/+<br>OR<br>w/w; esg-GAL4/UAS-PGRP-LC^{RNAi} (2); tubGAL80^{ts}, UAS-GFP/+
Figure 4	A-C	w/w^{1118}; esg-GAL4/+; tubGAL80^{ts}, UAS-GFP/+<br>OR<br>w/w; esg-GAL4/+; tubGAL80^{ts}, UAS-GFP/UAS-imd^{D30A}
	D-D', E-E'	w/w^{1118}; esg-GAL4/+; tubGAL80^{ts}, UAS-GFP/+
	F-F', G-G'	w/w; esg-GAL4/+; tubGAL80^{ts}, UAS-GFP/UAS-imd^{D30A}
Figure 5	A-C	w/w^{1118}; esg-GAL4/+; tubGAL80^{ts}, UAS-GFP/+<br>OR<br>w/yw; esg-GAL4/+; tubGAL80^{ts}, UAS-GFP/UAS-licorne
	D-F	w/w; esg-GAL4/P{attP,y[+],w[3`]}; tubGAL80^{ts}, UAS-GFP/+<br>OR<br>w/w; esg-GAL4/UAS-licorne^{RNAi} (1); tubGAL80^{ts}, UAS-GFP/+
	G-G', H-H'	w/w; esg-GAL4/P{attP,y[+],w[3`]}; tubGAL80^{ts}, UAS-GFP/+
	I-I', J-J'	w/w; esg-GAL4/UAS-licorne^{RNAi} (1); tubGAL80^{ts}, UAS-GFP/+
Figure 6	A-C	w/yv; esg-GAL4/+; tubGAL80^{ts}, UAS-GFP/UAS-mCherry^{RNAi}<br>OR<br>w/yv; esg-GAL4/+; tubGAL80^{ts}, UAS-GFP/UAS-string^{RNAi}
	D-D', E-E'	w/yv; esg-GAL4/+; tubGAL80^{ts}, UAS-GFP/UAS-mCherry^{RNAi}
	F-F', G-G'	w/yv; esg-GAL4/+; tubGAL80^{ts}, UAS-GFP/UAS-string^{RNAi}
Figure 7	A-B	w/w^{1118}; esg-GAL4 UAS-2X EYFP/+; Su(H)Gbe-GAL80, tubGAL80^{ts}/+<br>OR

		UAS-PGRP-LC^{RNAi} (1)*, w/w; esg-GAL4 UAS-2X EYFP/+; Su(H)Gbe-GAL80, tubGAL80^{ts}/+*
	C-D	*w/w^{1118}; esg-GAL4 UAS-2X EYFP/+; Su(H)Gbe-GAL80, tubGAL80^{ts}/+*
	E-F	*w/UAS-PGRP-LC^{RNAi}* (1)*, w; esg-GAL4 UAS-2X EYFP/+; Su(H)Gbe-GAL80, tubGAL80^{ts}/+*
	G-H	*w/w^{1118}; Su(H)Gbe-GAL4/+; tubGAL80^{ts} UAS-GFP/+*<br>OR<br>*w/UAS-PGRP-LC^{RNAi}* (1)*, w; Su(H)Gbe-GAL4/+; tubGAL80^{ts} UAS-GFP/+*
	I-J	*w/w^{1118}; esg-GAL4 UAS-2X EYFP/+; Su(H)Gbe-GAL80, tubGAL80^{ts}/+*<br>OR<br>*w/w; esg-GAL4 UAS-2X EYFP/UAS-p38a+b^{RNAi}* (1)*; Su(H)Gbe-GAL80, tubGAL80^{ts}/+*
