# Supplementary material for: Recognition of pathogenic bacteria by intestinal stem cells promotes adult Drosophila midgut regeneration
Source: PLoS Biol. 2026 Aug 25;24(8):e3003951. doi: 10.1371/journal.pbio.3003951 (PMC13541249; doi:10.1371/journal.pbio.3003951)
Supplement: Supplementary file 2 [file pbio.3003951.s011.pdf]

Figure		Genotype
S1 Figure	A-B	yw/yw
	C-D	yw, PGRP-LE^{112}/yw, PGRP-LE^{112}; +/+; +/+
	E	w^{1118}/w^{1118}<br>OR<br>w/w; +/+; PGRP-LC^{Δ5}/PGRP-LC^{Δ5}
S2 Figure	A,C	w/w^{1118}; mex-GAL4/+; tubGAL80^{ts}/+<br>OR<br>w/UAS-PGRP-LC^{RNAi} (1), w; mex-GAL4/+; tubGAL80^{ts}/+
	B	w/w; esg-GAL4/P{attP,y[+],w[3`]}; tubGAL80^{ts}, UAS-GFP/+<br>OR<br>w/w; esg-GAL4/UAS-PGRP-LC^{RNAi} (2); tubGAL80^{ts}, UAS-GFP/+
	D	w/w^{1118}; mex-GAL4/+; tubGAL80^{ts}/+<br>OR<br>w/w; mex-GAL4/UAS-PGRP-LE^{RNAi}; tubGAL80^{ts}/+
	E-E', F-F'	w/w; esg-GAL4/P{attP,y[+],w[3`]}; tubGAL80^{ts}, UAS-GFP/+
	G-G', H-H'	w/w; esg-GAL4/UAS-PGRP-LE^{RNAi}; tubGAL80^{ts}, UAS-GFP/+
	I,K,L	w/w^{1118}; esg-GAL4/+; tubGAL80^{ts}, UAS-GFP/+<br>OR<br>w/UAS-PGRP-LC^{RNAi} (1), w; esg-GAL4/+; tubGAL80^{ts}, UAS-GFP/+
	J	w/w; esg-GAL4/P{attP,y[+],w[3`]}; tubGAL80^{ts}, UAS-GFP/+<br>OR<br>w/w; esg-GAL4/UAS-PGRP-LE^{RNAi}; tubGAL80^{ts}, UAS-GFP/+
S3 Figure	A	w/w^{1118}; esg-GAL4/+; tubGAL80^{ts}, UAS-GFP/+<br>OR<br>w/w; esg-GAL4/UAS-p38a+b^{RNAi} (1); tubGAL80^{ts}, UAS-GFP/+
	B	w/w^{1118}; esg-GAL4/+; tubGAL80^{ts}, UAS-GFP/+<br>OR<br>w/UAS-p38b^{antisense}, w; esg-GAL4/+; tubGAL80^{ts}, UAS-GFP/+
	C	w/w; esg-GAL4/P{attP,y[+],w[3`]}; tubGAL80^{ts}, UAS-GFP/+<br>OR<br>w/w; esg-GAL4/UAS-PGRP-LE^{RNAi}; tubGAL80^{ts}, UAS-GFP/+
	D-G'	Dipt2.2-lacZ, Drs-GFP, yw/Dipt2.2-lacZ, Drs-GFP, yw
S4 Figure	A,B	w^{1118}/w^{1118}<br>OR<br>w/w; imd^{1}/imd^{1}; +/+
	C-D	w/w; esg-GAL4/P{attP,y[+],w[3`]}; tubGAL80^{ts}, UAS-GFP/+<br>OR<br>w/w; esg-GAL4/UAS-imd^{RNAi}; tubGAL80^{ts}, UAS-GFP/+
	E	w/w^{1118}; esg-GAL4/+; tubGAL80^{ts}, UAS-GFP/+<br>OR<br>w/w; esg-GAL4/+; tubGAL80^{ts}, UAS-GFP/UAS-imd^{D30A}
	F	w$^{1118\ DrosDel}$/w$^{1118\ DrosDel}$<br>w$^{1118\ DrosDel}$/w$^{1118\ DrosDel}$; +/+; Rel^{E20}/Rel^{E20}
S5 Figure	A-A'	w/w^{1118}; esg-GAL4/+; tubGAL80^{ts}, UAS-GFP/+
	B-B'	w/yw; esg-GAL4/+; tubGAL80^{ts}, UAS-GFP/UAS-licorne
	C-E, G	w/yv; esg-GAL4/+; tubGAL80^{ts}, UAS-GFP/UAS-mCherry^{RNAi}<br>OR<br>w/yv; esg-GAL4/+; tubGAL80^{ts}, UAS-GFP/UAS-licorne^{RNAi} (2)
	F	w/w; esg-GAL4/P{attP,y[+],w[3`]}; tubGAL80^{ts}, UAS-GFP/+<br>OR<br>w/w; esg-GAL4/UAS-licorne^{RNAi} (1); tubGAL80^{ts}, UAS-GFP/+
S6 Figure	A	w/yv; esg-GAL4/+; tubGAL80^{ts}, UAS-GFP/UAS-mCherry^{RNAi}<br>OR<br>w/yv; esg-GAL4/+; tubGAL80^{ts}, UAS-GFP/UAS-string^{RNAi}
	B	w/w^{1118}; Su(H)Gbe-GAL4/+; tubGAL80^{ts} UAS-GFP/+

		OR<br>*w/w; Su(H)Gbe-GAL4/UAS-p38a+b*^{RNAi} (1)*; tubGAL80*^{ts} *UAS-GFP/+*
S1 Appendix	A	*w/w*^{1118}*; esg-GAL4/+; tubGAL80*^{ts}*, UAS-GFP/+*<br>OR<br>*w/UAS-PGRP-LC*^{RNAi} (1)*, w; esg-GAL4/+; tubGAL80*^{ts}*, UAS-GFP/+*
	B	*w/w; esg-GAL4/P{attP,y[+],w[3`]}; tubGAL80*^{ts}*, UAS-GFP/+*<br>OR<br>*w/w; esg-GAL4/UAS-PGRP-LE*^{RNAi}*; tubGAL80*^{ts}*, UAS-GFP/+*
	C	*w/w*^{1118}*; esg-GAL4/+; tubGAL80*^{ts}*, UAS-GFP/+*<br>OR<br>*w/UAS-p38b*^{antisense}*, w; esg-GAL4/+; tubGAL80*^{ts}*, UAS-GFP/+*
S2 Appendix	A	*w/w*^{1118}*; esg-GAL4/+; tubGAL80*^{ts}*, UAS-GFP/+*<br>OR<br>*w/w; esg-GAL4/+; tubGAL80*^{ts}*, UAS-GFP/UAS-imd*^{D30A}
	B	*w/w*^{1118}*; esg-GAL4/+; tubGAL80*^{ts}*, UAS-GFP/+*<br>OR<br>*w/yw; esg-GAL4/+; tubGAL80*^{ts}*, UAS-GFP/UAS-licorne*
	C	*w/w; esg-GAL4/P{attP,y[+],w[3`]}; tubGAL80*^{ts}*, UAS-GFP/+*<br>OR<br>*w/w; esg-GAL4/UAS-licorne*^{RNAi} (1)*; tubGAL80*^{ts}*, UAS-GFP/+*
S3 Appendix	A	*w/yv; esg-GAL4/+; tubGAL80*^{ts}*, UAS-GFP/UAS-mCherry*^{RNAi}<br>OR<br>*w/yv; esg-GAL4/+; tubGAL80*^{ts}*, UAS-GFP/UAS-string*^{RNAi}
