# Supplementary material for: Recognition of pathogenic bacteria by intestinal stem cells promotes adult Drosophila midgut regeneration
Source: PLoS Biol. 2026 Aug 25;24(8):e3003951. doi: 10.1371/journal.pbio.3003951 (PMC13541249; doi:10.1371/journal.pbio.3003951)
Supplement: Supplementary file 4 [file pbio.3003951.s014.pdf]

Revision Plan

Review COMMONS

Manuscript number: RC-2025-03047
Corresponding author(s): Parthive H. Patel

[The "revision plan" should delineate the revisions that authors intend to carry out in response to the points raised by the referees. It also provides the authors with the opportunity to explain their view of the paper and of the referee reports.

The document is important for the editors of affiliate journals when they make a first decision on the transferred manuscript. It will also be useful to readers of the reprint and help them to obtain a balanced view of the paper.

If you wish to submit a full revision, please use our "[Full Revision]" template. ***It is important to use the appropriate template to clearly inform the editors of your intentions.****]*

1. General Statements [optional]

This section is optional. Insert here any general statements you wish to make about the goal of the study or about the reviews.

We thank the reviewers for their careful and constructive assessment of our work, which will help us further improve our manuscript. Below we have outlined our plans for the revision.

Our work shows that adult *Drosophila* intestinal progenitors recognise pathogenic bacteria, induce intestinal stem cell (ISC) proliferation, and non-cell autonomously shape the regenerative niche. We find that recognition of pathogenic bacteria by progenitors occurs via two peptidoglycan recognition proteins, PGRP-LC and PGRP-LE. Further, we show that PGRP-mediated recognition in progenitors stimulates MKK3-p38 signalling within progenitors and triggers ISC proliferation. In addition, we found that PGRP-LC/LE-MKK3-p38 signalling in progenitors induces trans-epithelial p38 activation, indicating that bacterial sensing by progenitors can shape the regenerative niche. To date, this is the first description of the requirement of PGRPs and MKK3-p38 signalling in progenitors for midgut regeneration.

Various mechanisms by which differentiated cell types sense pathogenic bacteria and signal to ISCs to proliferate for regeneration have been reported. Peptidoglycan sensing by midgut enterocytes (ECs) is vital for an innate immunity response to pathogenic bacteria (anti-microbial peptide and reactive oxygen species (ROS) production by Dual oxidase (Duox)) (Ha et al., 2005; Bosco-Drayon et al., 2012; Neyen et al., 2012). Interestingly, ROS production has also been reported to indirectly elicit a regenerative response, possibly due to collateral damage (Buchon et al., 2009). Peptidoglycan sensing by ECs increases ROS production by inducing Dual oxidase (Duox) expression (Ha et al., 2009). Depleting *Duox* expression throughout the midgut blocked ISC proliferation after infection (Buchon et al., 2009), suggesting that Duox-

derived ROS trigger a regenerative response. However, this has been recently challenged by Liu et al., 2024 as depleting *Duox* specifically in each midgut epithelial cell type did not affect ISC proliferation after infection. Thus, it remains unknown whether midgut cell types, particularly ISCs, can directly recognise peptidoglycan and contribute to regeneration. Moreover, bacterial-induced damage sensing by ECs and other midgut cell types also stimulates ISC proliferation via secreted cues (see Zhang and Edgar, 2022).

Our study, however, changes the view that only differentiated cell types sense pathogenic bacteria by showing that intestinal progenitors directly sense *Pseudomonas entomophila* during infection, which triggers ISC proliferation. Our recent preliminary data strengthen this conclusion by showing that pathogenic bacterial sensing occurs specifically in ISCs, which triggers trans-epithelial p38 signalling throughout the midgut epithelium (see Reviewer 2, point 6). Furthermore, we have preliminary data suggesting that Imd signalling is involved in p38 activation within progenitors (see Reviewer 2, point 3). Hence, we are convinced that we will be able to address the reviewer points with further experimental data, resulting in a stronger study with deeper mechanistic insight.

2. Description of the planned revisions

Insert here a point-by-point reply that explains what revisions, additional experimentations and analyses are *planned* *to address the points raised by the referees.*

Reviewer #1

Evidence, reproducibility and clarity

Summary:
This research article demonstrates that intestinal stem cells (ISCs) in the adult female Drosophila melanogaster midgut can detect peptidoglycans from the Gram-negative bacterium Pseudomonas entomophila through the PGRP-LC/LE receptors. This detection triggers MKK3-p38 signaling in both intestinal progenitors and enterocytes, promoting ISC proliferation.
The authors' conclusions are well supported by the presented experimental data, which are clearly described and thoughtfully discussed in the context of existing literature. They also suggest future research directions and consider the evolutionary implications of their findings. Notably, it remains unclear how peptidoglycan sensing in progenitors leads to p38 activation in enterocytes. This signaling mechanism may be specific to certain bacteria, such as P. entomophila, and could require additional cues not present in other species.

We thank the reviewer for their positive evaluation of our work and for noting that our “conclusions are well supported by the presented experimental data”.

Major points (optional):
To strengthen this point, the authors could optionally extend their study by including

experiments with Erwinia carotovora carotovora 15 (Ecc15) and Acetobacter species-at least assessing mitotic activity and p38 activation. Further functional analyses might also clarify how bacterial signals trigger ISC activation and how ISCs coordinate with enterocytes to induce p38 signaling and downstream responses. Additionally, investigating enterocyte turnover and their replacement dynamics by proliferating progenitors could enrich the mechanistic insight. Nonetheless, the current data are sufficient to support the central claims of the manuscript. The methodology and data analysis are sound and appropriate for substantiating the reported phenomena.

We thank the reviewer for these suggestions. We believe that the induction of PGRP-MKK3-p38 signalling is not specific to certain bacteria (*P.e.*), since previous studies have shown that *Ecc15* can activate p38 in the adult *Drosophila* midgut epithelium (Ha et al., 2009, Houtz et al., 2017) and induce ISC proliferation (Buchon et al., 2009). We will add this information to our Discussion. Nevertheless, we would be interested in extending this analysis further to other gram-negative species such as Acetobacter in the future. We agree with the reviewer that it remains unclear how peptidoglycan sensing in progenitors leads to p38 activation in enterocytes. We will propose some potential mechanisms in our Discussion (see Reviewer 3, point 5), and we hope to investigate these further in the future. Moreover, to further clarify how bacterial signals trigger ISC activation, we will investigate a role for *Imd* and Relish (see Reviewer 2, point 3). Our preliminary data suggest that *Imd* activity in progenitors can induce ISC proliferation and trans-epithelial p38 activation. Lastly, enterocyte turnover and replacement after *P.e.* infection have been reported in Jiang et al, 2009; *Cell*. However, if we were able to activate p38 and ISC proliferation with peptidoglycan feeding (see Review 2, point 2 and Reviewer 3, point 2), it would be interesting to examine whether peptidoglycan alone could influence epithelial turnover dynamics.

Minor points (to be addressed):
1. Some measured parameters seem to be analysed in midgut regions, while the number of mitoses were measured in the whole midgut. In cases when particular gut regions were selected, figure legend should be adjusted accordingly, with explanation which midgut regions were analysed, and the method section should describe which measures the authors undertook to avoid biases due to differences between physiological regions of the midgut. In cases when the whole midgut was analysed, the total number of midgut cells, and the total number of GFP-positive cells for each midgut ought to be indicated in an additional supplementary table.

We thank the reviewer for this suggestion. Activated p38 (p-p38) is frequently observed in unchallenged midguts in the anterior midgut (regions R1 and anterior R2) and occasionally in and around the mid-midgut (R3). In contrast, p38 activity is generally absent in the posterior midgut (R4–R5), where only occasional low levels of p38 activation are detected (Patel et al., 2019). Thus, we analysed regions R4a and R4b in this study. Regions R4a and R4b were imaged from randomly chosen midguts within a sample. We have now added this information to our figure legends and the Methods.

The number of mitoses were, however, quantitated throughout the entire midgut. It is not clear, however, why the total number of midgut cells and the total number of GFP-positive cells are suggested to be indicated in a supplementary table. This is not a common practice within our field; thus, we have not collected this information from our experimental samples. See these papers (Jiang *et al.*, 2009; *Cell* (Edgar lab), Buchon *et al.*, 2009; Genes and Dev (Lemaitre lab); Hudry *et al.*, 2016; *Nature* (Miguel-Aliaga lab); Patel *et al*., 2019; *Nat Commum* (Edgar lab); Perochon *et al*., 2021; *Nat Cell Biol* (Cordero lab)*;* Petsakou *et al*., 2023; *Nature* (Perrimon lab)). If the reviewer is concerned whether RNAi expression and *P.e.* infection leads to decreased progenitor numbers, we have provided this analysis within a portion of R4a and R4b in Supplementary Figures 1A-B, 2B-C and 3B-D. We found in all of our experiments that RNAi expression in progenitors and *P.e.* infection did not affect midgut progenitor number.

2. Statistical analyses should be described more cautiously when small sample volumes are presented (especially for n<10). In cases of n = 5 or less, representation of mean and 95%CI on graphs is meaningless and statistically wrong. Corrections for multiple comparisons should be taken into consideration when such analysis was conducted (Fig 1 G, for example)

We thank the reviewer for pointing this out. We have consulted a data scientist at the University of Bristol, who suggested we use descriptive statistics rather than inferential statistics to describe our data. Thus, we will only include the standard deviation in graphs displaying data per midgut. We will edit our graphs and figure legends in our revised manuscript. Moreover, we haven't performed multiple comparisons between samples within an experiment. We report here only comparisons between two independent groups.

3. In graphs where the number of progenitors with different fluorescence intensity is represented (e.g. Fig 1 C), the range of intervals for each fluorescent group should be noted in the legend. Otherwise it is not clear why there are white spots between histogram columns (supposedly, continuous range of fluorescence intensity was initially split into intervals with no gaps).

We thank the reviewer for this suggestion. We will include these ranges in our revised figure legends. For example, in Fig 1C, the first bar represents intensity values binned from 0-500 and each subsequent bar, every 1000.

4. How do the authors describe higher activation of p38 in PGRP-LE knockdown in comparison to PGRP-LC knockdown? It would be recommended to perform a statistical comparison for the fluorescence intensity of progenitors to prove that the induced knockdowns indeed show lower p38 fluorescence.

We thank the reviewer for this question. We reanalysed our data by measuring the fluorescent intensity in progenitors by slice as requested by Reviewer 3. We find that p-p38 levels are strongly reduced in *PGRP-LE*-depleted progenitors compared to control progenitors in infected midguts. See results from n=1 of 2 experiments below ($p<0.0001$). This is similar to *PGRP-LC*-depleted progenitors in infected midguts (see below, Reviewer 3, point 1).

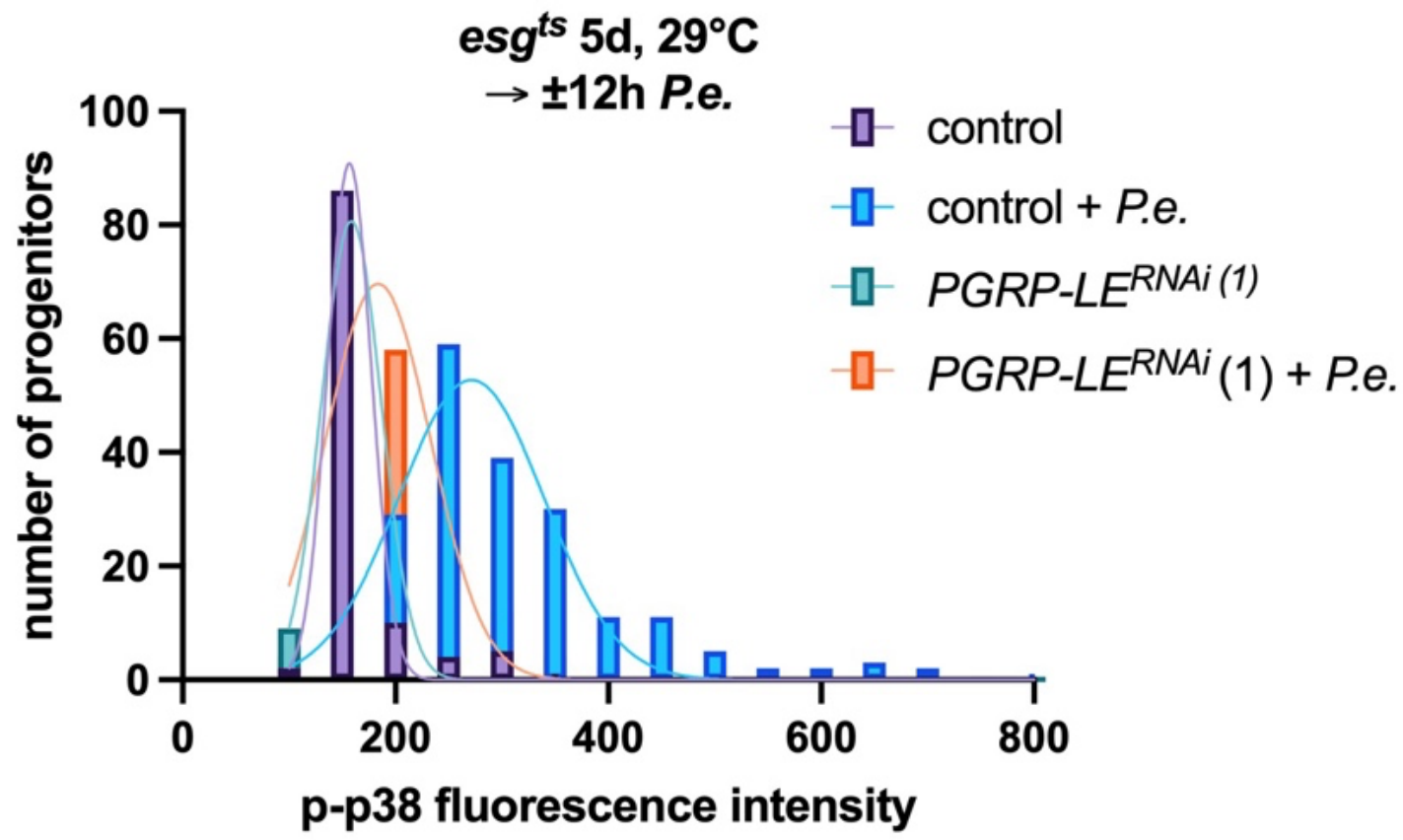

5. Representation of LUT requires normalisation of the fluorescence range, so that minimal and maximal values must be equal in different pictures. This information must be added to the "Methods" section.

We thank the reviewer for pointing this out. We have now included this information regarding LUT generation in our manuscript. We have acquired each stack within an experiment at the same laser intensity and gain. From these stacks, we have generated 8-bit max projections onto which a Fire LUT was applied. We have ensured for each image that the range (minimum and maximum pixel) was set from 0 to 255 in ImageJ prior to applying the LUT. We will, however, present fire LUT images produced from 8-bit single slices as suggested by Reviewer 3 in our revised manuscript.

6. The estimation of P.e. CFUs added into fly tubes is advised to be added to the Method section. The estimated quantity of CFU per fly per mL of food would be the best parameter which would allow to reproduce the methodology by other researchers.

We thank the reviewer for this suggestion. We have now included an estimation of *P.e.* CFU per ml we added to our food vials in our Methods section. We resuspended a pellet of *P.e.* from a 50 ml culture to approximately 7.0 $x10^9$ CFU per ml. We then added 500ul of this solution to the top layer of the food and mixed it into the food to a depth of approximately 3.7 mm.

Significance

This study establishes a foundational model describing a cascade of events that drive intestinal cell renewal in response to bacterial infection. The findings hold clear conceptual significance for specialists in intestinal physiology and innate immunity. Moreover, the proposed model has the potential to evolve into a translational framework relevant to broader biomedical contexts. However, the generalizability of the model may be constrained by its reliance on a specific host species (Drosophila melanogaster) and bacterial strain (Pseudomonas entomophila). Further

functional validation-particularly in alternative microbial contexts and animal systems-will be necessary to assess its broader applicability and evolutionary conservation

We thank the reviewer for the positive evaluation and for stating that our work "establishes a foundational model describing a cascade of events that drive intestinal cell renewal in response to bacterial infection". We believe that our work could conceptually extend beyond the intestine. Pathogenic bacterial sensing by stem cells may promote regeneration in other stem-based epithelial tissues exposed to pathogenic bacteria from the environment, such as the lungs or skin. Moreover, we believe that our observations are likely not limited to specific bacterial strain as both gram-negative *P.e.* and *Ecc15* can activate p38 in the midgut epithelium and stimulate ISC proliferation.

Reviewer #2

Evidence, reproducibility and clarity

This study investigates how oral infection in Drosophila triggers intestinal stem cell (ISC) proliferation. Previous work has shown that reactive oxygen species (ROS), produced by Duox, can lead to ISC renewal following tissue damage. More recent studies implicate the Nox pathway in stem cell activation. This manuscript proposes a novel role for a signaling pathway involving PGRP-LC/LE → Licorne (MKK3) → p38a/b in stem cell proliferation as well as a more broad gutresponse.

This manuscript addresses an important and timely question in gut immunity and stem cell biology. The proposed pathway linking bacterial detection to ISC activation is intriguing and potentially novel. However, additional experiments are critically required to validate key claims and better contextualize the findings. With these revisions, the manuscript could make a strong contribution to the field.

We thank the reviewer for the positive evaluation, and for acknowledging the potential impact of our findings by stating that "the proposed pathway linking bacterial detection to ISC activation is intriguing and potentially novel".

Major Recommendations

1. Validation of RNAi Efficiency and Use of Mutants:

The authors must provide evidence that their RNAi constructs are functional. For instance, PGRP-LC silencing should sensitize flies to Ecc15 during systemic infection. In addition, available null mutants for PGRP-LC and PGRP-LE should be used to validate the RNAi findings and assess their effects on ISC proliferation. Similarly, the use of p38a or p38b mutants would strengthen the claim that these kinases are functionally involved.

We thank the reviewer for these suggestions. We agree with the reviewer that the efficacy of the RNAi lines for *PGRP-LC* and *PGRP-LE* need to be tested. However, we must note that one of the *PGRP-LC* RNAis (VDRC 101636 KK) we used in this study has been used in the following studies: Molaei et al., 2019; *Dev Cell* (Karpac lab), Zhao and Karpac, 2021; *Cell Metab*.,

Mazuzzo et al., 2022; *J. Neurosci.* (Royet lab) and Shin et al., 2022; *Stem Cell Reports* (Foley lab). A *PGRP-LE* RNAi that we used (VDRC 108199KK) was also used in Shin et al., 2022; *Stem Cell Reports*. Unfortunately, the efficacy of these lines was not reported in any of these studies, thus we cannot currently provide this information from these studies. Thus, we will perform qPCR to determine the efficacy of these RNAis to deplete their targets.

We will determine the effects on ISC proliferation in *PGRP-LC* and *PGRP-LE* mutants after *P.e.* infection. We already found a 54.8% decrease in ISC proliferation in *PGRP-LE*^{112} mutants (kind gift from Prof Bruno Lemaitre) after *P.e.* infection compared to control infected midguts. See below (n=1 experiment, p=0.0001). We will repeat this experiment.

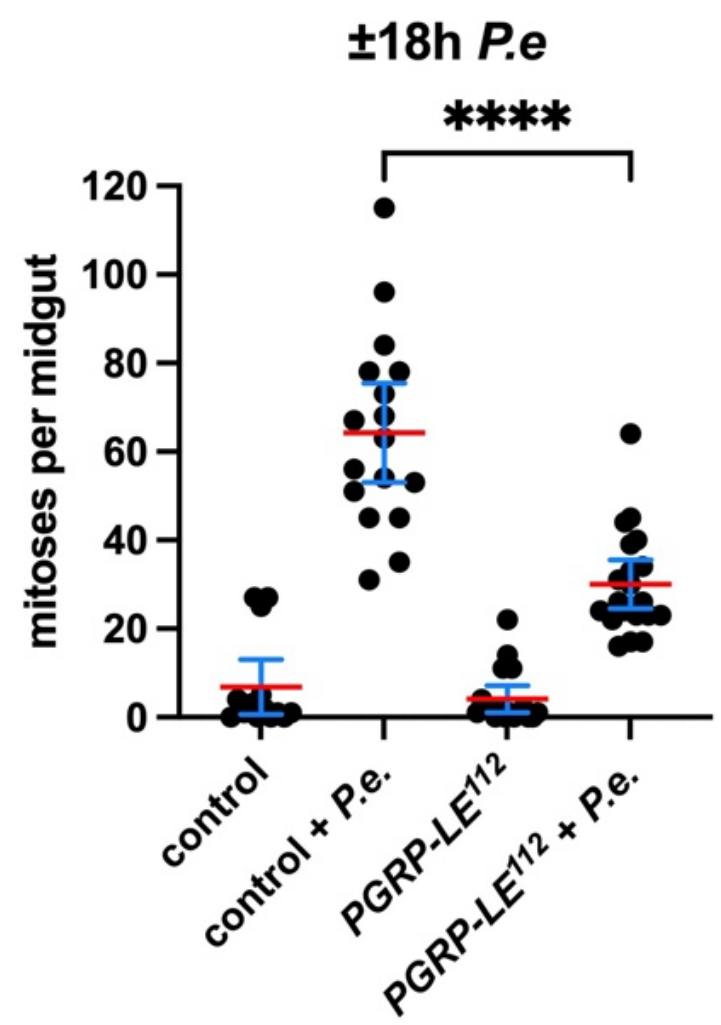

The effects on ISC proliferation in p38a and p38b mutants after *P.e.* infection were determined in Patel et al., 2019. While a significant decrease in ISC proliferation was observed in *P.e.*-infected *p38b*^{ex9} homozygotes, a decrease in ISC proliferation was not observed in *p38a*^{1} mutants. We will discuss these results in our Discussion.

2. Demonstrating the Role of Peptidoglycan:
The article imply that peptidoglycan is the ligand triggering stem cell proliferation via PGRP-LC/LE. However, this has not been directly shown. This point should be removed from the subtitle and discussed more cautiously. It remains possible that PGRP-LC/LE is activated by other microbial or host-derived ligands. The authors should test whether feeding flies DAP-type peptidoglycan or TCT (tracheal cytotoxin) stimulates stem cell proliferation. Furthermore, do heat-killed bacteria induce the same response? If not, additional signals may be required for full activation.

We thank the reviewer for raising this important point and for these suggestions. We agree that we have not shown that peptidoglycan alone can activate p38 in the midgut epithelium and ISC proliferation. Thus, we will feed flies DAP-type peptidoglycan or tracheal cytotoxin (TCT) to determine whether this stimulates ISC proliferation. If it does not, it may suggest that additional

microbial or host-derived signals are required for p38 activation. In this case, we will remove peptidoglycan from the subtitle and discuss the possibility that, together with peptidoglycan, other microbial and host-derived ligands may help trigger p38 activation in ISCs and their proliferation.

We have, however, fed heat-killed *P.e.* to adult flies. To our surprise, heat-killed (95ºC) *P.e.* still damaged the midgut and stimulated ISC proliferation (see below). We confirmed that our heat-killed *P.e.* were indeed not viable. Thus, we currently believe that a *P.e.* virulence factor, possibly Monolysin, is resistant to heat treatment and is still active. Interestingly, we found that *PGRP-LC* was required in progenitors for ISC proliferation (81.4% decrease) after ingestion of heat-killed *P.e.*. See below for *PGRP-LC* (n=1 experiment, p<0.0001). We similarly found that *PGRP-LE* was required in progenitors for ISC proliferation after ingestion of heat-killed *P.e.*. These data suggest that midgut progenitors can sense the peptidoglycan of heat-killed *P.e.* via PGRP-LC and PGRP-LE.

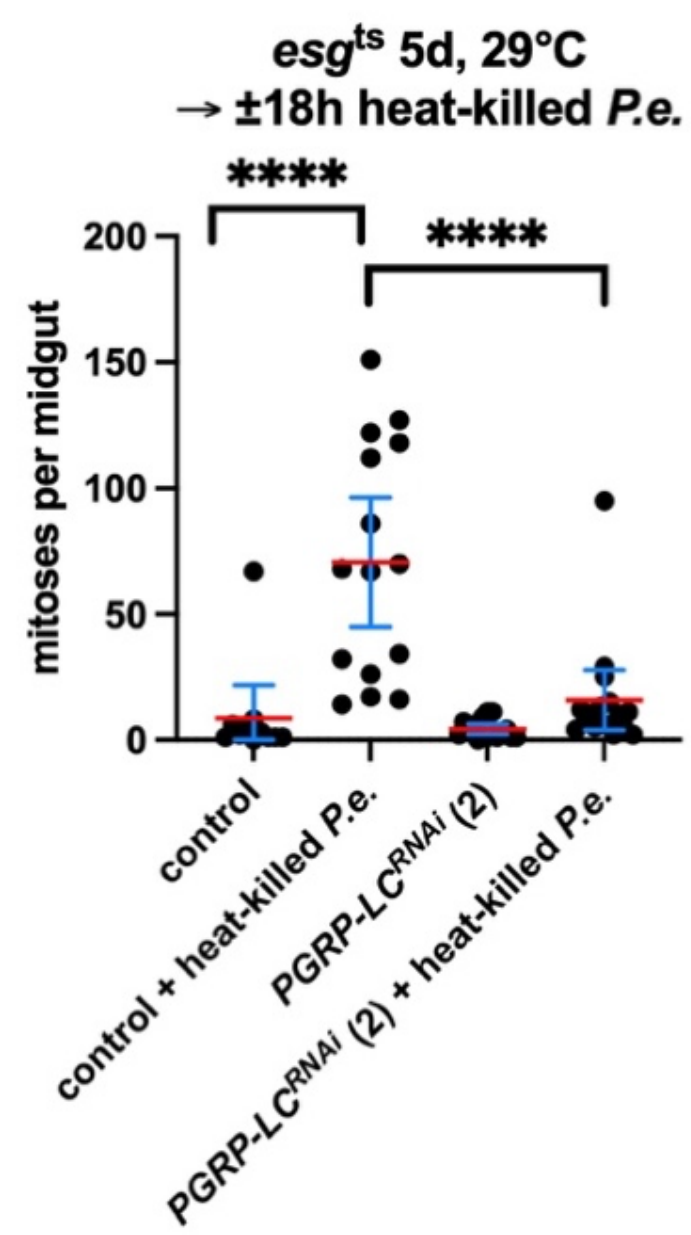

3. Is the Imd Pathway Involved?:
The authors outline a pathway involving PGRP-LC/LE → Lic → p38 → ISC proliferation. It would be informative to determine whether this pathway also requires Imd or Relish. Notably, prior studies suggest that Relish mutants display increased ISC proliferation. This point should be addressed experimentally using mutants and RNAi.

We thank the reviewer for these interesting suggestions. Indeed, we had started to test whether Imd is required in progenitors for p38 activation in the midgut epithelium and ISC proliferation. We found that depleting *imd* in progenitors inhibited infection-induced ISC proliferation (p=0.0130) and p38 activation in progenitors (p<0.0001) and in the midgut epithelium (75.4% decrease; p=0.0269). See below (n=1 experiment). We used the VDRC *imd* RNAi line

101834KK used in Zhai et al, 2018; *Cell Host and Microbe* (Lemaitre lab) and Krautz et al, 2020; *eLife* (Theopold lab). These data suggest a possible role for *Imd* in progenitors for ISC proliferation and p38 activation after infection. We will repeat this experiment. We will further test a role for Imd in progenitors for trans-epithelial p38 activation and ISC proliferation using an *imd* null mutant (*imd*[1]).

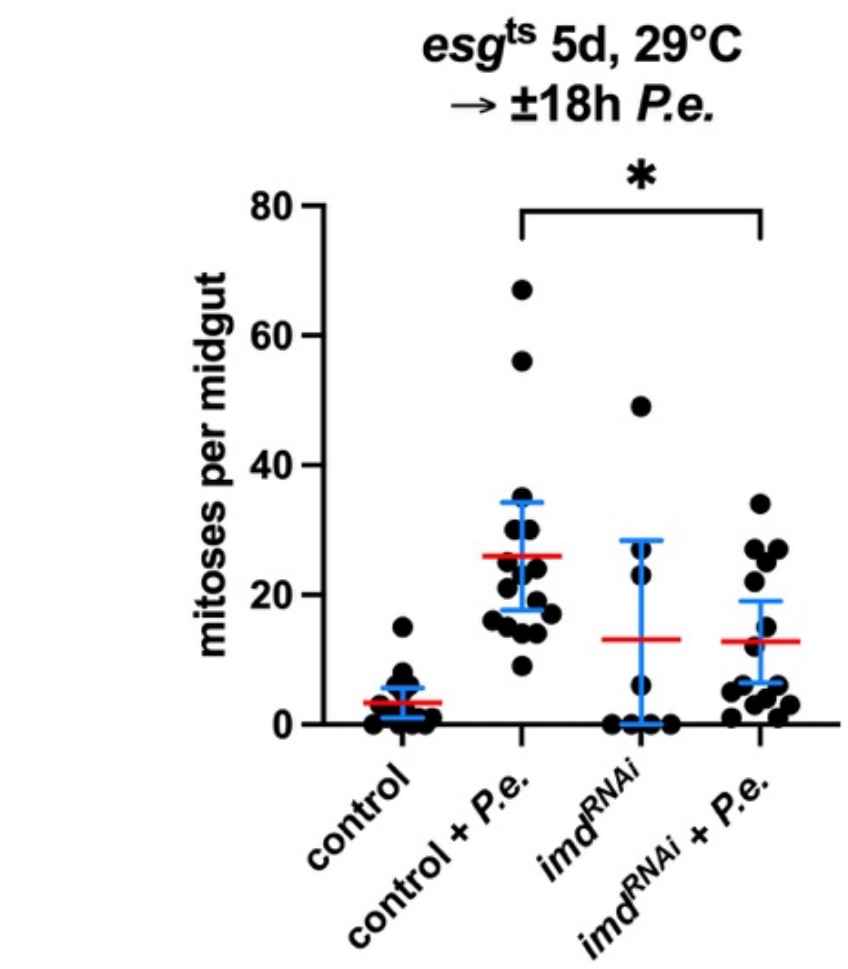

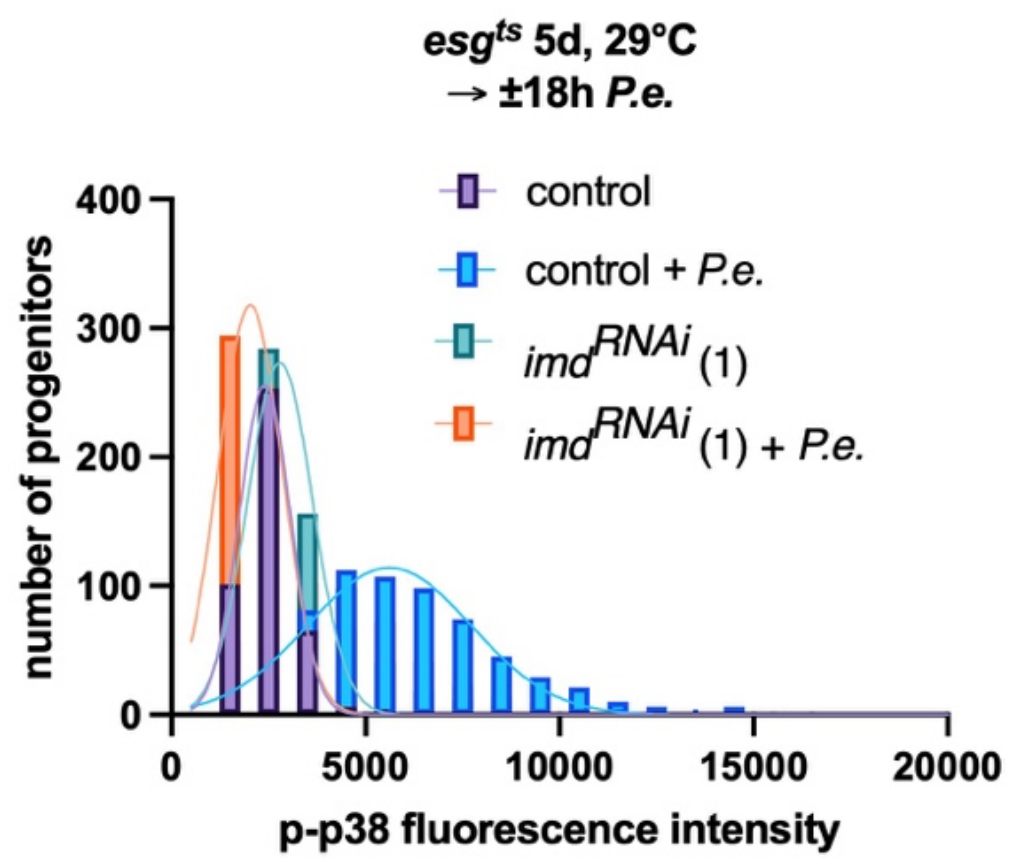

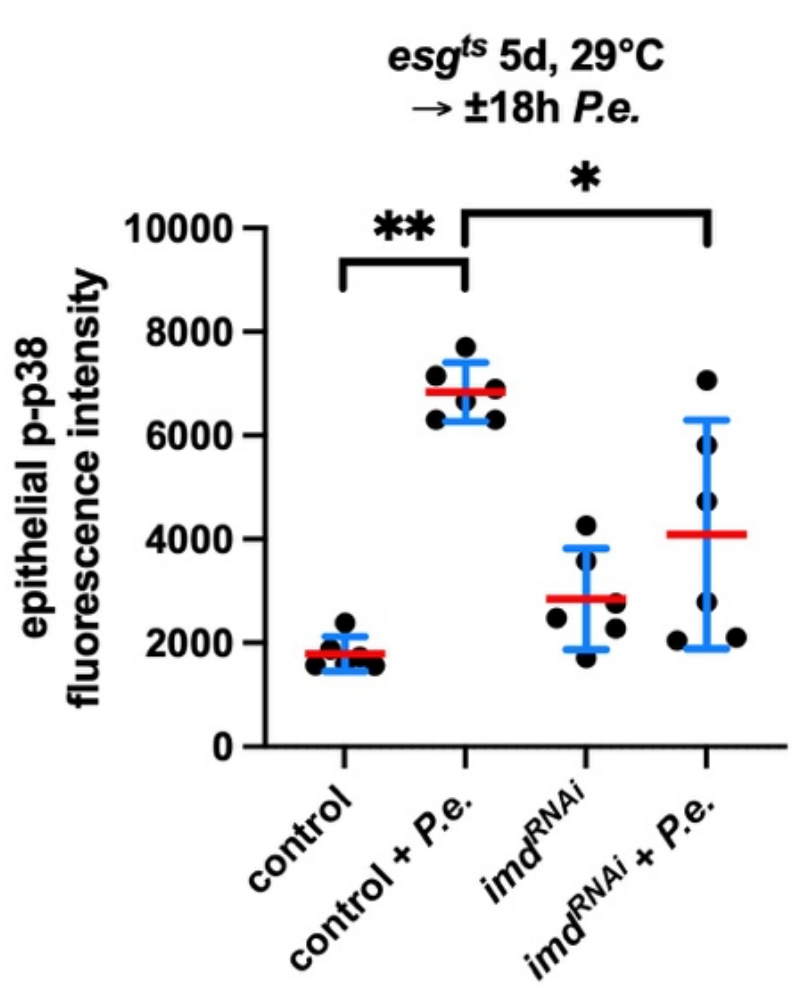

It is not clear, however, how Relish (NF-ϰB) would regulate p38 activity, since PGRP signalling is thought to regulate Relish independently of p38 in midgut enterocytes. Previous studies do report increased ISC proliferation in *Relish* mutants. As PGRP signalling induces Relish activity, these results are contrary to our data showing a decrease in ISC proliferation in *P.e.*-infected *PGRP-LE*^{112} flies (above, point 1) and in *P.e.*-infected midguts expressing RNAis toward *PGRP-LC* or *PGRP-LE* in progenitors. Nevertheless, using *Relish* mutants, we will examine whether Relish is involved in the activation of p38 signalling in the midgut epithelium and stimulation of ISC proliferation after infection.

Minor Recommendations
1. Page Numbering:
Please number the pages to facilitate the review process.

We apologise for this oversight. We have now added page numbers to the manuscript.

2. Clarification of Previous Literature:
Note the findings from Chakrabarti et al. (PLoS Genetics, 2014), which questioned the role of PGRP-LC in Duox activation:
"Using null mutations (Mekk1^Ur36, PGRP-LC^E12) and an RNAi construct targeting Mkk3, we found no effect of PGRP-LC on Duox transcription following P. entomophila infection, though Mkk3 and Mekk1 were required (Figure 4C)."

We thank the reviewer for pointing this out. We are aware of this study but forgot to mention it in our Introduction. We apologise for this oversight. We will include this information in the Introduction of our revised manuscript.

3. Introduction:
Please specify that Nod sense 'Gram'-negative bacterium.

We apologise for this typo. We have now edited this in our revised manuscript.

4. Typo Correction:
"RKP2" should be corrected to "RIPK2."

We apologise for this typo. We have now edited this in our revised manuscript.

5. Clarify Expression of Fold Change:
Replace ambiguous expressions such as "induced by 134%" with clearer alternatives like "increased by 134%."
"Induced by 180%" is unclear; "increased by 180%" is the correct standard.

We thank the reviewer for pointing this out. We completely agree and have edited these in our revised manuscript.

6. Use of ISC-Specific Drivers:
The current study uses esgGAL4 tubGAL80^ts Su(H)Gal80 to manipulate gene expression in ISCs and enteroblasts. To increase specificity, the use of Delta-GAL4 to target ISCs alone would strengthen the conclusions.

We thank the reviewer for this suggestion. The *esgGAL4 tubGAL80^{ts}* system allows for progenitor-specific GAL4-mediated expression. However, *esgGAL4 tubGAL80^{ts} Su(H)Gal80* is an ISC-specific driver as it blocks GAL4-mediated expression in *Su(H)$^{+}$* enteroblasts. Data using this ISC-specific driver can be found in Figure 1F, which shows that p38a and p38b signalling are key in ISCs to promote their proliferation after *P.e.* infection. Indeed, we have recently used this stock to deplete *p38a* and *p38b* in ISCs and found that p38 activation is blocked by 92.4% in the midgut epithelium after *P.e.* infection (see below n=1 experiment; p=0.0043). These data suggest that p38a and p38b signalling is key in ISCs of infected midguts to promote trans-epithelial p38 activation. We will repeat this experiment. Using the ISC-specific driver, we will also test if PGRP-LC or LE are required in ISCs for their proliferation after *P.e.* infection. We did not use Dl-GAL4 in this study because we found that very few ISCs expressed GAL4 in these midguts. We found this to be the case for Dl-GAL4 stocks from two different laboratories.

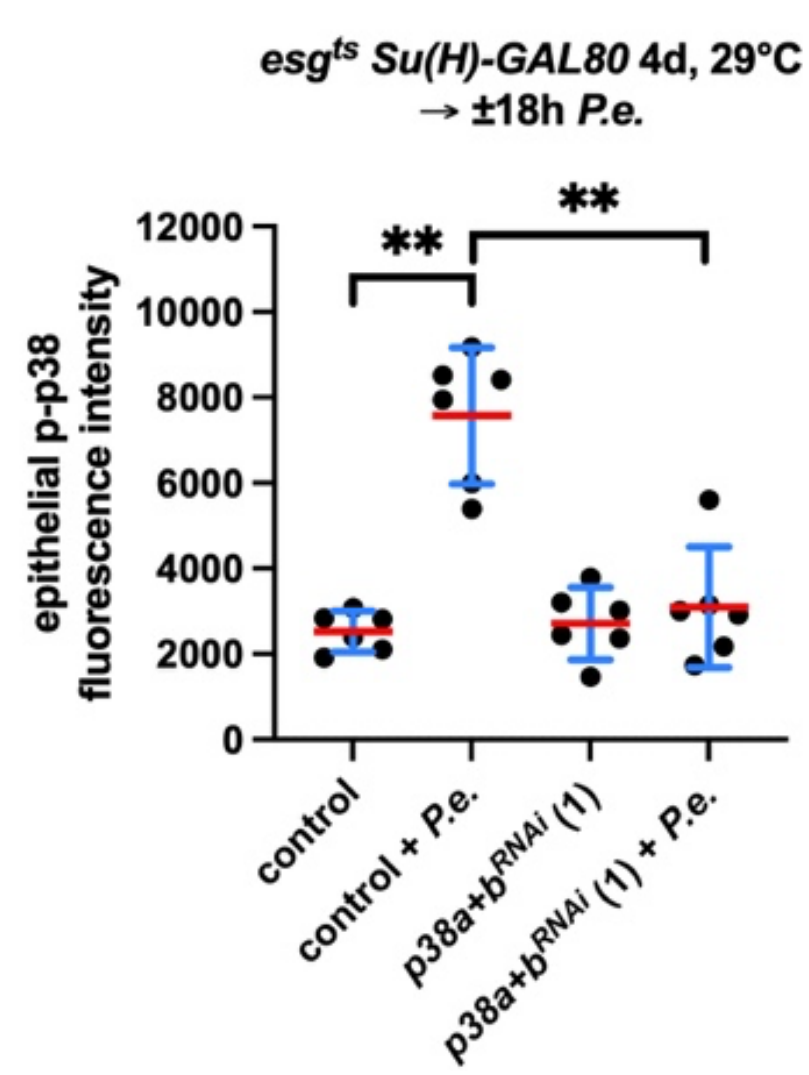

7. Background on MAPK-p38 Pathway:
A brief explanation of the p38 MAPK pathway and its relevance in immune/stem cell signaling would be helpful, especially to justify the focus on Licorne.

We thank the reviewer for this suggestion. We completely agree that a brief explanation of the importance of p38 MAPK signalling in immune and stem cell signalling will be helpful to the reader. We will add this to the Introduction of our revised manuscript.

8. Figure Legends and Labels:
For clarity, instead of using "Licorne " label figures with driver and gene names (e.g., esg>Licorne) to make the experiments more accessible to readers.

We thank the reviewer for this suggestion. However, we have already included both the driver and gene/RNAi name in each panel. We haven't used, for example, *esg*^{ts}>Licorne, because the label often ends up obscuring data.

9. PGRP-LE Forms:
Note that the existence of a secreted/extracellular form of PGRP-LE remains unconfirmed. See: https://reprosci.epfl.ch/

We thank the reviewer for pointing this out. We will amend the text accordingly.

10. Materials and Methods:
This section should be more detailed. Please specify fly age, sex, , infection protocols (starvation conditions (if any)),.

We thank the reviewer for these suggestions. We have already mentioned in our Methods the age and sex of the flies used in our experiments. However, we have now added to our Methods more detailed information about our infection protocol.

11. Genotypes and Double RNAi Experiments:
The genotypes used, especially (ex double RNAi experiments on.p38a and p38b knockdown), should be fully described.

We thank the reviewer for pointing this out. We will make a list of the genotypes used in our study in our Supplementary Materials. The p38a and p38b depletion does not use two independent RNAi lines. Two RNAi lines at VDRC can simultaneously target both p38a and p38b due their extensive homology. qPCR data shows that these RNAis can deplete both p38a and p38b transcripts. See Patel et al., 2019, Supplementary Fig. 1j.

Significance
This manuscript addresses an important and timely question in gut immunity and stem cell biology. The proposed pathway linking bacterial detection to ISC activation is intriguing and potentially novel. However, additional experiments are critically required to validate key claims and better contextualize the findings. With these revisions, the manuscript could make a strong contribution to the field.

We thank the reviewer for the positive evaluation of our work and for stating that "the proposed pathway linking bacterial detection to ISC activation is intriguing and potentially novel". We hope that our revisions will allow our work to make a strong contribution to the field.

Reviewer #3 (Evidence, reproducibility and clarity (Required)):

Uttekar et al. present evidence of direct sensing of bacterial pathogens by the progenitor population of the adult Drosophila midgut. They delineate the molecular pathway that allows this detection and links it to a proliferative response, identifying DAP-type proteoglycan receptors PGRP-LC and LE as receptors, and the MKK3 > p38 pathway as the intracellular transducer that leads to proliferation. This is a very interesting observation that changes considerably the perspective on how the epithelial response to bacterial infections is organised. So far, the view of infection defence has been dominated by a strong separation of roles, where terminally differentiated cells (mostly enterocytes) did all the sensing (directly or through damage), mounted an anti-microbial response, and stimulated proliferation of the progenitor population through secreted factors. Progenitors were there just to provide new cells. The results presented here dynamite this view, and open important new questions about the extent to which progenitors contribute to the infection response. For instance, it is clear that ISCs not only participate in sensing, but also function to stimulate p38 activation in nearby ECs, which can autonomously both detect the pathogens and activate p38 through another pathway. The authors make a compelling case with their experimental approach and the data, but the paper is a bit thin on mechanism.

Overall, the phenomenology is of sufficient interest to be disseminated widely and soon. On the other hand, the authors have not made a full attempt at dissecting the mechanism with the same level of detail that one could expect in Drosophila. While I do not think this is necessarily a problem, I have a few caveats with the characterisation of the phenomenology.

We thank the reviewer for the positive evaluation and for stating that our "very interesting observation changes considerably the perspective on how the epithelial response to bacterial infections is organised".

Main scientific comments:

1 __ Fluorescence of phospho-p38.

The authors claim that according to the results in Figure 1A-D, the levels of phospho-p38 after P.e. infection in ISCs/EBs are higher than in non-treated conditions. I do not doubt the

results, but I think several aspects of their reporting needs clarification:

- Imaging conditions: making direct comparisons of fluorescence intensity in images originating from different batches of immuno-staining requires, as the authors declare, staining and imaging conditions that minimise variation. However, even with all experimental procedures minimising variation, a normalisation approach is required (i.e. using one normalisation window with no staining per image as within-image normalisation factor). It does not seem that this has been described in methods. Is there a solid reason whereby this is not necessary?

We agree with the reviewer that a normalisation approach is required. We found that the background levels were similar between samples within an experiment, thus we did not report background subtracted measurements. The background (raw integrated density/area) was determined for each sample from an area on the image without any tissue. We have now subtracted the background from the fluorescence intensity (raw integrated density/area) from the tissue. We have now also described this normalisation in the Methods, and we will report these normalised measurements in our revised manuscript.

- Segmentation approach: authors have made projections in the apical-basal axis and measured the intensity in the ROIs resulting from the outline of signal from GFP expressed in progenitors. No reference is made to how the GFP signal is thresholded. Moreover, midgut progenitors are pyramidal cells with a larger basal than apical perimeter. This leads to an overlap with some of the neighbouring ECs when projected along their apical-basal axis. This is quite obvious in Figure 1B-B', where the GFP outlines include slices of high signal that belong to the neighbour ECs. While by eye it is clear that these progenitors have higher levels of signal than the untreated ones from figure 1A-A' anyway, the extent of this difference may have been greatly overestimated if this overlap has been systematically included across the dataset. The presence of this overlap is also so obvious that may cast strong doubts in the reader. One solution would be to measure the signal individually in each of the Z-slices projected (which can be done automatically in ImageJ), and show only one slice in Figure 1A-B.

We thank the reviewer for pointing these issues out.

The GFP signal was auto-thresholded using the Li method in ImageJ. We have added this information to our Methods.

We agree that using stacks of midgut progenitors for p-p38 measurements likely introduces some signal from neighbouring ECs, and this could overestimate the signal within progenitors. We will reanalyse our data by determining the fluorescent intensity (raw integrated density) per progenitor from each of the Z-slices and sum these intensities for each progenitor. We will plot (histogram) the number of progenitors per (summed) fluorescence intensity. Moreover, we will present only one slice in our Figures and include a description of our progenitor signal quantitation in our revised Methods.

Below are some examples of our reanalysis. We found that similar to our previous analysis that blocking ISC proliferation by depleting *string* in progenitors did not inhibit p38 activation within progenitors after *P.e.* infection ($p<0.0001$). In contrast, we found that depleting *PGRP-LC* in progenitors inhibited p38 activation in progenitors after infection ($p<0.0001$). See below (n=1 of 2 experiments shown).

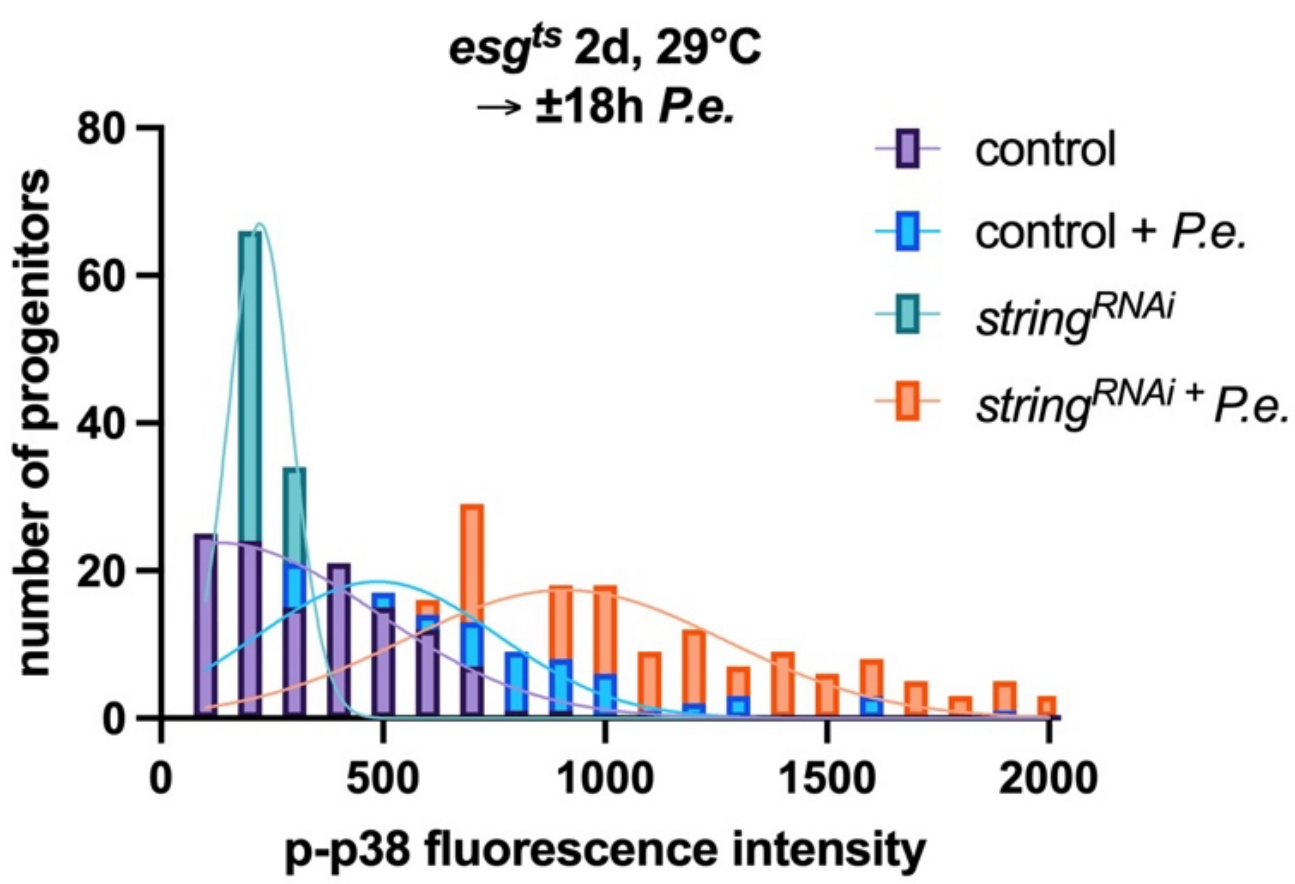

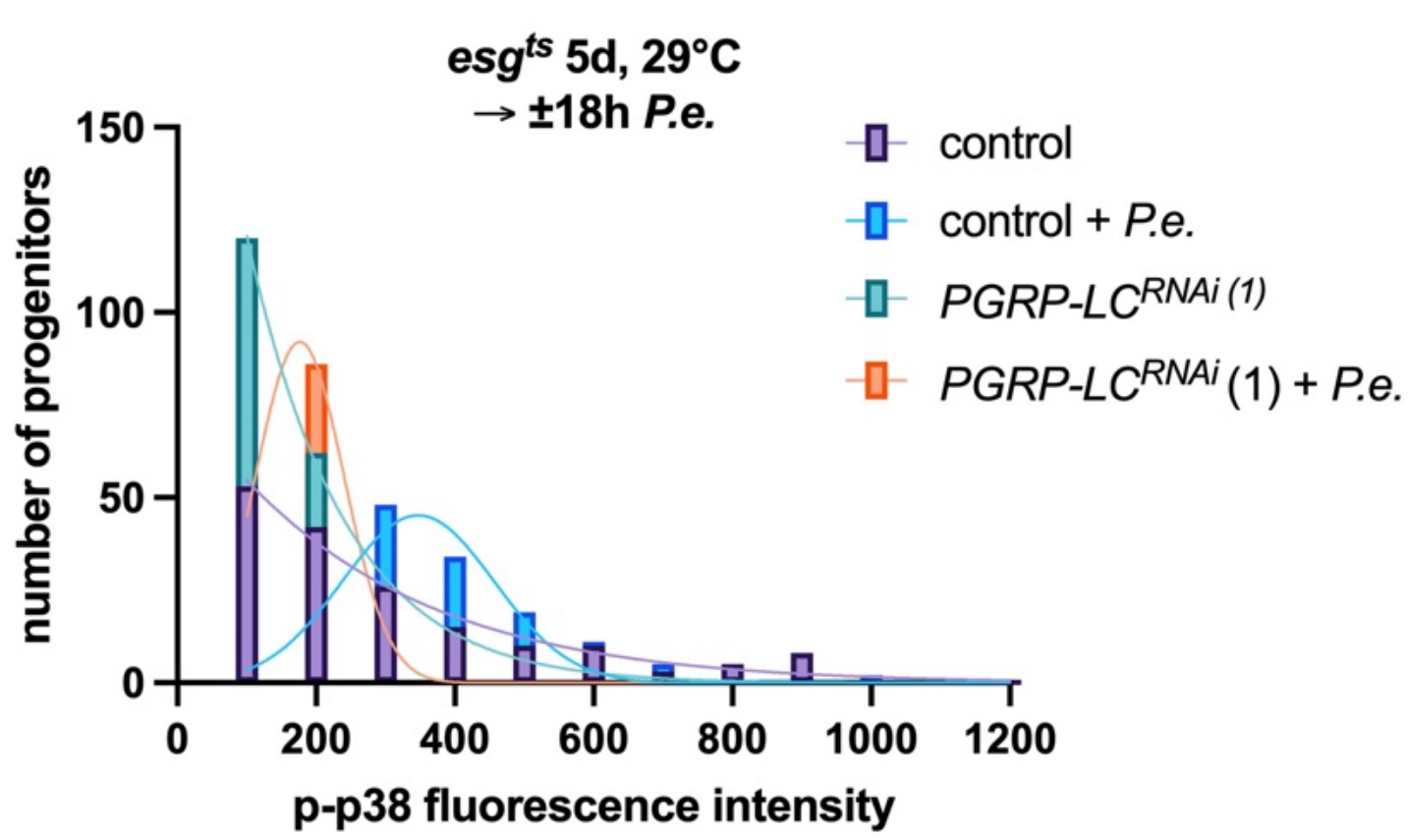

The p-values comparing the p-p38 signal in progenitors from *P.e.*-infected midguts with progenitors depleted for *string* or *PGRP-LC* in *P.e.*-infected midguts when measurements are taken from a projection are $p< 0.0001$ and $p<0.0001$, respectively. These p-values do not differ between the two methods of quantification suggesting that the conclusions we made from our measurements from projections are likely correct.

- Infection with P.e. induces a rapid regenerative response (whose characterization has been in part the contribution of some of the authors of this manuscript). I wonder whether the acceleration of proliferation induces a situation where progenitor cells differentiate terminally faster than the GFP that labels them degrades. This would lead to a situation where they are considering the signal of phospho-p38 in early ECs that are still labelled by GFP molecules that they are no longer producing, but are responding to Pe infection as ECs, not progenitors. If the authors could comment on whether GFP perdurance was apparent in their images and if so, on what precautions have they taken to eliminate from their phospho-p38 dataset cells that were polyploid and/or displaying enlarged cytoplasm, it would make the results more convincing.

We thank the reviewer for pointing this out. It is true that at times there are early ECs that are GFP labelled due to rapid ISC proliferation. To exclude these cells from our p-p38 quantitation, we have used a macro that we developed using images of esg>GFP^+ progenitors from homeostatic midguts to identify cells with a progenitor nuclear size. However, we did not apply this analysis to our images and have outlined all GFP-positive cells. Thus, we will remove outlines around progenitors with nuclei larger than esg>GFP^+ progenitors in homeostatic midguts. We will describe this analysis in our Methods.

We will also make our macro available to download from our lab website for other researchers to use with their research.

These comments apply as well to Figures 2A-C and 3, 4, 5, and 6 (panels B-K).

- More generally, the specificity of the anti-phosphorylated-human p38 does not seem to have been established using RNAi nor mutants. Is this demonstrated in an earlier work? I could not find this information in ref. 41. I am not suggesting to do additional experiments necessarily, but any information the authors have on this would be quite helpful to include

(maybe in methods).

We thank the reviewer for this question. It has been shown that this antibody is specific for phosphorylated p38a and p38b. This antibody was used in Patel et al., 2019 Nat Comms (ref. 41) to examine p38 activation in midgut enterocytes after infection. Simultaneously depleting both p38a and p38b with RNAi in ECs in *P.e.* infected midguts reduced p-p38 levels in the midgut epithelium (see Patel et al., 2019 Suppl. Fig. 1a-b). We have now stated in our Methods that the specificity of these antibodies to p-p38 was tested in reference 41. Furthermore, the depletion of p38b alone in progenitors results in a decrease in p-p38 levels in midgut progenitors by 72.1% after *P.e.* infection compared to control infected progenitors, confirming the specificity of the antibody (see Supplementary Figure 1C).

2 __ Directly addressing the sensing of DAP-PG

The authors delineate a pathway whereby DAP-type proteoglycans stimulate PGRP-LC and PGRP-LE, which in turn activates the cascade Licorne > p38a/b > proliferation/non-autonomous signal propagation. This suggests that the sensing of bacteria is independent of damage and also very sensitive to thresholds (as removing either PGRP strongly suppresses the response). I am surprised that the authors have not attempted to feed the flies DAP-PG to ensure that the response can be triggered in isolation from damage locally or elsewhere. I am not suggesting every single experiment repeated with feeding instead of infection - but some key experiments showing that there is a similar response in the wild type and this is suppressed with e.g. p38a/b RNAi, would strengthen the proposed phenomenology.

We thank the reviewer for this suggestion. We have indeed not yet fed DAP-type peptidoglycan to adult flies. As discussed above (Reviewer 2 point 2), we will feed DAP-type peptidoglycan to adults to determine whether this can trigger p38 activation and ISC proliferation. If so, we will deplete p38a and p38b from progenitors and test whether this inhibits ISC proliferation after DAP-type peptidoglycan feeding.

Minor scientific comments:

3 __ Non cell-autonomous propagation of p38 signalling

Figure 2 shows that overexpression of Licorne induces increased phospho-p38 not only in progenitors, but in some neighbouring ECs. This is not a problem in itself, as the authors show already in Fig 1G and S1D-G that you need activation of the pathway in progenitor cells for ECs to become responsive of it. One can assume that some signal emanates from progenitors to stimulate the pathway in ECs. However, this would suggest some correlation between the levels of p-p38 in ECs and their neighbouring progenitors. Is this the case? The authors could verify this with the existing data.

We thank the reviewer for this question. We will analyse our existing data to determine if there is a correlation between levels of p-p38 in progenitors and their neighbouring ECs. To do this, we will determine and plot the fluorescence intensity in the progenitor and its neighboring ECs to identify a linear relationship between the two. We will also calculate the Pearson correlation coefficient to demonstrate the strength of this relationship.

4 __ Epistasis

The pathway delineation is based on what is known about the molecular function of the proteins and the p-p38 antibody. This is OK, but maybe the authors could consider one epistasis experiment between PGPRs and Licorne or an activated form of p38, to have genetic evidence of the episatsis.

We thank the reviewer for this suggestion. Expressing *PGRP-LC* (or *LE*) RNAi in progenitors blocks ISC proliferation after *P.e.* infection. We will test whether co-expression of Licorne and *PGRP-LC* (or *LE*) RNAi rescues ISC proliferation during infection.

5 __ Mechanistic hints

I do not expect the authors to fully characterise aspects of the mechanism that are clearly missing, like how increased p38 activation leads to proliferation or how progenitors influence p38 activation in neighbouring ECs. However, the authors could analyse existing data to offer some possibilities, even if it is just in the discussion. For instance, the authors could re-analyse existing data of the cell-type specific transcriptional response to Pseudomonas entomophila infection (Dutta et al., 2015) (and existing data from other papers) to make suggestions about potential diffusible signals modulating activation of p38 in ECs or proliferative genes potentially targeted by p38 signalling.

We thank the reviewer for this suggestion. We will, based on the literature, suggest in our revised Discussion possible diffusible signals from progenitors that may activate p38 in ECs. We will likewise suggest potential factors that could be regulated by p38 signalling to promote ISC proliferation.

Language/presentation comments:

Intro:

"Thus, it remains unknown whether midgut cell types, particularly ISCs, can recognise peptidoglycan and contribute directly to regeneration." I would change "recognise peptidoglycan and contribute directly" for "recognise peptidoglycan DIRECTLY TO contribute" as they would contribute _directly_ to regeneration through non-autonomously induced proliferation (arguably they do all the regenerative work as they are the only indefinitely proliferative cell type); the question is whether they do the _sensing_ directly.

We thank the reviewer for this suggestion. We have now edited this text to improve clarity.

"Nod1 senses -negative bacteria" -- I think a 'gram' is missing before '-negative'.

We apologise for this oversight, and we have edited this in our revised manuscript.

"which does not occur upon infection with pathogenic bacteria." I am not sure what is 'Which' referring to. Is it to the regeneration, to the requirement of Nod2 for it or to the ISC and crypt cell depletion resulting from doxorubicin treatment? The next sentence suggests that it is the ISC/crypt cell depletion, but then there is some redundancy between the two.

Indeed, this refers to ISC/crypt cell depletion. We have edited this text in our Introduction.

Results:

"Further, depletion of p38b alone in progenitors blocked p38 activation in the entire midgut epithelium by 62.9% (Figure 1G)." Where it says 'blocked' it should be 'reduced'.

We thank the reviewer for this helpful suggestion. We completely agree with the reviewer and have modified when needed “blocked” to “reduced” throughout the manuscript.

Figure 1G: why is one transgene denominated [RNAi] but the other is [antisense]? Is there a fundamental difference in the constructs that needs to be highlighted like this?

We thank the reviewer for this question. This stock is designated as $p38b^{antisense}$ in Adachi-Yamada et al., 1999. The UAS-$p38b^{antisense}$ construct was made by inserting the full length p38b cDNA but inverted into pUAST. This suggests that the antisense strand is expressed by GAL4. Since the antisense strand likely doesn’t form a hairpin (double stranded RNA) it may block translation of p38b mRNA rather than participating in RNAi-mediated knockdown of p38b mRNA.

Figure S2 title repeats "in progenitors".

We will edit the title for Supplementary Figure 2.

Figure legends in general refer to "control midguts" but it is not clear what is the genotype of such controls. The methods section refers to a KK control line and a UAS-mCherry[RNAi] control line, but it is not specified which one is use as controls in which experiment. Similarly, it is not clear what the controls for UAS-cDNA experiments (e.g. UAS-licorne) are.

We thank the reviewer for these questions. We will make a list of genotypes used for each experiment as requested by Reviewer 2. The KK control line was used when a VDRC KK line was used for an experiment; a UAS-*mCherry* RNAi, when a TRiP RNAi was used for an experiment. Further, w^{1118} was used as a control for UAS-cDNA experiments.

In swarm plots, bars and whiskers are not described in every case. By magnitude, they look SD, but SEM might be more appropriate in some cases (e.g. Fig S1A).

We apologise for this oversight. These are the mean and 95% confidence interval for these plots. However, as stated above in response to Reviewer 1 point 2, we will use descriptive statistics (standard deviation) in the revised manuscript. This information will be described in the revised Figure legends.

In section "PGRP-LC/LE-MKK3-p38 activity in progenitors activates p38 in the midgut epithelium after pathogenic bacterial infection", the authors write: "Thus, we asked whether it was increased ISC proliferation due to increased PGRP-LC/LE- MKK3-p38 signalling in progenitors that stimulated p38 activation throughout the midgut epithelium or whether it was specifically due to p38 signalling." I had to re-read this to work out that the authors suggest a potential 'pathway' such as: [PGPR-LC/LE > Licorne > proliferation > p38 activation] or rather [PGPR-LC/LE > Licorne > p38 activation]. May I suggest that the authors consider editing this paragraph for clarity.

We thank the reviewer for pointing this out. We have edited this paragraph to improve clarity.

Discussion:

The discussion is very repetitive with results (do we really need to read again the percentages with one decimal?) and does not really make an integrated attempt to indicate how the change in perspective impacts our understanding of epithelial immunity/regeneration. The few pointers that are offered could be written more succinctly.

We thank the reviewer for this helpful suggestion. We agree that the Discussion could include more discussion of how the work changes our understanding of midgut epithelial immunity and regeneration. We will amend our Discussion in our revised manuscript.

General:

What is exactly the 'regenerative niche' for the authors? Which cells and/or other anatomical entity?

We thank the reviewer for this question. We have defined the regenerative niche (or microenvironment) as all the midgut cell types that influence ISC behavior, such as proliferation, after damage. These include midgut epithelial cells such as EBs, ECs and EEs, the visceral muscle and trachea. This is described in the Introduction on page 2.

Referee cross-commenting

All comments by the other reviewers seem sensible to me. I think evaluating the involvement of the Imd pathway, while it would make the paper certainly stronger, may be construed as slightly out of scope.

We will determine whether *Imd* is required in progenitors for ISC proliferation and trans-epithelial p38 activation after pathogenic infection. Our preliminary data suggest a role for *Imd*. See Reviewer 2, point 3.

Reviewer #3 (Significance (Required)):

Uttekar et al. present evidence of direct sensing of bacterial pathogens by the progenitor population of the adult Drosophila midgut. They delineate the molecular pathway that allows this detection and links it to a proliferative response, identifying DAP-type proteoglycan receptors PGRP-LC and LE as receptors, and the MKK3 > p38 pathway as the intracellular transducer that leads to proliferation. This is a very interesting observation that changes considerably the perspective on how the epithelial response to bacterial infections is organised. So far, the view of infection defence has been dominated by a strong separation of roles, where terminally differentiated cells (mostly enterocytes) did all the sensing (directly or through damage), mounted an anti-microbial response, and stimulated proliferation of the progenitor population through secreted factors. Progenitors were there just to provide new cells. The results presented here dynamite this view, and open important new questions about the extent to which progenitors contribute to the infection response. For instance, it is clear that ISCs not only participate in sensing, but also function to stimulate p38 activation in nearby ECs, which can autonomously both detect the pathogens and activate p38 through another pathway. The authors make a compelling case with their experimental approach and the data, but the paper is a bit thin on mechanism.

Overall, the phenomenology is of sufficient interest to be disseminated widely and soon. On the other hand, the authors have not made a full attempt at dissecting the mechanism with the same level of detail that one could expect in Drosophila. While I do not think this is necessarily a problem, I have a few caveats with the characterisation of the phenomenology.

We thank the reviewer for the very helpful and constructive review that will further improve our work.

Revision Plan

3. Description of the revisions that have already been incorporated in the transferred manuscript

Please insert a point-by-point reply describing the revisions that were already carried out and included in the transferred manuscript. If no revisions have been carried out yet, please leave this section empty.

1. We have now stated in our figure legends and Methods that phospho-p38 levels were analysed in regions R4a and R4b from randomly chosen midguts within a sample (Reviewer 1).
2. We have included information regarding LUT generation in our Methods. (Reviewer 1)
3. We have now included an estimation of *P.e.* CFU per ml we added to the fly vial in our Methods. (Reviewer 1)
4. We have now added page numbers to the manuscript. (Reviewer 2)
5. We have corrected the typos “gram-negative” and “RIPK2” in the Introduction. (Reviewers 2 and 3)
6. We have changed “induced” to “increased” throughout the manuscript when appropriate. (Reviewer 2)
7. We have added to our Methods detailed information about our infection protocol. (Reviewer 2)
8. We have included in the Methods how we normalised our p-p38 fluorescence intensity measurements. (Reviewer 3).
9. We have indicated in our Methods that the GFP signal was auto-thresholded using the Li method in ImageJ. (Reviewer 3)
10. We have described in our Methods how we excluded GFP^+ early ECs from our progenitor p-p38 analysis.
11. We have indicated in our Methods that the specificity of the p-p38 antibodies was tested in reference 41. (Reviewer 3)
12. We edited the text in our Introduction (page 3) and Results (page 6) to improve clarity as requested by Reviewer 3.
13. We have modified when appropriate “blocked” to “reduced” throughout the manuscript. (Reviewer 3)

14. Description of analyses that authors prefer not to carry out

Please include a point-by-point response explaining why some of the requested data or additional analyses might not be necessary or cannot be provided within the scope of a revision. This can be due to time or resource limitations or in case of disagreement about the necessity of such additional data given the scope of the study. Please leave empty if not applicable.

1) We will not provide the total number of midgut cells and the total number of GFP-positive cells in a supplementary table for each experiment. Since this is not a common practice

within our field, we have not collected this information from our experimental samples and it would require re-performing all of our experiments (Reviewer 1, point 1)
