# Supplementary material for: Recognition of pathogenic bacteria by intestinal stem cells promotes adult Drosophila midgut regeneration
Source: PLoS Biol. 2026 Aug 25;24(8):e3003951. doi: 10.1371/journal.pbio.3003951 (PMC13541249; doi:10.1371/journal.pbio.3003951)
Supplement: Supplementary file 5 [file pbio.3003951.s015.docx]

We would like to thank the reviewers for their helpful suggestions, which have clarified and strengthened our manuscript. We have carefully addressed each comment with clarifications and, where appropriate, additional experiments. Overall, our model and conclusions remain the same after the additional tests outlined below.

We have also added new elements to our model. While we previously showed that progenitors are able to sense pathogenic bacteria and elicit an ISC regenerative response, it was not clear which progenitor sense pathogenic bacteria or whether they do so equally. We have now found that PGRP-LC is strongly required in intestinal stem cells (ISCs) to promote ISC proliferation and midgut epithelial p38 activation. Further, we find a strong requirement for p38a and p38b in ISC proliferation, and for p38 activation in the midgut epithelium. Loss of PGRP-LC in EBs only leads to mild reductions in ISC proliferation and midgut epithelial p38 activation. These data indicate the ISCs are the principal sensors of pathogenic bacteria and transducers of p38 activation across the midgut epithelium. This is further supported by our finding that p38a/b signalling is not required in EBs to promote ISC proliferation.

Further, we have found that Imd signalling is required in progenitors to promote p38 activation in both progenitors and throughout the midgut epithelium as well as ISC proliferation after pathogenic bacterial infection.

**Reviewer #1**

**Evidence, reproducibility and clarity**

Summary:
This research article demonstrates that intestinal stem cells (ISCs) in the adult female Drosophila melanogaster midgut can detect peptidoglycans from the Gram-negative bacterium Pseudomonas entomophila through the PGRP-LC/LE receptors. This detection triggers MKK3-p38 signaling in both intestinal progenitors and enterocytes, promoting ISC proliferation. The authors' conclusions are well supported by the presented experimental data, which are clearly described and thoughtfully discussed in the context of existing literature. They also suggest future research directions and consider the evolutionary implications of their findings. Notably, it remains unclear how peptidoglycan sensing in progenitors leads to p38 activation in enterocytes. This signaling mechanism may be specific to certain bacteria, such as P. entomophila, and could require additional cues not present in other species.

We thank the reviewer for their positive evaluation of our work and for noting that our “conclusions are well supported by the presented experimental data”.

Major points (optional):
To strengthen this point, the authors could optionally extend their study by including experiments with Erwinia carotovora carotovora 15 (Ecc15) and Acetobacter species-at least assessing mitotic activity and p38 activation. Further functional analyses might also clarify how bacterial signals trigger ISC activation and how ISCs coordinate with enterocytes to induce p38 signaling and downstream responses. Additionally, investigating enterocyte turnover and their replacement dynamics by proliferating progenitors could enrich the mechanistic insight. Nonetheless, the current data are sufficient to support the central claims of the manuscript. The methodology and data analysis are sound and appropriate for substantiating the reported phenomena.

We thank the reviewer for these suggestions. We believe that the induction of PGRP-MKK3-p38 signalling is not specific to certain bacteria (*P.e.*), since previous studies have shown that *Ecc15* can activate p38 in the adult *Drosophila* midgut epithelium (Ha et al., 2009; *Nature Immunology*) and induce ISC proliferation (Buchon et al., 2009; *Genes and Dev*). We have added this information to our Discussion. Nevertheless, we have now also shown that after *Ecc15* infection, PGRP-LC is required in progenitors to activate both ISC proliferation and p38 activation in the midgut epithelium (S2K-L Fig.). This data excludes the possibility that additional *P.e*.-specific factors are required for PGRP-LC signalling to promote ISC proliferation. Instead, it suggests that DAP-type peptidoglycan, possibly along with other bacterial or host factors (see Reviewer 2, point 2), can trigger ISC proliferation after pathogenic bacterial infection. We would be interested in extending this analysis further to other gram-negative species such as Acetobacter in the future.

We agree with the reviewer that it remains unclear how peptidoglycan sensing in progenitors leads to p38 activation in enterocytes. We now propose some potential mechanisms in our Discussion (see Reviewer 3, point 5), and we hope to investigate these further in the future. Moreover, to further clarify how bacterial signals trigger ISC activation, we have investigated possible roles for Imd and Relish (see Reviewer 2, point 3). We found Imd activity is necessary in progenitors to stimulate ISC proliferation and p38 activation throughout the midgut epithelium after *P.e.* infection (Fig. 4; S4C-D Fig.). However, we did not observe a consistent decrease in ISC proliferation in *imd^1^* mutant midguts after *P.e.* infection (S4A-B Fig.). Additionally, we found that *Rel* is not required for ISC proliferation after *P.e.* infection using the *Relish* mutant (*Rel^E20^*) (S4F Fig.). Together, these data suggest that PGRP-LC in progenitors activates p38 signalling and ISC proliferation through Imd but not Relish signalling. Lastly, enterocyte turnover and replacement after *P.e.* infection have been reported in Jiang et al, 2009; *Cell*. However, if we were able to activate p38 and ISC proliferation with DAP-type peptidoglycan feeding (see Review 2, point 2 and Reviewer 3, point 2), it would have been interesting to examine whether peptidoglycan alone could influence epithelial turnover dynamics. We thus fed 5mg/ml DAP-type peptidoglycan from *E. coli*, but this did not stimulate ISC proliferation, suggesting that other cues together with DAP-type peptidoglycan, either bacterial or from the host, are required to trigger ISC proliferation.

Minor points (to be addressed):
1. Some measured parameters seem to be analysed in midgut regions, while the number of mitoses were measured in the whole midgut. In cases when particular gut regions were selected, figure legend should be adjusted accordingly, with explanation which midgut regions were analysed, and the method section should describe which measures the authors undertook to avoid biases due to differences between physiological regions of the midgut. In cases when the whole midgut was analysed, the total number of midgut cells, and the total number of GFP-positive cells for each midgut ought to be indicated in an additional supplementary table.

We thank the reviewer for this suggestion. Activated p38 (p-p38) is frequently observed in unchallenged midguts in the anterior midgut (regions R1 and anterior R2) and occasionally in and around the mid-midgut (R3). In contrast, p38 activity is generally absent in the posterior midgut (R4–R5), where only occasional low levels of p38 activation are detected (Patel et al., 2019; *Nature Commun.*). Thus, we analysed regions R4a and R4b in this study. Regions R4a and R4b were imaged from randomly chosen midguts within a sample. We have now added this information to our figure legends and the Methods.

The number of mitoses were, however, quantitated throughout the entire midgut. It is not clear why the total number of midgut cells and the total number of GFP-positive cells are suggested to be provided in a supplementary table. This is not a common practice within our field; thus, we have not collected this information from our experimental samples. See these papers: Jiang *et al.*, 2009; *Cell* (Edgar lab), Buchon *et al.*, 2009; Genes and Dev (Lemaitre lab); Hudry *et al.*, 2016; *Nature* (Miguel-Aliaga lab); Patel *et al*., 2019; *Nat Commum* (Edgar lab); Perochon *et al*., 2021; *Nat Cell Biol* (Cordero lab)*;* Petsakou *et al*., 2023; *Nature* (Perrimon lab). If the reviewer is concerned whether *RNAi* expression and *P.e.* infection led to decreased progenitor numbers, we have provided this analysis within a portion of R4a and R4b in S2I-J, S3A-B, S4E, S5F-G, S6A Figs.. We found in all of our experiments that *RNAi* expression in progenitors and *P.e.* infection did not negatively affect midgut progenitor numbers.

2. Statistical analyses should be described more cautiously when small sample volumes are presented (especially for n<10). In cases of n = 5 or less, representation of mean and 95%CI on graphs is meaningless and statistically wrong. Corrections for multiple comparisons should be taken into consideration when such analysis was conducted (Fig 1 G, for example)

We thank the reviewer for pointing this out. We have consulted a data scientist at the University of Bristol, who suggested we use descriptive statistics rather than inferential statistics to describe our data. Thus, we will only include the standard deviation in graphs displaying data per midgut. We will edit our graphs and figure legends in our revised manuscript. Moreover, we haven’t performed multiple comparisons between samples within an experiment. We report here only comparisons between two independent groups.

3. In graphs where the number of progenitors with different fluorescence intensity is represented (e.g. Fig 1 C), the range of intervals for each fluorescent group should be noted in the legend. Otherwise it is not clear why there are white spots between histogram columns (supposedly, continuous range of fluorescence intensity was initially split into intervals with no gaps).

We thank the reviewer for this suggestion. We have included these ranges in our revised figure legends. For example, in prior Fig 1C, the first bar represents intensity values binned from 0-500 and each subsequent bar, every 1000. We have now re-quantified the fluorescent intensities within these progenitors using single slices as suggested by Reviewer 3 and plotted histograms to report their distribution. These histograms can be found in the S1-3 App..

4. How do the authors describe higher activation of p38 in PGRP-LE knockdown in comparison to PGRP-LC knockdown? It would be recommended to perform a statistical comparison for the fluorescence intensity of progenitors to prove that the induced knockdowns indeed show lower p38 fluorescence.

We thank the reviewer for this question. We reanalysed our data by measuring the fluorescent intensity in progenitors by slice as requested by Reviewer 3. We found that p-p38 levels are strongly reduced in *PGRP-LE*-depleted progenitors compared to control progenitors in infected midguts (Fig. 2I). This is similar to *PGRP-LC*-depleted progenitors in infected midguts (Fig. 2B). See S1 App. for histograms and statistics.

5. Representation of LUT requires normalisation of the fluorescence range, so that minimal and maximal values must be equal in different pictures. This information must be added to the "Methods" section.

We thank the reviewer for pointing this out. We have now included this information regarding LUT generation in our manuscript. We have acquired each stack within an experiment at the same laser intensity and gain. From these stacks, we have generated 8-bit max projections onto which a Fire LUT was applied. We have ensured for each image that the range (minimum and maximum pixel) was set from 0 to 255 in ImageJ prior to applying the LUT. We have, however, now presented fire LUT images produced from 8-bit single slices as suggested by Reviewer 3 in our revised manuscript. We have reconfirmed our methods with our resident imaging processing and analysis expert.

6. The estimation of P.e. CFUs added into fly tubes is advised to be added to the Method section. The estimated quantity of CFU per fly per mL of food would be the best parameter which would allow to reproduce the methodology by other researchers.

We thank the reviewer for this suggestion. We have now included an estimation of *P.e.* and *Ecc15* CFU (bacteria per ml) we added to our food vials in our Methods section. We resuspended a pellet of *P.e.* or *Ecc15* to approximately 7.0 x10^9^ CFU per ml and 3.8 x10^11^ bacteria/ml, respectively. We then added 500μl of this solution to the top layer of the food and mixed it into the food to a depth of approximately 3.7 mm.

**Significance**

This study establishes a foundational model describing a cascade of events that drive intestinal cell renewal in response to bacterial infection. The findings hold clear conceptual significance for specialists in intestinal physiology and innate immunity. Moreover, the proposed model has the potential to evolve into a translational framework relevant to broader biomedical contexts.

However, the generalizability of the model may be constrained by its reliance on a specific host species (Drosophila melanogaster) and bacterial strain (Pseudomonas entomophila). Further functional validation-particularly in alternative microbial contexts and animal systems-will be necessary to assess its broader applicability and evolutionary conservation

We thank the reviewer for the positive evaluation and for stating that our work “establishes a foundational model describing a cascade of events that drive intestinal cell renewal in response to bacterial infection”. We also thank the reviewer for stating that our work holds “clear conceptual significance for specialists in intestinal physiology and innate immunity”, and that it “has the potential to evolve into a translational framework relevant to broader biomedical contexts.” However, we believe that our work has the potential to conceptually extend beyond the intestine rather than being mostly of specialist interest. Pathogenic bacterial sensing by stem cells may promote regeneration in other stem-based epithelial tissues exposed to pathogenic bacteria from the environment, such as the lungs or skin. Moreover, we believe that the mechanism we have uncovered is likely bacterial species-independent, as we found that PGRP-LC is required in progenitors for ISC proliferation and midgut epithelial p38 activation after infection with either *P.e.* or *Ecc15*.

**Reviewer #2**

**Evidence, reproducibility and clarity**

This study investigates how oral infection in Drosophila triggers intestinal stem cell (ISC) proliferation. Previous work has shown that reactive oxygen species (ROS), produced by Duox, can lead to ISC renewal following tissue damage. More recent studies implicate the Nox pathway in stem cell activation. This manuscript proposes a novel role for a signaling pathway involving PGRP-LC/LE → Licorne (MKK3) → p38a/b in stem cell proliferation as well as a more broad gutresponse.

This manuscript addresses an important and timely question in gut immunity and stem cell biology. The proposed pathway linking bacterial detection to ISC activation is intriguing and potentially novel. However, additional experiments are critically required to validate key claims and better contextualize the findings. With these revisions, the manuscript could make a strong contribution to the field.

We thank the reviewer for the positive evaluation, and for acknowledging the potential impact of our findings by stating that “the proposed pathway linking bacterial detection to ISC activation is intriguing and potentially novel”.

Major Recommendations
1. Validation of RNAi Efficiency and Use of Mutants:
The authors must provide evidence that their RNAi constructs are functional. For instance, PGRP-LC silencing should sensitize flies to Ecc15 during systemic infection. In addition, available null mutants for PGRP-LC and PGRP-LE should be used to validate the RNAi findings and assess their effects on ISC proliferation. Similarly, the use of p38a or p38b mutants would strengthen the claim that these kinases are functionally involved.

We thank the reviewer for these suggestions. We agree that the efficacy of the *RNAi* lines for *PGRP-LC* and *PGRP-LE* need to be tested. However, we must note that one of the *PGRP-LC* *RNAis* (VDRC 101636 KK) we used in this study has been used in the following studies: Molaei et al., 2019; *Dev Cell* (Karpac lab), Zhao and Karpac, 2021; *Cell Metab*., Mazuzzo et al., 2022; *J. Neurosci.* (Royet lab) and Shin et al., 2022; *Stem Cell Reports* (Foley lab). A *PGRP-LE* *RNAi* that we used (VDRC 108199KK) was also used in Shin et al., 2022; *Stem Cell Reports*. Unfortunately, the efficacy of these lines was not reported in any of these studies, thus we could not obtain this information from these publications. Therefore, we performed qPCR to determine the efficacy of *PGRP-LC* and *PGRP-LE* *RNAi*s to deplete their targets. We depleted both *PGRP-LC* and *PGRP-LE* in ECs by expressing *RNAi*s VDRC 51968GD towards *PGRP-LC* and 108199KK towards *PGRP-LE* with *mex^ts^*. We found a 41.0% and 80.8% decrease in *PGRP-LC* and *PGRP-LE* transcript, respectively, in these midguts (S2C-D Fig.), confirming functionality of these *RNAi* constructs.

We also determined the effects of *P.e.* infection on ISC proliferation and p38 activation in *PGRP-LC* and *PGRP-LE* mutants. We found a 54.8% decrease in ISC proliferation and a decrease in midgut epithelial p38 activation in putative null *PGRP-LE^112^* mutants (kind gift from Prof. Bruno Lemaitre) after *P.e.* infection compared to control infected midguts (Fig. 1G-H). To examine ISC proliferation and midgut epithelial p38 activation in *PGRP-LC* mutants, we used two established fly strains: the null *PGRP-LC^E12^* mutant, which deletes the entire *PGRP-LC* locus, and a reported null *PGRP-LC^Δ5^* mutant (Rämet et al., 2002; *Nature*). We found that *PGRP-LC^Δ5^* showed a mild, but significant, decrease in ISC proliferation by 23.7% upon *P.e.* infection (S1A Fig.). We could not find flies homozygous for *PGRP-LC^E12^* in our lab, thus we used transheterozygotes of *PGRP-LC^E12/Δ5^* to maximally reduce *PGRP-LC* function. We found a strong 64.4% decrease in ISC proliferation and decrease in p38 activation throughout the midgut epithelium in *P.e.*-infected *PGRP-LC^E12/Δ5^* transheterozygotes (Fig. 1A-B).

Together with our *RNAi* results (Fig. 2; S2E-H’ Fig.), our data indicate that PGRP-LC and PGRP-LE are both required in progenitors to promote ISC proliferation and p38 activation throughout the midgut epithelium.

The effects on ISC proliferation in *p38a/c* and *p38b* mutants after *P.e.* infection were determined in Patel et al., 2019; *Nature commun*.. While a significant decrease in ISC proliferation was observed in *P.e.*-infected *p38b^ex9^* homozygotes, a decrease in ISC proliferation was not observed in *p38a^1^* mutants. We discuss these results in our Discussion.

2. Demonstrating the Role of Peptidoglycan:
The article imply that peptidoglycan is the ligand triggering stem cell proliferation via PGRP-LC/LE. However, this has not been directly shown. This point should be removed from the subtitle and discussed more cautiously. It remains possible that PGRP-LC/LE is activated by other microbial or host-derived ligands. The authors should test whether feeding flies DAP-type peptidoglycan or TCT (tracheal cytotoxin) stimulates stem cell proliferation. Furthermore, do heat-killed bacteria induce the same response? If not, additional signals may be required for full activation.

We thank the reviewer for raising this important point and for these suggestions. We agree that we had not shown that peptidoglycan alone can activate p38 in the midgut epithelium and ISC proliferation. Thus, we fed flies sonicated DAP-type peptidoglycan from *E. coli* to determine whether this stimulates ISC proliferation. We found that feeding DAP-type peptidoglycan to adult *Dipt-lacZ* *Drs-GFP* flies did not stimulate ISC proliferation in midguts. *Dipt-lacZ* and *Drs-GFP* are reporters for production of the antimicrobial peptides Diptericin and Drosomycin, respectively. Diptericin is produced after exposure to DAP-type peptidoglycan and Drosomycin in response to fungi and gram-positive bacteria. Using these reporters, we could determine whether our flies had fed on food containing peptidoglycan and had activated an innate immune response by producing these antimicrobial peptides. We found that midgut ECs were positive for *Diptericin* but not *Drosomycin* expression after DAP-type peptidoglycan feeding, indicating that the midgut epithelium recognised DAP-type peptidoglycan after its feeding (S3D-E” Fig.), and yet did not trigger an ISC proliferative response (Fig. 3H).

We also fed *Dipt-lacZ Drs-GFP* flies a non-virulent mutant strain of *P.e* *(ΔgacA*). We found that DAP-type peptidoglycan from this non-virulent strain was detected by the midgut epithelium (S3G-G” Fig.). Similar to peptidoglycan feeding, feeding non-virulent *P.e.* to adult flies did not stimulate ISC proliferation (Fig. 3I).

These data suggest that peptidoglycan alone does not trigger an ISC response but that additional microbial or host-derived signals may be required. For this reason, we have removed peptidoglycan from the subtitle as suggested and discussed this possibility in the Discussion.

We have also fed heat-killed *P.e.* to adult flies. To our surprise, heat-killed (95ºC) *P.e.* damaged the midgut and stimulated ISC proliferation (Fig. 3K, S3C Fig.). We confirmed that our heat-killed *P.e.* were indeed not viable. Thus, we currently believe that a *P.e.* virulence factor, possibly Monolysin, is resistant to heat treatment and is still active. Interestingly, we found that PGRP-LC was required in progenitors for ISC proliferation (81.4% decrease) after ingestion of heat-killed *P.e.* (Fig. 3K). We similarly found that PGRP-LE was required in progenitors for ISC proliferation after ingestion of heat-killed *P.e*. (S3C Fig.). These data suggest that midgut progenitors can still sense the peptidoglycan of heat-killed *P.e*. via PGRP-LC and PGRP-LE, possibly due to midgut damage caused by heat-resistant virulence factors. This could be due to the requirement of a microbial factor and a host factor produced after midgut damage, or that a breach of the midgut epithelium permits bacterial factors such as peptidoglycan to reach the basally located ISCs, triggering proliferation. We now discuss these possibilities in the Discussion.

3. Is the Imd Pathway Involved?:
The authors outline a pathway involving PGRP-LC/LE → Lic → p38 → ISC proliferation. It would be informative to determine whether this pathway also requires Imd or Relish. Notably, prior studies suggest that Relish mutants display increased ISC proliferation. This point should be addressed experimentally using mutants and RNAi.

We thank the reviewer for these interesting suggestions. Indeed, we had already started to test whether Imd is required in progenitors for p38 activation in the midgut epithelium and for ISC proliferation. We found that inhibiting *imd* with *RNAi* or by expressing a dominant-negative Imd (D30A) in progenitors reduced ISC proliferation and p38 activation in the midgut epithelium after infection (Fig. 4, S4C-D Fig.). We used the VDRC *imd RNAi* line 101834KK used in Zhai et al, 2018; *Cell Host and Microbe* (Lemaitre lab) and Krautz et al, 2020; *eLife* (Theopold lab). These data suggest a role for Imd within progenitors for ISC proliferation and p38 activation after infection. We further tested a role for Imd for ISC proliferation using an *imd* mutant (*imd^1^*). We found inconsistent proliferative responses by ISCs after *P.e* infection of *imd* mutants (S4A-B Fig.). This is different from our data using *PGRP-LC* or *PGRP-LE* mutants, which consistently show a decrease in ISC proliferation and midgut epithelial p38 activation after infection.

It is not clear, however, how Relish (NF-ϰB) would regulate p38 activity, since PGRP signalling is thought to regulate Relish independently of p38 in midgut enterocytes (Ha et al., 2009; *Nature Immunology*). Previous studies do report increased ISC proliferation in unchallenged *Relish* mutants compared to control (Buchon et al., 2009; *Cell Host and Microbe* and Wang et al., 2013) but not in infected *Rel* mutants compared to control (Buchon et al., 2009). As PGRP signalling induces Relish activity, these results are contrary to our data showing a decrease in ISC proliferation in *P.e.*-infected *PGRP-LC* and *PGRP-LE* mutant flies (above, point 1) and in *P.e.-*infected midguts expressing *RNAi*s toward *PGRP-LC* or *PGRP-LE* in progenitors (Fig. 1A, G, 2A, H and S2B Fig.). Unlike these prior studies, we found that isogenic DrosDel *Relish* mutants (*Rel^E20^*) had basal ISC proliferation levels similar to isogenic DrosDel *w^1118^* control flies (S4F Fig.). However, we also found that *P.e.*-infected *Relish* mutant midguts showed ISC proliferation levels similar to control infected midguts (S4F Fig.).

In summary, our data suggest that Imd, but not Relish, is required in the progenitors for ISC proliferation and midgut epithelial p38 activation.

Minor Recommendations
1. Page Numbering:
Please number the pages to facilitate the review process.

We apologise for this oversight. We have now added page numbers to the manuscript.

2. Clarification of Previous Literature:
Note the findings from Chakrabarti et al. (PLoS Genetics, 2014), which questioned the role of PGRP-LC in Duox activation:
"Using null mutations (Mekk1^Ur36, PGRP-LC^E12) and an RNAi construct targeting Mkk3, we found no effect of PGRP-LC on Duox transcription following P. entomophila infection, though Mkk3 and Mekk1 were required (Figure 4C)."

We thank the reviewer for pointing this out. We are aware of this study but forgot to mention it in our Introduction. We apologise for this oversight. We now include this information in the Introduction of our revised manuscript.

3. Introduction:
Please specify that Nod sense 'Gram'-negative bacterium.

We apologise for this typo. We have now edited this in our revised manuscript.

4. Typo Correction:
"RKP2" should be corrected to "RIPK2."

We apologise for this typo. We have now edited this in our revised manuscript.

5. Clarify Expression of Fold Change:
Replace ambiguous expressions such as "induced by 134%" with clearer alternatives like "increased by 134%."
"Induced by 180%" is unclear; "increased by 180%" is the correct standard.

We thank the reviewer for pointing this out. We completely agree and have edited these in our revised manuscript.

6. Use of ISC-Specific Drivers:
The current study uses esgGAL4 tubGAL80^ts Su(H)Gal80 to manipulate gene expression in ISCs and enteroblasts. To increase specificity, the use of Delta-GAL4 to target ISCs alone would strengthen the conclusions.

We thank the reviewer for this suggestion. The *esgGAL4 tubGAL80^ts^* system allows for progenitor-specific GAL4-mediated expression. However, *esgGAL4 tubGAL80^ts^ Su(H)Gal80* is an ISC-specific driver as it blocks GAL4-mediated expression in *Su(H)^+^* enteroblasts (Wang et al., 2014; *PLOS Genetics*). Data using this ISC-specific driver can be found in Fig. 7A-H, which shows that PGRP-LC as well as p38a and p38b signalling are key in ISCs to promote their proliferation and p38 activation throughout the midgut epithelium after *P.e.* infection. We did not use Dl-GAL4 in this study because we found that very few ISCs expressed GAL4 in these midguts. We found this to be the case for Dl-GAL4 stocks from two different laboratories.

We have now used the *esgGAL4 tubGAL80^ts^ Su(H)Gal80* stock to deplete *p38a* and *p38b* in ISCs and found that it decreased ISC proliferation by 59.6% and p38 activation in the midgut epithelium after *P.e.* infection (Fig. 7G-H). These data suggest that p38a and p38b signalling is key in ISCs of infected midguts for their proliferation and to promote p38 activation throughout the midgut epithelium. Using the same ISC-specific driver, we also tested if PGRP-LC is required in ISCs for their proliferation and midgut epithelial p38 activation after *P.e.* infection. *PGRP-LC* depletion specifically in ISCs resulted in a 48.3% decrease in ISC proliferation and a decrease in p38 activation in the midgut epithelium after *P.e.* infection (Figs. 7A-F). In contrast, loss of *PGRP-LC* in EBs had mild effects on both p38 activation in the midgut epithelium and ISC proliferation (Fig. 7I-J). Interestingly, loss of *p38a* and *p38b* from EBs had no effect on ISC proliferation (S6B Fig.). These data indicate that ISCs are the principal sensors of pathogenic bacteria and inducers of p38 activation throughout the midgut epithelium. The data further suggest that EBs can somewhat sense pathogenic bacteria and influence ISC proliferation, possibly independently of p38a and p38b.

7. Background on MAPK-p38 Pathway:
A brief explanation of the p38 MAPK pathway and its relevance in immune/stem cell signaling would be helpful, especially to justify the focus on Licorne.

We thank the reviewer for this suggestion. We completely agree that a brief explanation of the importance of p38 MAPK signalling in immune and stem cell signalling will be helpful to the reader. We have added this to the Introduction of our revised manuscript.

8. Figure Legends and Labels:
For clarity, instead of using "Licorne " label figures with driver and gene names (e.g., esg>Licorne) to make the experiments more accessible to readers.

We thank the reviewer for this suggestion. However, we had already included both the driver and gene/*RNAi* name in each panel. We haven’t used, for example, *esg^ts^*>Licorne, because the label often ends up obscuring data.

9. PGRP-LE Forms:
Note that the existence of a secreted/extracellular form of PGRP-LE remains unconfirmed. See: <https://reprosci.epfl.ch/>

We thank the reviewer for pointing this out. We have amended the text accordingly.

10. Materials and Methods:
This section should be more detailed. Please specify fly age, sex, , infection protocols (starvation conditions (if any)),.

We thank the reviewer for these suggestions. We had already mentioned in our Methods the age and sex of the flies used in our experiments. However, we have now added to our Methods more detailed information about our infection protocol.

11. Genotypes and Double RNAi Experiments:
The genotypes used, especially (ex double RNAi experiments on.p38a and p38b knockdown), should be fully described.

We thank the reviewer for pointing this out. We have made a list of the genotypes used in our study in S1-2 Tables. The *p38a* and *p38b* depletion does not use two independent *RNAi* lines. Two *RNAi* lines at VDRC can simultaneously target both *p38a* and *p38b* due their extensive homology. qPCR data shows that these *RNAis* can deplete both *p38a* and *p38b* transcripts. See Patel et al., 2019, Supplementary Fig. 1j.

**Significance**

This manuscript addresses an important and timely question in gut immunity and stem cell biology. The proposed pathway linking bacterial detection to ISC activation is intriguing and potentially novel. However, additional experiments are critically required to validate key claims and better contextualize the findings. With these revisions, the manuscript could make a strong contribution to the field.

We thank the reviewer for the positive evaluation of our work and for stating that “the proposed pathway linking bacterial detection to ISC activation is intriguing and potentially novel”. We believe that our revisions have allowed our work to make a strong contribution to the field and hope that the reviewer agrees.

Reviewer #3 (Evidence, reproducibility and clarity (Required)):

Uttekar et al. present evidence of direct sensing of bacterial pathogens by the progenitor population of the adult Drosophila midgut. They delineate the molecular pathway that allows this detection and links it to a proliferative response, identifying DAP-type proteoglycan receptors PGRP-LC and LE as receptors, and the MKK3 > p38 pathway as the intracellular transducer that leads to proliferation. This is a very interesting observation that changes considerably the perspective on how the epithelial response to bacterial infections is organised. So far, the view of infection defence has been dominated by a strong separation of roles, where terminally differentiated cells (mostly enterocytes) did all the sensing (directly or through damage), mounted an anti-microbial response, and stimulated proliferation of the progenitor population through secreted factors. Progenitors were there just to provide new cells. The results presented here dynamite this view, and open important new questions about the extent to which progenitors contribute to the infection response. For instance, it is clear that ISCs not only participate in sensing, but also function to stimulate p38 activation in nearby ECs, which can autonomously both detect the pathogens and activate p38 through another pathway. The authors make a compelling case with their experimental approach and the data, but the paper is a bit thin on mechanism.

Overall, the phenomenology is of sufficient interest to be disseminated widely and soon. On the other hand, the authors have not made a full attempt at dissecting the mechanism with the same level of detail that one could expect in Drosophila. While I do not think this is necessarily a problem, I have a few caveats with the characterisation of the phenomenology.

We thank the reviewer for the positive evaluation and for stating that our “very interesting observation changes considerably the perspective on how the epithelial response to bacterial infections is organised”. Further, we thank the reviewer for highlighting that our work should be “disseminated widely and soon”.

Main scientific comments:

1 __ Fluorescence of phospho-p38.

The authors claim that according to the results in Figure 1A-D, the levels of phospho-p38 after P.e. infection in ISCs/EBs are higher than in non-treated conditions. I do not doubt the results, but I think several aspects of their reporting needs clarification:

- Imaging conditions: making direct comparisons of fluorescence intensity in images originating from different batches of immuno-staining requires, as the authors declare, staining and imaging conditions that minimise variation. However, even with all experimental procedures minimising variation, a normalisation approach is required (i.e. using one normalisation window with no staining per image as within-image normalisation factor). It does not seem that this has been described in methods. Is there a solid reason whereby this is not necessary?

We agree with the reviewer that a normalisation approach is required. We found that the background levels were similar between samples within an experiment, thus we did not report background subtracted measurements. The background (raw integrated density/area) was determined for each sample from an area on the image without any tissue. We have now subtracted the background from the fluorescence intensity (raw integrated density/area) from the tissue. We have now also described this normalisation in the Methods and have reported these normalised measurements in our revised manuscript.

- Segmentation approach: authors have made projections in the apical-basal axis and measured the intensity in the ROIs resulting from the outline of signal from GFP expressed in progenitors. No reference is made to how the GFP signal is thresholded. Moreover, midgut progenitors are pyramidal cells with a larger basal than apical perimeter. This leads to an overlap with some of the neighbouring ECs when projected along their apical-basal axis. This is quite obvious in Figure 1B-B', where the GFP outlines include slices of high signal that belong to the neighbour ECs. While by eye it is clear that these progenitors have higher levels of signal than the untreated ones from figure 1A-A' anyway, the extent of this difference may have been greatly overestimated if this overlap has been systematically included across the dataset. The presence of this overlap is also so obvious that may cast strong doubts in the reader. One solution would be to measure the signal individually in each of the Z-slices projected (which can be done automatically in ImageJ), and show only one slice in Figure 1A-B.

We thank the reviewer for pointing these issues out.

With our new analyses of the progenitor signal, thresholding was not required.

We agree that using stacks of midgut progenitors for p-p38 measurements likely introduces some signal from neighbouring ECs, and this could overestimate the signal within progenitors. We have reanalysed our data by determining the fluorescent intensity (raw integrated density) per progenitor from each of the Z-slices. The mean raw integrated density for each progenitor was then divided by the mean area of each progenitor to obtain the normalised p-p38 fluorescence intensity per progenitor. We plotted the number of progenitors per normalised fluorescence intensity (histogram). Moreover, we have now presented only one slice in our Figures and have included a description of our progenitor signal quantitation in our revised Methods. We would like to thank the reviewer for this suggestion as our re-analysis has improved our data.

- Infection with P.e. induces a rapid regenerative response (whose characterization has been in part the contribution of some of the authors of this manuscript). I wonder whether the acceleration of proliferation induces a situation where progenitor cells differentiate terminally faster than the GFP that labels them degrades. This would lead to a situation where they are considering the signal of phospho-p38 in early ECs that are still labelled by GFP molecules that they are no longer producing, but are responding to Pe infection as ECs, not progenitors. If the authors could comment on whether GFP perdurance was apparent in their images and if so, on what precautions have they taken to eliminate from their phospho-p38 dataset cells that were polyploid and/or displaying enlarged cytoplasm, it would make the results more convincing.

We thank the reviewer for pointing this out. It is true that at times there are early ECs that are labelled with GFP due to rapid ISC proliferation. To exclude these cells from our p-p38 quantitation, we have used a macro that we developed using images of esg>GFP^+^ progenitors from homeostatic midguts to identify cells with a progenitor nuclear size. However, we had not applied this analysis to our images and had outlined all GFP-positive cells. Thus, we have now removed outlines around progenitors with nuclei larger than esg>GFP^+^ progenitors in homeostatic midguts. We describe this analysis in our Methods.

We will also make our macro available to download from the journal and our lab website for other researchers to use with their research.

These comments apply as well to Figures 2A-C and 3, 4, 5, and 6 (panels B-K).

- More generally, the specificity of the anti-phosphorylated-human p38 does not seem to have been established using RNAi nor mutants. Is this demonstrated in an earlier work? I could not find this information in ref. 41. I am not suggesting to do additional experiments necessarily, but any information the authors have on this would be quite helpful to include (maybe in methods).

We thank the reviewer for this question. It has been shown that this antibody is specific for phosphorylated p38a and p38b. This antibody was used in Patel et al., 2019; *Nat Commun* to examine p38 activation in midgut enterocytes after infection. Simultaneously depleting both *p38a* and *p38b* with *RNAi* in ECs in *P.e.*-infected midguts reduced p-p38 levels in the midgut epithelium (see Patel et al., 2019 Fig. 1a-b, Suppl. Fig. 1a-b). We have now stated in our Methods that the specificity of the p-p38 antibody was tested in Patel et al., 2019. Furthermore, the depletion of p38b alone in progenitors results in a decrease in p-p38 levels in midgut progenitors by 87.8% after *P.e.* infection compared to control infected progenitors, reconfirming the specificity of the antibody (see Fig. 3B).

2 __ Directly addressing the sensing of DAP-PG

The authors delineate a pathway whereby DAP-type proteoglycans stimulate PGRP-LC and PGRP-LE, which in turn activates the cascade Licorne > p38a/b > proliferation/non-autonomous signal propagation. This suggests that the sensing of bacteria is independent of damage and also very sensitive to thresholds (as removing either PGRP strongly suppresses the response). I am surprised that the authors have not attempted to feed the flies DAP-PG to ensure that the response can be triggered in isolation from damage locally or elsewhere. I am not suggesting every single experiment repeated with feeding instead of infection - but some key experiments showing that there is a similar response in the wild type and this is suppressed with e.g. p38a/b RNAi, would strengthen the proposed phenomenology.

We thank the reviewer for this suggestion. We had indeed not fed DAP-type peptidoglycan to adult flies. As discussed above (Reviewer 2 point 2), we fed DAP-type peptidoglycan to adults but found that it alone does not stimulate ISC proliferation. For this reason, we are unable to also test if depleting *p38a* and *p38b* from progenitors inhibits ISC proliferation after DAP-type peptidoglycan feeding.

Minor scientific comments:

3 __ Non cell-autonomous propagation of p38 signalling

Figure 2 shows that overexpression of Licorne induces increased phospho-p38 not only in progenitors, but in some neighbouring ECs. This is not a problem in itself, as the authors show already in Fig 1G and S1D-G that you need activation of the pathway in progenitor cells for ECs to become responsive of it. One can assume that some signal emanates from progenitors to stimulate the pathway in ECs. However, this would suggest some correlation between the levels of p-p38 in ECs and their neighbouring progenitors. Is this the case? The authors could verify this with the existing data.

We thank the reviewer for this question. We analysed our existing data to determine if there is a correlation between levels of p-p38 in progenitors and their neighbouring ECs. To do this, we determined and plotted the fluorescence intensity in the progenitor and its adjacent ECs to identify a linear relationship between the two. We performed this analysis in both control midguts and in midguts with progenitors overexpressing *licorne*. We also calculated the Pearson correlation coefficient to demonstrate the strength of this relationship. Our analysis shows a strong linear relation between p38 activation in progenitors and their neighbouring EC in both control midguts (*Pearsons* *r* =0.68) and in midguts overexpressing *licorne* in progenitors (Pearsons *r* =0.60). These data indicate that p38 activation by *licorne* overexpression in progenitors results in linear p38 activation in both progenitors and ECs. Furthermore, the strong correlation found between progenitors and ECs in control midguts suggests that basal p38 activity in progenitors regulates p38 activity in ECs.

4 __ Epistasis

The pathway delineation is based on what is known about the molecular function of the proteins and the p-p38 antibody. This is OK, but maybe the authors could consider one epistasis experiment between PGPRs and Licorne or an activated form of p38, to have genetic evidence of the episatsis.

We thank the reviewer for this suggestion. Expressing *PGRP-LC* (or -*LE*) *RNAi* in progenitors blocks ISC proliferation after *P.e.* infection. Thus, we tested whether co-expression of *licorne* and *PGRP-LC* (or -*LE*) *RNAi* could rescue ISC proliferation during infection. Unfortunately, progenitors were not viable after simultaneously depleting *PGRP-LC* with *RNAi* and overexpressing *licorne*, thus we were unable to perform this experiment. See our quantitation of *esg^+^* progenitors below.

5 __ Mechanistic hints

I do not expect the authors to fully characterise aspects of the mechanism that are clearly missing, like how increased p38 activation leads to proliferation or how progenitors influence p38 activation in neighbouring ECs. However, the authors could analyse existing data to offer some possibilities, even if it is just in the discussion. For instance, the authors could re-analyse existing data of the cell-type specific transcriptional response to Pseudomonas entomophila infection (Dutta et al., 2015) (and existing data from other papers) to make suggestions about potential diffusible signals modulating activation of p38 in ECs or proliferative genes potentially targeted by p38 signalling.

We thank the reviewer for this suggestion. We have, based on the literature, suggested in our revised Discussion possible diffusible signals from progenitors that may activate p38 in ECs. We likewise suggest potential factors that could be regulated by p38 signalling to promote ISC proliferation.

Language/presentation comments:

Intro:

"Thus, it remains unknown whether midgut cell types, particularly ISCs, can recognise peptidoglycan and contribute directly to regeneration." I would change "recognise peptidoglycan and contribute directly" for "recognise peptidoglycan DIRECTLY TO contribute" as they would contribute _directly_ to regeneration through non-autonomously induced proliferation (arguably they do all the regenerative work as they are the only indefinitely proliferative cell type); the question is whether they do the _sensing_ directly.

We thank the reviewer for this suggestion. We have now edited this text to improve clarity.

"Nod1 senses -negative bacteria" -- I think a 'gram' is missing before '-negative'.

We apologise for this oversight, and we have edited this in our revised manuscript.

"which does not occur upon infection with pathogenic bacteria." I am not sure what is 'Which' referring to. Is it to the regeneration, to the requirement of Nod2 for it or to the ISC and crypt cell depletion resulting from doxorubicin treatment? The next sentence suggests that it is the ISC/crypt cell depletion, but then there is some redundancy between the two.

Indeed, this refers to ISC/crypt cell depletion. We have edited this text in our Introduction.

Results:

"Further, depletion of p38b alone in progenitors blocked p38 activation in the entire midgut epithelium by 62.9% (Figure 1G)." Where it says 'blocked' it should be 'reduced'.

We thank the reviewer for this helpful suggestion. We completely agree with the reviewer and have modified when needed “blocked” to “reduced” or “decreased” throughout the manuscript.

Figure 1G: why is one transgene denominated [RNAi] but the other is [antisense]? Is there a fundamental difference in the constructs that needs to be highlighted like this?

We thank the reviewer for this question. This stock is designated as *p38b^antisense^* in Adachi-Yamada et al., 1999. The *UAS-p38b^antisense^* construct was made by inserting the full length p38b cDNA but inverted into pUAST. This suggests that the antisense strand is expressed by GAL4. Since the antisense strand likely doesn’t form a hairpin (double stranded RNA) it may block translation of *p38b* mRNA rather than participating in *RNAi*-mediated knockdown of *p38b* mRNA.

Figure S2 title repeats "in progenitors".

We have edited the title for S2 Figure.

Figure legends in general refer to "control midguts" but it is not clear what is the genotype of such controls. The methods section refers to a KK control line and a UAS-mCherry[RNAi] control line, but it is not specified which one is use as controls in which experiment. Similarly, it is not clear what the controls for UAS-cDNA experiments (e.g. UAS-licorne) are.

We thank the reviewer for these questions. We have made a list of genotypes (S1-2 Tables) used for each experiment as requested by Reviewer 2. The KK control line was used when a VDRC KK line was used for an experiment; a *UAS-mCherry* *RNAi*, when a TRiP *RNAi* was used for an experiment. Further, *w^1118^* was used as a control for UAS-cDNA experiments.

In swarm plots, bars and whiskers are not described in every case. By magnitude, they look SD, but SEM might be more appropriate in some cases (e.g. Fig S1A).

We apologise for this oversight. These were the mean and 95% confidence interval for plots in our previous figures. However, as stated above in response to Reviewer 1 point 2, we have used descriptive statistics (standard deviation) in the revised manuscript for plots describing data per midgut. For plots describing mitoses per midgut, the bar represents the mean and the whiskers, 95% confidence intervals. This information is now described in the revised Figure legends.

In section "PGRP-LC/LE-MKK3-p38 activity in progenitors activates p38 in the midgut epithelium after pathogenic bacterial infection", the authors write: "Thus, we asked whether it was increased ISC proliferation due to increased PGRP-LC/LE- MKK3-p38 signalling in progenitors that stimulated p38 activation throughout the midgut epithelium or whether it was specifically due to p38 signalling." I had to re-read this to work out that the authors suggest a potential 'pathway' such as: [ PGPR-LC/LE > Licorne > proliferation > p38 activation ] or rather [ PGPR-LC/LE > Licorne > p38 activation ]. May I suggest that the authors consider editing this paragraph for clarity.

We thank the reviewer for pointing this out. We have edited this paragraph to improve clarity.

Discussion:

The discussion is very repetitive with results (do we really need to read again the percentages with one decimal?) and does not really make an integrated attempt to indicate how the change in perspective impacts our understanding of epithelial immunity/regeneration. The few pointers that are offered could be written more succinctly.

We thank the reviewer for this helpful suggestion. We agree that the Discussion could include more discussion of how the work changes our understanding of midgut epithelial immunity and regeneration. We have amended our Discussion in our revised manuscript.

General:

What is exactly the 'regenerative niche' for the authors? Which cells and/or other anatomical entity?

We thank the reviewer for this question. We had defined the regenerative niche (or microenvironment) as all the midgut cell types that influence ISC behavior, such as proliferation, after damage. These include midgut epithelial cells such as EBs, ECs and EEs, the visceral muscle and trachea. This is described in the Introduction on page 2. However, since there is not a subset of specialist “niche” cells that specifically regulate ISC proliferation during midgut regeneration, we have now used the term “regenerative microenvironment”.

**Referee cross-commenting**

All comments by the other reviewers seem sensible to me. I think evaluating the involvement of the Imd pathway, while it would make the paper certainly stronger, may be construed as slightly out of scope.

Thank you for commenting on the other reviewer reports. Out of interest, we have determined whether Imd is required in progenitors for ISC proliferation and midgut epithelial p38 activation after pathogenic infection. Our data suggest Imd is necessary in progenitors for p38 activation throughout the midgut epithelium and ISC proliferation after *P.e*. infection. See Reviewer 2, point 3.

Reviewer #3 (Significance (Required)):

Uttekar et al. present evidence of direct sensing of bacterial pathogens by the progenitor population of the adult Drosophila midgut. They delineate the molecular pathway that allows this detection and links it to a proliferative response, identifying DAP-type proteoglycan receptors PGRP-LC and LE as receptors, and the MKK3 > p38 pathway as the intracellular transducer that leads to proliferation. This is a very interesting observation that changes considerably the perspective on how the epithelial response to bacterial infections is organised. So far, the view of infection defence has been dominated by a strong separation of roles, where terminally differentiated cells (mostly enterocytes) did all the sensing (directly or through damage), mounted an anti-microbial response, and stimulated proliferation of the progenitor population through secreted factors. Progenitors were there just to provide new cells. The results presented here dynamite this view, and open important new questions about the extent to which progenitors contribute to the infection response. For instance, it is clear that ISCs not only participate in sensing, but also function to stimulate p38 activation in nearby ECs, which can autonomously both detect the pathogens and activate p38 through another pathway. The authors make a compelling case with their experimental approach and the data, but the paper is a bit thin on mechanism.

Overall, the phenomenology is of sufficient interest to be disseminated widely and soon. On the other hand, the authors have not made a full attempt at dissecting the mechanism with the same level of detail that one could expect in Drosophila. While I do not think this is necessarily a problem, I have a few caveats with the characterisation of the phenomenology.

We thank the reviewer for the very helpful and constructive review that has further improved our work.
