# Supplementary material for: Recognition of pathogenic bacteria by intestinal stem cells promotes adult Drosophila midgut regeneration
Source: PLoS Biol. 2026 Aug 25;24(8):e3003951. doi: 10.1371/journal.pbio.3003951 (PMC13541249; doi:10.1371/journal.pbio.3003951)
Supplement: Supplementary file 6 [file pbio.3003951.s016.docx]

We would like to thank the reviewers for their helpful suggestions, which have clarified and strengthened our manuscript. We have carefully addressed each comment by adding clarifications throughout the manuscript.

Rev. 1: François Leulier - note that this reviewer has signed the review.
The revised version of the MS is addressing adequately my initial comments. I believe the MS is now more solid and make a more compelling case supporting the author's model.

We would like to thank the reviewer for stating that our work is “more solid and make a more compelling case supporting” our model.

Rev. 2:
The revised version has been substantially improved. The results are intriguing, as they reveal a direct effect of pathogenic bacteria on stem cell proliferation mediated through a PGRP-LC/PGRP-LE-Imd-p38 signaling pathway. Another unexpected finding is that peptidoglycan, the microbial elicitor sensed by these receptors, is not sufficient on its own to reproduce this effect, suggesting that both tissue damage and peptidoglycan are required to drive the stem cell response. Overall, the study is interesting and thought-provoking, although future additional work might be required to fully substantiate these conclusions. Several points, however, still need to be addressed.

We thank the reviewer for stating that our results are “intriguing” and that our study is “interesting and thought-provoking”. We look forward to investigating in the future how tissue damage and peptidoglycan together activate epithelial p38 and ISC proliferation.

Major points
-'null PGRP-LC[E12] mutants were not viable as homozygotes on our medium'. This is very strange because PGRP-LC[E12] is a viable mutation.

We thank the reviewer for this question. Unfortunately, we do not understand why the *PGRP-LC^E12^* homozygous mutants are not viable on our medium. Nevertheless, there is a strong defect in ISC proliferation in *P.e.*-infected *PGRP-LC^E12^/^Δ5^* transheterozygotes. We received two *PGRP-LC^E12^* stocks over balancers from Prof. Bruno Lemaitre, an expert on *Drosophila* immunity and the Imd pathway. This suggests that *PGRP-LC^E12^* mutants don't grow well as homozygotes. In contrast, we received *PGRP-LE^112^* as a homozygous viable stock from the same lab, and these thrive on our medium. It is possible that there is an interaction between the culture conditions and *PGRP-LC* mutants that is beneficial for their growth as homozygotes but is absent from our culture conditions. In any case, we would like to emphasise that our transheterozygote analysis helps minimise background effects on epithelial p38 activation or ISC proliferation.

- Implication of Imd:
The authors report no effect of the imd[1] mutation on ISC proliferation (Figure S4A), in contrast to the phenotype observed upon stem cell-specific RNAi knockdown. This discrepancy is surprising and warrants further clarification. One possible explanation is the existence of compensatory mechanisms when Imd is impaired in all cells, but not when it is specifically depleted in stem cells. Alternatively, the imd[1] allele, a strong hypomorph, may not fully abolish Imd function in the context of p38 activation. This issue could be addressed by testing a bona fide null allele of imd, such as imd[shadok] (generated by the Ferrandon laboratory). In addition, Minos insertions in the imd locus that are likely null alleles could also be considered to strengthen the analysis.

We thank the reviewer for this question, and we too found it puzzling that the *imd^1^* mutant did not consistently show a decrease in ISC proliferation.

We agree that it is possible that compensatory p38 activation occurs within midguts of *imd^1^* mutants. Such compensatory p38 activation could promote ISC proliferation in *imd^1^* mutants, counteracting the decrease in ISC proliferation that would otherwise be expected. We have now further elaborated on this in our Discussion (see page 12). Nevertheless, we have shown that inhibiting Imd signalling specifically in progenitors reduces both ISC proliferation and epithelial p38 activation after pathogenic bacterial infection, which is key for this study.

Minor points:
- Introduction
'This increased ROS production eliminates invading pathogenic bacteria from the lumen (33, 41)' This statement has been recently contradicted in a reproducibility experiment. This should be mentioned (see Westlake et al Elife 2026 <https://doi.org/10.7554/eLife.108404.1)>.

We thank the reviewer for pointing out this study. We have now cited and included this study in our discussion in our Introduction and Discussion.

- Drosomysin should be Drosomycin

We apologise for this typo. Since we have removed the *Drs-GFP* data (see below), we have now removed the mention of Drosomycin from the manuscript.

- 'This indicates that after adult flies consumed food containing DAP-type peptidoglycan or P.e., midgut ECs launched an innate immune response by producing AMPs.' This statement is not new and could be back up by references (ex among others Boscon-Drayon 2012; Neyen 2012)

We thank the reviewer for pointing out this oversight. We are fully aware that this is not a new observation and apologise for not citing these studies. We have clarified in the text that this is not a new finding and included these references.

- Use of Drs-GFP in the midgut: There is no evidence for a role of Toll pathway in the midgut to regulate AMPs and Drs is not induced in the gut (Buchon 2009). Thus, this experiment should be removed. The authors do not need to discuss the Toll pathway since the focus is on PGRP-LC/LE.

We used *Drs-GFP* as a negative control to show that DAP-type peptidoglycan from *E. coli*, *P.e.* or *P.e. ΔgacA* do not activate the Toll pathway. We have now removed these data from our manuscript.

- I could not figure what is the meaning of 5d +-18h on the figure panels? Are they monitoring stem cell proliferation at 5 days post infection or at 18h post infection using 5 days old flies? This is important to clarify.

We apologise for our lack of clarity. When 5d ± 18h is indicated, it means that we have turned on our *UAS*-transgene for 5 days at 29°C using conditional GAL4 genetics and then treated (or not treated) flies for 18h (for example, *P.e.* infection). Flies are between 5-10 days old at the start of each experiment. We have clarified this further in the Results for the first experiment mentioned. See page 5.

- Mode of infection: Is it continuous feeding or one shot? Those types of information should appear in the result section.

The flies have been continuously exposed to pathogenic bacteria for 18 hours. We clarified this now within the text and Methods. However, we cannot say if the flies have continuously fed on the bacteria. We have described at first mention that *P.e.* infection is continuous (page 5) and in the Methods (page 15).

-Two points I raised here but that were not mentioned in my first review came to my mind. I would understand if the authors do not want to address them but I still consider them as important.

We agree that these two points are important to determine, particularly the analysis of *PGRP-LC* and *PGRP-LE* double mutants. However, we feel that these experiments are currently not essential to support the main conclusions of the manuscript.

- Most observations are performed at a single time point (4 days or 18h). It would greatly strengthen the study to include a kinetic analysis of stem cell proliferation (e.g., using PH3 staining) to determine at which stage of epithelial renewal the Imd pathway is required. For instance, Liu et al. (Cell Reports) performed a time-course analysis at 16 h, 48 h, and 72 h. Such an approach would help clarify whether the Imd-dependent effect represents an early or late response.

Flies were dissected after 18 hours of treatment (for example, *P.e.* infection) unless otherwise stated. We dissected flies for one experiment 12 hours post-infection (Fig 2I-J) and found a decrease in progenitor and epithelial p38 activation in midguts with progenitors depleted for *PGRP-LE*. This further suggests a decrease in ISC proliferation in these midguts 12 hours post-infection. In our lab, we find ISC proliferation significantly increases about 6 hours post-infection (CFU= 7.0x10^9^ bacteria/ml), thus this suggests that loss of PGRP signalling causes a proliferation defect between 6-12 hours. Therefore, we believe that the PGRP-Imd-dependent effect is an early response. Nevertheless, we hope to perform a time course in the future to pinpoint the exact response period.

- The observation that both PGRP-LE and PGRP-LC individually have strong effects is somewhat puzzling, as these receptors are generally thought to function independently. It would therefore be informative to analyze double mutants (PGRP-LE; PGRP-LC) to assess potential redundancy or additive effects between these pathways.

We agree entirely with the reviewer that both the loss of PGRP-LC and PGRP-LE have similar effects although we do find often that the loss of PGRP-LC often exhibits stronger effects than PGRP-LE loss. Our data suggest that both of these proteins function together in progenitors in the same biological process rather than independently, which we find fascinating. We are planning to generate double mutants for *PGRP-LE; PGRP-LC* in the future to determine whether they work together or independently.
