# Supplementary material for: Recognition of pathogenic bacteria by intestinal stem cells promotes adult Drosophila midgut regeneration
Source: PLoS Biol. 2026 Aug 25;24(8):e3003951. doi: 10.1371/journal.pbio.3003951 (PMC13541249; doi:10.1371/journal.pbio.3003951)
Supplement: Peer review history

# Author comment: Author response to Decision Letter 0

**DOI:** 10.1371/journal.pbio.3003951.r001

23 Sep 2025

Attachment Submitted filename: 158276_1_rebuttal_3518258_t2xlq6.pdf

# Editor report: Decision Letter 0

**DOI:** 10.1371/journal.pbio.3003951.r002

**Authors:** Ines Alvarez-Garcia

9 Oct 2025

Dear Dr Patel,

Thank you for submitting via Review Commons your manuscript entitled "Recognition of pathogenic bacteria by intestinal progenitors promotes adult Drosophila midgut regeneration via PGRP-MKK3-p38 signalling" for consideration as a Research Article by PLOS Biology.

Your manuscript and associated reviews and revision plan have now been evaluated by the PLOS Biology editorial staff as well as by an academic editor with relevant expertise and I am writing to let you know that we would like to consider a revision.

However, before we send you the decision, we need you to complete your submission by providing the metadata that is required. To this end, please login to Editorial Manager where you will find the paper in the 'Submissions Needing Revisions' folder on your homepage. Please click 'Revise Submission' from the Action Links and complete all additional questions in the submission questionnaire.

Once your full submission is complete, your paper will undergo a series of checks. After your manuscript has passed the checks. we will send you the decision. To provide the metadata for your submission, please Login to Editorial Manager (https://www.editorialmanager.com/pbiology) within two working days, i.e. by Oct 13 2025 11:59PM.

During the process of completing your manuscript submission, you will be invited to opt-in to posting your pre-review manuscript as a bioRxiv preprint. Visit http://journals.plos.org/plosbiology/s/preprints for full details. If you consent to posting your current manuscript as a preprint, please upload a single Preprint PDF.

Feel free to email us at plosbiology@plos.org if you have any queries relating to your submission.

Kind regards,

Ines

--

Ines Alvarez-Garcia, PhD

Senior Editor

PLOS Biology

ialvarez-garcia@plos.org

# Author comment: Author response to Decision Letter 1

**DOI:** 10.1371/journal.pbio.3003951.r003

14 Oct 2025

Attachment Submitted filename: 158276_1_rebuttal_3518258_t2xlq6_auresp_1.pdf

# Editor report: Decision Letter 1

**DOI:** 10.1371/journal.pbio.3003951.r004

**Authors:** Ines Alvarez-Garcia

16 Oct 2025

Dear Dr Patel,

Thank you for submitting the requested metadata for your Review Commons manuscript entitled "Recognition of pathogenic bacteria by intestinal progenitors promotes adult Drosophila midgut regeneration via PGRP-MKK3-p38 signalling" and currently under consideration at PLOS Biology.

As I mentioned, we have evaluated the associated reviews and revision plan, and we would like to invite you to revise the work to thoroughly address the reviewers' reports according to the plan you suggest.

Given the extent of revision needed, we cannot make a decision about publication until we have seen the revised manuscript and your response to the reviewers' comments. Your revised manuscript is likely to be sent for further evaluation by all or a subset of the reviewers.

In addition to these revisions, you will need to complete some formatting changes, which you will receive in a follow up email. A member of our team will be in touch with a set of requests shortly.

We expect to receive your revised manuscript within 3 months. Please email us (plosbiology@plos.org) if you have any questions or concerns, or would like to request an extension.

At this stage, your manuscript remains formally under active consideration at our journal; please notify us by email if you do not intend to submit a revision so that we may withdraw it.

**IMPORTANT - SUBMITTING YOUR REVISION**

Your revisions should address the specific points made by each reviewer. Please submit the following files along with your revised manuscript:

1. A 'Response to Reviewers' file - this should detail your responses to the editorial requests, present a point-by-point response to all of the reviewers' comments, and indicate the changes made to the manuscript.

*NOTE: In your point-by-point response to the reviewers, please provide the full context of each review. Do not selectively quote paragraphs or sentences to reply to. The entire set of reviewer comments should be present in full and each specific point should be responded to individually, point by point.

You should also cite any additional relevant literature that has been published since the original submission and mention any additional citations in your response.

2. In addition to a clean copy of the manuscript, please also upload a 'track-changes' version of your manuscript that specifies the edits made. This should be uploaded as a "Revised Article with Changes Highlighted" file type.

3. Resubmission Checklist

When you are ready to resubmit your revised manuscript, please refer to this resubmission checklist: https://plos.io/Biology_Checklist

To submit a revised version of your manuscript, please go to https://www.editorialmanager.com/pbiology/ and log in as an Author. Click the link labelled 'Submissions Needing Revision' where you will find your submission record.

Please make sure to read the following important policies and guidelines while preparing your revision and fulfil the editorial requests:

a) *PLOS Data Policy*

Please note that as a condition of publication PLOS' data policy (http://journals.plos.org/plosbiology/s/data-availability) requires that you make available all data used to draw the conclusions arrived at in your manuscript. If you have not already done so, you must include any data used in your manuscript either in appropriate repositories, within the body of the manuscript, or as supporting information (N.B. this includes any numerical values that were used to generate graphs, histograms etc.). Please also indicate in each figure legend where the data can be found. For an example see here: http://www.plosbiology.org/article/info%3Adoi%2F10.1371%2Fjournal.pbio.1001908#s5

b) *Published Peer Review*

Please note while forming your response, if your article is accepted, you may have the opportunity to make the peer review history publicly available. The record will include editor decision letters (with reviews) and your responses to reviewer comments. If eligible, we will contact you to opt in or out. Please see here for more details:

https://blogs.plos.org/plos/2019/05/plos-journals-now-open-for-published-peer-review/

c) *Blot and Gel Data Policy*

Please provide the original, uncropped and minimally adjusted images supporting all blot and gel results reported in an article's figures or Supporting Information files. We will require these files before a manuscript can be accepted so please prepare them now, if you have not already uploaded them. Please carefully read our guidelines for how to prepare and upload this data: https://journals.plos.org/plosbiology/s/figures#loc-blot-and-gel-reporting-requirements

d) *Blurb*

Please also provide a blurb which (if accepted) will be included in our weekly and monthly Electronic Table of Contents, sent out to readers of PLOS Biology, and may be used to promote your article in social media. The blurb should be about 30-40 words long and is subject to editorial changes. It should, without exaggeration, entice people to read your manuscript. It should not be redundant with the title and should not contain acronyms or abbreviations. For examples, view our author guidelines: https://journals.plos.org/plosbiology/s/revising-your-manuscript#loc-blurb

e) *Protocols deposition*

To enhance the reproducibility of your results, we recommend that if applicable you deposit your laboratory protocols in protocols.io, where a protocol can be assigned its own identifier (DOI) such that it can be cited independently in the future. For instructions see: https://journals.plos.org/plosbiology/s/submission-guidelines#loc-materials-and-methods

Thank you again for your submission to our journal. We hope that our editorial process has been constructive thus far, and we welcome your feedback at any time. Please don't hesitate to contact us if you have any questions or comments.

Sincerely,

Ines

--

Ines Alvarez-Garcia, PhD

Senior Editor

PLOS Biology

ialvarez-garcia@plos.org

# Author comment: Author response to Decision Letter 2

**DOI:** 10.1371/journal.pbio.3003951.r005

6 May 2026

Attachment Submitted filename: response to reviewers_Uttekar.docx

# Peer review: Decision Letter 2

**DOI:** 10.1371/journal.pbio.3003951.r006

**Authors:** Ines Alvarez-Garcia

6 Jul 2026

Dear Dr Patel,

Thank you for your patience while we considered your revised manuscript entitled "Recognition of pathogenic bacteria by intestinal stem cells promotes adult Drosophila midgut regeneration" for publication as a Research Article at PLOS Biology. This revised version of your manuscript has been evaluated by the PLOS Biology editors, the Academic Editor and two of the original reviewers.

Based on the reviews, we are likely to accept this manuscript for publication, provided you satisfactorily address the remaining points raised by Reviewer 2, although please note that we won't make the analysis of the double mutants a requirement for publication. Please also make sure to address the data and other policy-related requests stated below my signature.

As you address these items, please take this last chance to review your reference list to ensure that it is complete and correct. If you have cited papers that have been retracted, please include the rationale for doing so in the manuscript text, or remove these references and replace them with relevant current references. Any changes to the reference list should be mentioned in the cover letter that accompanies your revised manuscript.

In addition to these revisions, you may need to complete some formatting changes, which you will receive in a follow up email. A member of our team will be in touch with a set of requests shortly. If you do not receive a separate email within a few days, please assume that checks have been completed, and no additional changes are required.

We expect to receive your revised manuscript within two weeks.

To submit your revision, please go to https://www.editorialmanager.com/pbiology/ and log in as an Author. Click the link labelled 'Submissions Needing Revision' to find your submission record. Your revised submission must include the following:

- a cover letter that should detail your responses to any editorial requests, if applicable, and whether changes have been made to the reference list

- a Response to Reviewers file that provides a detailed response to the reviewers' comments (if applicable, if not applicable please do not delete your existing 'Response to Reviewers' file.)

- a track-changes file indicating any changes that you have made to the manuscript.

NOTE: If Supporting Information files are included with your article, note that these are not copyedited and will be published as they are submitted. Please ensure that these files are legible and of high quality (at least 300 dpi) in an easily accessible file format. For this reason, please be aware that any references listed in an SI file will not be indexed. For more information, see our Supporting Information guidelines:

https://journals.plos.org/plosbiology/s/supporting-information

*Published Peer Review History*

Please note that you may have the opportunity to make the peer review history publicly available. The record will include editor decision letters (with reviews) and your responses to reviewer comments. If eligible, we will contact you to opt in or out. Please see here for more details:

https://plos.org/published-peer-review-history/

*Press*

Should you, your institution's press office or the journal office choose to press release your paper, please ensure you have opted out of Early Article Posting on the submission form. We ask that you notify us as soon as possible if you or your institution is planning to press release the article.

*Protocols deposition*

To enhance the reproducibility of your results, we recommend that if applicable you deposit your laboratory protocols in protocols.io, where a protocol can be assigned its own identifier (DOI) such that it can be cited independently in the future. Additionally, PLOS ONE offers an option for publishing peer-reviewed Lab Protocol articles, which describe protocols hosted on protocols.io. Read more information on sharing protocols at https://plos.org/protocols?utm_medium=editorial-email&utm_source=authorletters&utm_campaign=protocols

Please do not hesitate to contact me should you have any questions.

Sincerely,

Ines

--

Ines Alvarez-Garcia, PhD

Senior Editor

PLOS Biology

ialvarez-garcia@plos.org

------------------------------------------------------------------------

DATA POLICY:

Many thanks for providing all the data underlying the graphs shown in the figures. Please ensure that:

1) The data file is referred to in the manuscript using the following format verbatim: S1 Data, and that the file is saved using exactly the following convention: S1_Data.xlsx (using an underscore).

2) Please indicate in each corresponding figure legend where the underlying data can be found, and ensure your supplemental data file/s has a legend.

------------------------------------------------------------------------

CODE POLICY

Per journal policy, if you have generated any custom code during the course of this investigation, please make it available without restrictions. Please ensure that the code is sufficiently well documented and reusable, and that your Data Statement in the Editorial Manager submission system accurately describes where your code can be found. More information on our Code Policy, what and how to share can be found here: https://journals.plos.org/plosbiology/s/code-availability

Please note that we cannot accept sole deposition of code in GitHub, as this could be changed after publication. However, you can archive this version of your publicly available GitHub code to Zenodo. Once you do this, it will generate a DOI number, which you will need to provide in the Data Accessibility Statement (you are welcome to also provide the GitHub access information). See the process for doing this here: https://docs.github.com/en/repositories/archiving-a-github-repository/referencing-and-citing-content

------------------------------------------------------------------------

Reviewers' comments

Rev. 1: François Leulier - note that this reviewer has signed the review.

The revised version of the MS is addressing adequately my initial comments. I believe the MS is now more solid and make a more compelling case supporting the author's model.

Rev. 2:

The revised version has been substantially improved. The results are intriguing, as they reveal a direct effect of pathogenic bacteria on stem cell proliferation mediated through a PGRP-LC/PGRP-LE-Imd-p38 signaling pathway. Another unexpected finding is that peptidoglycan, the microbial elicitor sensed by these receptors, is not sufficient on its own to reproduce this effect, suggesting that both tissue damage and peptidoglycan are required to drive the stem cell response. Overall, the study is interesting and thought-provoking, although future additional work might be required to fully substantiate these conclusions. Several points, however, still need to be addressed.

Major points

-'null PGRP-LC[E12] mutants were not viable as homozygotes on our medium'. This is very strange because PGRP-LC[E12] is a viable mutation.

- Implication of Imd:

The authors report no effect of the imd[1] mutation on ISC proliferation (Figure S4A), in contrast to the phenotype observed upon stem cell-specific RNAi knockdown. This discrepancy is surprising and warrants further clarification. One possible explanation is the existence of compensatory mechanisms when Imd is impaired in all cells, but not when it is specifically depleted in stem cells. Alternatively, the imd[1] allele, a strong hypomorph, may not fully abolish Imd function in the context of p38 activation. This issue could be addressed by testing a bona fide null allele of imd, such as imd[shadok] (generated by the Ferrandon laboratory). In addition, Minos insertions in the imd locus that are likely null alleles could also be considered to strengthen the analysis.

Minor points:

- Introduction

'This increased ROS production eliminates invading pathogenic bacteria from the lumen (33, 41)' This statement has been recently contradicted in a reproducibility experiment. This should be mentioned (see Westlake et al Elife 2026 https://doi.org/10.7554/eLife.108404.1).

- Drosomysin should be Drosomycin

- 'This indicates that after adult flies consumed food containing DAP-type peptidoglycan or P.e., midgut ECs launched an innate immune response by producing AMPs.' This statement is not new and could be back up by references (ex among others Boscon-Drayon 2012; Neyen 2012)

- Use of Drs-GFP in the midgut: There is no evidence for a role of Toll pathway in the midgut to regulate AMPs and Drs is not induced in the gut (Buchon 2009). Thus, this experiment should be removed. The authors do not need to discuss the Toll pathway since the focus is on PGRP-LC/LE.

- I could not figure what is the meaning of 5d +-18h on the figure panels? Are they monitoring stem cell proliferation at 5 days post infection or at 18h post infection using 5 days old flies? This is important to clarify.

- Mode of infection: Is it continuous feeding or one shot? Those types of information should appear in the result section.

-Two points I raised here but that were not mentioned in my first review came to my mind. I would understand if the authors do not want to address them but I still consider them as important.

- Most observations are performed at a single time point (4 days or 18h). It would greatly strengthen the study to include a kinetic analysis of stem cell proliferation (e.g., using PH3 staining) to determine at which stage of epithelial renewal the Imd pathway is required. For instance, Liu et al. (Cell Reports) performed a time-course analysis at 16 h, 48 h, and 72 h. Such an approach would help clarify whether the Imd-dependent effect represents an early or late response.

- The observation that both PGRP-LE and PGRP-LC individually have strong effects is somewhat puzzling, as these receptors are generally thought to function independently. It would therefore be informative to analyze double mutants (PGRP-LE; PGRP-LC) to assess potential redundancy or additive effects between these pathways.

# Author comment: Author response to Decision Letter 3

**DOI:** 10.1371/journal.pbio.3003951.r007

29 Jul 2026

Attachment Submitted filename: response to reviewers_Uttekar_R3.docx

# Editor report: Decision Letter 3

**DOI:** 10.1371/journal.pbio.3003951.r008

**Authors:** Ines Alvarez-Garcia

31 Jul 2026

Dear Dr Patel,

Thank you for the submission of your revised Research Article entitled "Recognition of pathogenic bacteria by intestinal stem cells promotes adult Drosophila midgut regeneration" for publication in PLOS Biology. On behalf of my colleagues and the Academic Editor, Rebecca Drummond, I am delighted to let you know that we can in principle accept your manuscript for publication, provided you address any remaining formatting and reporting issues. These will be detailed in an email you should receive within 2-3 business days from our colleagues in the journal operations team; no action is required from you until then. Please note that we will not be able to formally accept your manuscript and schedule it for publication until you have completed any requested changes.

Please take a minute to log into Editorial Manager at http://www.editorialmanager.com/pbiology/, click the "Update My Information" link at the top of the page, and update your user information to ensure an efficient production process.

PRESS

We frequently collaborate with press offices. If your institution or institutions have a press office, please notify them about your upcoming paper at this point, to enable them to help maximise its impact. If the press office is planning to promote your findings, we would be grateful if they could coordinate with biologypress@plos.org. If you have previously opted in to the early version process, we ask that you notify us immediately of any press plans so that we may opt out on your behalf.

We also ask that you take this opportunity to read our Embargo Policy regarding the discussion, promotion and media coverage of work that is yet to be published by PLOS. As your manuscript is not yet published, it is bound by the conditions of our Embargo Policy. Please be aware that this policy is in place both to ensure that any press coverage of your article is fully substantiated and to provide a direct link between such coverage and the published work. For full details of our Embargo Policy, please visit http://www.plos.org/about/media-inquiries/embargo-policy/.

Many congratulations and thanks again for choosing PLOS Biology for publication and supporting Open Access publishing. We look forward to publishing your study.

Sincerely,

Ines

--

Ines Alvarez-Garcia, PhD

Senior Editor

PLOS Biology

ialvarez-garcia@plos.org
